# PTRF/Cavin-1 and MIF Proteins Are Identified as Non-Small Cell Lung Cancer Biomarkers by Label-Free Proteomics

**DOI:** 10.1371/journal.pone.0033752

**Published:** 2012-03-26

**Authors:** Angelo Gámez-Pozo, Iker Sánchez-Navarro, Enrique Calvo, María Teresa Agulló-Ortuño, Rocío López-Vacas, Esther Díaz, Emilio Camafeita, Manuel Nistal, Rosario Madero, Enrique Espinosa, Juan Antonio López, Juan Ángel Fresno Vara

**Affiliations:** 1 Laboratory of Molecular Pathology & Oncology, Instituto de Genética Médica y Molecular, Hospital Universitario La Paz, Madrid, Spain; 2 Service of Proteomics, Centro Nacional de Investigaciones Cardiovasculares, Madrid, Spain; 3 Service of Oncology, Instituto de Investigación Sanitaria i+12, Hospital Doce de Octubre, Madrid, Spain; 4 Service of Pathology, Instituto de Investigación Sanitaria IdiPAZ, Hospital Universitario La Paz, Madrid, Spain; 5 Statistics Department, Instituto de Investigación Sanitaria IdiPAZ, Hospital Universitario La Paz, Madrid, Spain; 6 Service of Medical Oncology, Instituto de Investigación Sanitaria IdiPAZ, Hospital Universitario La Paz, Madrid, Spain; Ospedale Pediatrico Bambino Gesu', Italy

## Abstract

With the completion of the human genome sequence, biomedical sciences have entered in the “omics” era, mainly due to high-throughput genomics techniques and the recent application of mass spectrometry to proteomics analyses. However, there is still a time lag between these technological advances and their application in the clinical setting. Our work is designed to build bridges between high-performance proteomics and clinical routine. Protein extracts were obtained from fresh frozen normal lung and non-small cell lung cancer samples. We applied a phosphopeptide enrichment followed by LC-MS/MS. Subsequent label-free quantification and bioinformatics analyses were performed. We assessed protein patterns on these samples, showing dozens of differential markers between normal and tumor tissue. Gene ontology and interactome analyses identified signaling pathways altered on tumor tissue. We have identified two proteins, PTRF/cavin-1 and MIF, which are differentially expressed between normal lung and non-small cell lung cancer. These potential biomarkers were validated using western blot and immunohistochemistry. The application of discovery-based proteomics analyses in clinical samples allowed us to identify new potential biomarkers and therapeutic targets in non-small cell lung cancer.

## Introduction

Lung cancer is the leading cause of cancer death in the world. The overall survival rate at 5 years is 15% and has not been improved for decades. Two thirds of patients are diagnosed with advanced disease where therapeutic options are palliative, and up to 55% of patients with limited disease eventually relapse after radical surgery [1].

Gene expression profiling has led to the identification of groups of patients with different outcome, thus reflecting the heterogeneity of this disease [2]. However, gene-level analyses do not detect subtle changes caused by post-translational modifications of proteins [3]. A deep understanding of the processes of carcinogenesis, tumor progression and metastasis requires the analysis of both the genome and the proteome [4]. Proteomic technologies based on mass spectrometry (MS) have emerged as preferred components of a strategy to discover diagnostic, prognostic and therapeutic protein biomarkers [5]. Continuing advances in this field give this strategy an enormous potential for such investigations [6], [7].

Recent clinical trials demonstrating good response to new drugs in specific subgroups of patients underline the need for molecular tests that complement classical histopathological procedures [8]. In this context, proteomic profiling can provide valuable biomarker tools for efficient patient stratification and therapy selection.

Although it is possible to analyze proteins from tissues using mass spectrometry [3], [9], the complexity of the clinical sample and the amount of available protein are limiting factors. Therefore, sample enrichment in biologically relevant analytes is required [5]. Most eukaryotic cellular processes are regulated by protein phosphorylation, and deregulation of this key post-translational modification is common in cancer and other diseases. This explains why protein kinases have emerged as the main class of new drug targets in oncology and other fields [10]. In this work we have applied phosphopeptide enrichment coupled with label-free MS techniques to identify already known and new potential biomarkers in non-small cell lung cancer clinical tissues and validate them using western blot and immunohistochemistry.

## Materials and Methods

### Ethics statement

Institutional approval from our ethical committee was obtained for the conduct of the study (Comité Ético de Investigación Clínica, Hospital Universitario La Paz). Data were analyzed anonymously. Patients provided written consent so that their samples and clinical data could be used for investigational purposes.

### Sample selection

Frozen samples from patients diagnosed with lung cancer were retrieved from the Department of Pathology of Hospital Universitario La Paz (Madrid, Spain): 5 lung adenocarcinoma (AC), 5 lung squamous cell carcinoma (SC) and 5 normal lung (NL) samples. The histopathological features of each sample were reviewed by an experienced lung pathologist to confirm diagnosis and tumor content. At least 50% of a sample had to be made up of tumor cells for it to be eligible. Samples from patients were kindly provided by the IdiPAZ Biobank (RD09/0076/00073) integrated in the Spanish Hospital Biobanks Network (RetBioH; www.redbiobancos.es). Samples were registered and processed following current procedures and fixed/frozen immediately after their reception.

### Total protein extraction, solubilization and digestion

Samples were cut in a Leica CM3050S cryostat, obtaining 10 sections of 10 microns thickness each. Tissue was processed with TRIzol reagent (Invitrogen) following the manufacturer's instructions. For MS analyses, protein pellets were resuspended in guanidine hydrochloride 6 M and heated 10 minutes at 95°C with agitation. Subsequently, 950 µl of 50 mM ammonium bicarbonate (pH 7–9) per sample were added. Protein sample concentration was measured by MicroBCA Protein Assay Kit (Pierce-Thermo Scientific). Trypsin MS Grade Gold (Promega) was added to each sample to a 1∶50 relation. Digestion was carried out overnight at 37°C. The digested sample was divided into two aliquots.

### Parallel IMAC (PIMAC)

Phosphopeptide enrichment was carried out as described previously [11]. Briefly, Fe(III)-based IMAC was performed in one aliquot of digested protein using the PHOS-Select Iron Affinity Gel (Sigma-Aldrich) following the manufacturer's instructions. Ga(III)-based IMAC was performed in another aliquot of digested protein using the Phosphopeptide Isolation Kit (Pierce-Thermo Scientific) following the manufacturer's instructions. Eluates were mixed, vacuum-dried and stored at −20°C for later MS analysis.

### LC-MS/MS analyses

Peptide mixtures were subjected to nano-liquid chromatography coupled with MS for protein identification. Peptides were injected into a C-18 reversed phase (RP) nano-column (100 µm I.D. and 12 cm, Mediterranea sea, Teknokroma) and analyzed in a continuous acetonitrile gradient consisting of 0–40% B in 90 min, 50–90% B in 20 min (B = 95% acetonitrile, 0.5% acetic acid). At the end of the gradient, the column was washed with 90% B and equilibrated with 5% B for 20 min. A flow rate of 300 nl/min was used to elute peptides from the RP nano-column to an emitter nanospray needle for real time ionization and peptide fragmentation on an LTQ-Orbitrap XL mass spectrometer (Thermo-Fisher). An enhanced FT-resolution spectrum (resolution = 60000) followed by the MS/MS spectra from the five most intense parent ions were analyzed along the chromatographic run (130 min). Dynamic exclusion was set at 1 min. For protein identification fragmentation spectra were searched against the MSDB database (version 091509) using the Mascot 2.1 program (Matrixscience). Two missed cleavages were allowed, and an error of 10 ppm or 0.8 Da was set for full MS or MS/MS spectra searches, respectively. All identifications were performed by Proteome Discoverer 1.0 software (Thermo-Fisher). Decoy database search for false discovery rate analysis was set at 0.05 by applying corresponding filters. Raw data files were processed and compared with SIEVE version 1.2 (Thermo-Fisher). Protein identifications were validated using the BLAST tool from the blastp suite (http://blast.ncbi.nlm.nih.gov). For detailed peptide mass fingerprint and protein identification settings, see Table S4.

### Inmunoblotting assays

For inmunoblotting assays, protein pellets were resuspended in 2% SDS and heated 10 minutes at 95°C with agitation. Protein sample concentration was measured by MicroBCA Protein Assay Kit (Pierce-Thermo Scientific).Western blots were performed using WesternDot system (Invitrogen) in a SNAP i.d. device (Millipore). MIF antibody 1∶ 250 dilution (R&D Systems) and PTRF antibody 1∶125 dilution (BD Biosciences) were used. Densitometry analyses were performed using ImageJ 1.38e software (http://rsb.info.nih.gov/ij/) to measure the intensity of bands. For western blot normalization, total protein loading was measured using the Novex Reversible Membrane Stain Kit (Invitrogen).

### Immunohistochemistry

Formalin-fixed, paraffin-embedded tissue blocks, representative of normal lung and non-small cell lung cancer diagnosis, were retrieved following routine histopathological assessment. Sections were processed using a Dako Autostainer universal staining system (Dako). For this study, 3.5-µm sections were immunostained with anti-MIF 1∶2000 (R&D Systems) or anti-PTRF 1∶100 (BD Biosciences). Images were obtained in a Leyca microscope with magnification ×40. The percentage of stained tissue and the stain intensity (0, +, ++ or +++) was obtained for each sample and marker evaluated. IHC staining was considered positive when at least 50% of the tissue (normal or tumoral) was stained with at least ++.

### Statistical Analyses

Expression values between sample groups were compared using a Kruskal-Wallis test (Gaussian Approximation). To assess differences between pairs of groups Dunn's Multiple Comparison Test was used. A p-value <0.05 was considered significant. SIEVE and densitometry values were compared using Pearson's correlation coefficient.

### Bioinformatics

Protein lists were processed using The Database for Annotation, Visualization and Integrated Discovery (DAVID) version 2.0 (http://david.abcc.ncifcrf.gov/home.jsp) [12], [13]. To identify under- and over-represented functional categories we used Protein ANalysis THrough Evolutionary Relationships (PANTHER) database v 6.1 (www.pantherdb.org) [14]. Tumor protein list were compared to the normal lung list using the binomial test [15] for each molecular function, biological process or pathway term in PANTHER. Protein-protein interactions were obtained from the Search Tool for the Retrieval of Interacting Genes/Proteins (STRING) database v9.0 containing known and predicted physical and functional protein-protein interactions [16]. STRING in protein mode was used, and only interactions based in experimental protein-protein interaction and curated databases- with confidence levels over 0.5- were kept.

## Results

In this study, we assessed differences at the protein level between non-small cell lung cancer (NSCLC) and lung normal tissue using a phosphopeptide enrichment strategy and a label-free approach. Samples were analyzed on a LTQ-Orbitrap XL after being subjected to liquid chromatography. Since it is known that different techniques isolate distinct and overlapping segments of the phosphoproteome [17], including Fe(III) and Ga(III) IMAC [18], we mixed the Fe(III) and Ga(III) IMAC fractions from each sample and analyzed them together.

We evaluated the number of unique peptides and their corresponding proteins, as well as phosphopeptides and their corresponding phosphoproteins, identified in lung adenocarcinoma (AC), lung squamous cell carcinoma (SC) and normal lung (NL) samples applying a decoy database search at false discovery rate<0.05. The extensive analysis performed in NSCLC and NL samples using LC-MS/MS allowed us to identify a mean of 381 unique peptides per sample, of which a mean of 56 were phosphopeptides. These peptides corresponded to a mean of 138 unique proteins identified per sample, of which a mean of 39 were phosphorylated. The fraction of phosphopeptides identified (number of phosphorylated peptides*100/number of identified peptides) was 19.9%.

**Figure 1:**
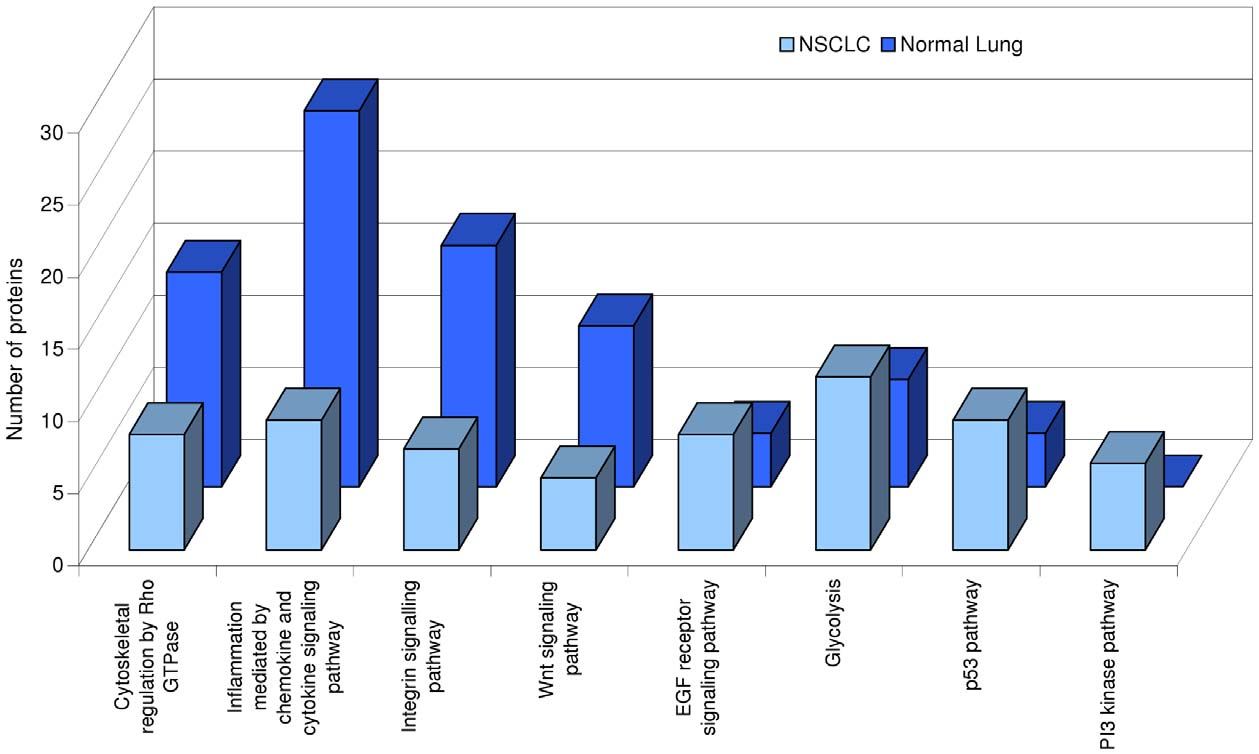
Analysis of differences in GO Pathways between NSCLC and normal lung. Comparison of number of proteins assigned to each GO pathway category. Normal tissue sample categories are represented as fold-change in relation to this category. Statistical significance is tested using the binomial test. Only significant categories (p<0.05) are shown.

Gene ontology analyses were performed using all identified proteins. The tumor protein list was compared to the normal lung protein list for each molecular function (Figure S1), biological process (Figure S2), or pathway (Figure 1) terms using PANTHER. This approach showed significant differences between normal lung and tumor samples (complete analyses are provided in Table S1). Differences in molecular functions are mainly related to the interaction with nucleic acids and the regulation of protein synthesis and activity. Processes controlling exocytosis, immune response, response to stimulus, response to stress and transport were significantly under-represented in tumors, whereas categories related to cell-matrix adhesion or response to toxin were over-represented. On the other hand, homeostasis categories were under-represented, whereas categories related with energy production and cell proliferation were over-represented in tumors. Remarkable differences in pathway analysis appeared in categories related with signal transduction control. While cytoskeletal regulation by Rho GTPase, inflammation mediated by chemokine and cytokine signaling pathway, integrin signaling pathway and Wnt signaling pathway were under-represented in tumor samples, EGF receptor signaling pathway, Glycolysis, p53 pathway and PI3 kinase pathway were over-represented.

Differential expression analysis between NSCLC vs. normal lung was performed using SIEVE 1.2 software. A total of 296 differentially expressed m/z peaks were found, 115 of which had available MS2 spectra, leading to the identification of proteins differentially expressed between normal lung and NSCLC samples (Table 1). All data obtained from SIEVE analyses, including relative expression values, are provided in Table S2.

**Table 1:** Differentially expressed peptides.

Peptide	m/z	GeneID	Gene Symbol	Ratio TvsN	P Value
**Peptides down-regulated in tumor samples**					
FKDLGEENFK	409.54	213	ALB	0.55	0.039
VLSPADKTNVK	586.34	3040	HBA2	0.10	0.016
VGAHAGEYGAEALER	765.37	3040	HBA2	0.19	0.000
TYFPHFDLSHGSAQVK	917.45	3040	HBA2	0.26	0.000
TYFPHFDLSHGSAQVK	611.97	3040	HBA2	0.33	0.000
EFTPQVQGAFQK	690.36	3043	HBB	0.23	0.010
VVAGVANALAHKYH	725.40	3043	HBB	0.24	0.003
VLGAFSDGLAHLDNLK	835.45	3043	HBB	0.24	0.019
LHVDPENFR	563.79	3043	HBB	0.25	0.008
VVAGVANALAHK	575.34	3043	HBB	0.29	0.003
KVLGAFSDGLAHLDNLK	600.00	3043	HBB	0.29	0.005
VNVDEVGGEALGR	438.89	3043	HBB	0.32	0.000
VNVDEVGGEALGR	657.84	3043	HBB	0.33	0.000
VLGAFSDGLAHLDNLK	557.30	3043	HBB	0.34	0.007
SLKESEALPEK	615.83	284119	PTRF	0.06	0.004
SLKESEALPEK	410.89	284119	PTRF	0.12	0.004
**Peptides up-regulated in tumor samples**					
VAPEEHPVLLTEAPLNPK	652.03	60	ACTB	2.32	0.026
MQKEITALAPSTMK	516.94	60	ACTB	2.40	0.032
IWHHTFYNELR	505.92	60	ACTB	2.50	0.024
IMFVDPSLTVR	639.35	10551	AGR2	4.70	0.024
LPQTLSR	407.74	10551	AGR2	5.99	0.021
KLNQALLDLHALGSAR	574.00	2512	FLP	5.93	0.047
PPYTVVYFPVR	669.31	2950	GSTP1	2.64	0.039
VGVNGFGR	403.22	2597	GAPDH	3.67	0.005
GALQNIIPASTGAAK	706.40	2597	GAPDH	7.52	0.009
DNIQGITKPAIR	442.59	8294, 8359, 8364, 8367	HIST1H4A, HIST1H4C, HIST1H4E, HIST1H4I	2.36	0.001
AGLQFPVGR	472.77	3012, 3013, 3014, 3015, 8329, 8331, 8334, 8337, 92815, 94239, 55766, 85235, 221613	HIST1H2AA, HIST1H2AC, HIST1H2AD, HIST1H2AE, HIST1H2AH, HIST1H2AI, HIST1H2AJ, HIST2H2AA3, H2AFJ, H2AFV, H2AFX, H2AFZ, HIST3H2A	2.52	0.001
TVTAMDVVYALKR	489.61	8294, 8359, 8364, 8367	HIST1H4A, HIST1H4C, HIST1H4E, HIST1H4I	2.68	0.009
DNIQGITKPAIR	663.38	8294, 8359, 8364, 8367	HIST1H4A, HIST1H4C, HIST1H4E, HIST1H4I	2.73	0.004
YHTSQSGDEMTSLSEYVSR	726.32	3326	HSP90AB1	3.36	0.002
ALLFIPR	415.27	3326	HSP90AB1	4.50	0.004
SNMDNMFESYINNLRR	668.64	3856	KRT8	2.63	0.041
TKFETEQALR	408.22	3872/3880	KRT17/KRT19	1.78	0.027
LLEGEDAHLTQYK	506.26	3872/3881	KRT17/KRT19	2.67	0.007
VLDELTLAR	515.30	3872/3882	KRT17/KRT19	2.75	0.004
PMFIVNTNVPR	644.35	4282	MIF	21.37	0.000
LRTLNLGGNALDR	706.90	60506	NYX	4.99	0.008
WFYIASAFR	580.80	5005	ORM2	3.08	0.015
ALESPERPFLAILGGAK	590.34	5230	PGK	5.51	0.003
SLPNEEIVQK	578.82	182465	SON	13.52	0.004
GYPTLLWFR	576.81	81567	TXNDC5	3.78	0.032
TLMNLGGLAVAR	608.35	8407	TAGLN2	2.97	0.035
QMEQISQFLQAAER	560.28	8407	TAGLN2	3.31	0.012
DDGLFSGDPNWFPKK	574.94	8407	TAGLN2	3.64	0.020
DDGLFSGDPNWFPK	797.86	8407	TAGLN2	4.78	0.035
LAVNMVPFPR	572.32	203068	TUBB	3.23	0.010
LHFFMPGFAPLTSR	540.95	203068	TUBB	4.53	0.003

Differentially expressed peptides between NSCLC and normal lung samples identified using SIEVE 1.2 software. Peptides presenting different m/z values have been identified with various charge states.

**Figure 2:**
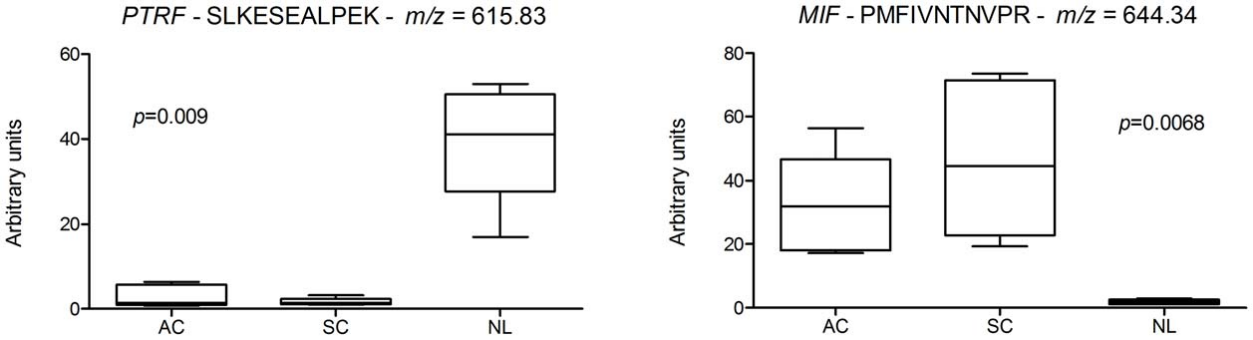
PTRF/cavin-1 and MIF label-free expression values by SIEVE. Boxplots represent mean and 25th–75th percentile; whiskers represent minimun and maximun. Measurements were obtained from five different samples in each condition. Kruskall-Wallis test p-values are shown. AC: Adenocarcinoma; SC: Squamous cell carcinoma; NL: Normal lung.

**Figure 3:**
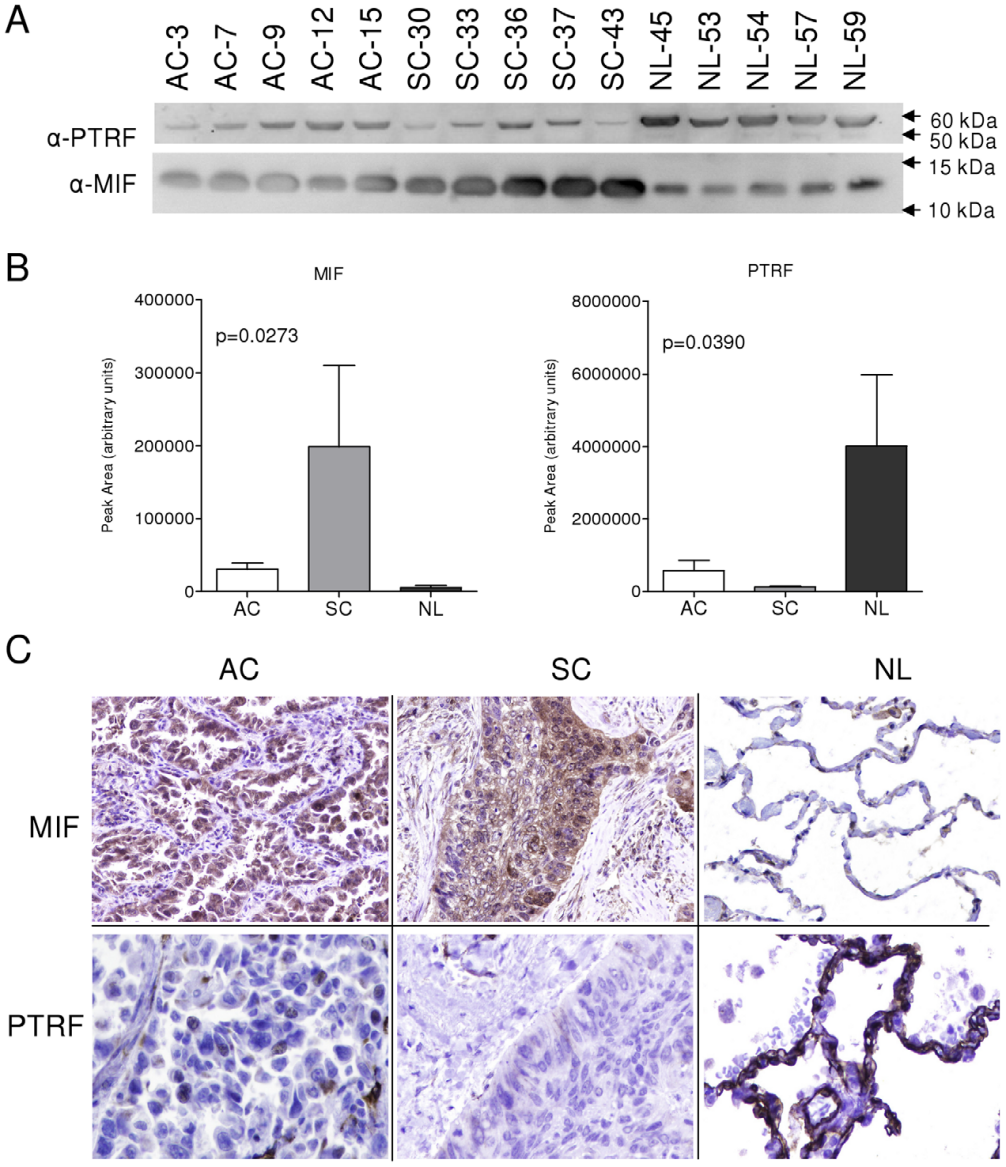
Validation of PTRF/cavin-1 and MIF expression changes using IHC and western blot. a) Western blot of total protein extracted from indicated samples, using anti-MIF and anti-PTRF/cavin-1 primary antibodies. b) Densitometric analyses of western blot. ImageJ 1.38e software was employed to measure the intensity of bands. All values in arbitrary units. c) Immunohistochemistry of indicated samples, using anti-MIF and anti-PTRF/cavin-1 primary antibodies. AC: Adenocarcinoma; SC: Squamous cell carcinoma; NL: Normal lung.

**Figure 4:**
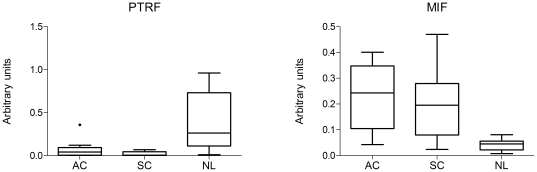
Validation of PTRF/cavin-1 and MIF expression changes by western blot using a new cohort. Box-Plot graphs showing PTRF and MIF western blot quantification using ImageJ 1.38e software. All values in arbitrary units. Each Box includes values from nine different samples. Differences between normal and tumoral samples were p<0.005 in both cases (Kruskall-Wallis test).

PTRF/cavin-1 and MIF outstand among the differentially expressed biomarkers between NSCLC and normal lung samples in label-free analyses as the most down-regulated and up-regulated respectively (Figure 2). PTRF/cavin-1 showed loss of expression in both adenocarcinoma and squamous cell carcinoma samples. On the other hand, MIF showed an increased expression in these samples. In order to avoid false positive identifications, more than twenty MS2 spectra for each MIF and PTRF/cavin-1 peptides were evaluated manually (Figures S3 and S4 and Table S3). Changes in PTRF/cavin-1 and MIF expression in NSCLC samples were validated using immunohistochemistry (IHC) and western blot analyses. The same samples used for MS/MS analyses and nine additional samples of adenocarcinoma, squamous cell carcinoma and normal lung were evaluated for MIF and PTRF using western blot. Five additional samples of adenocarcinoma, squamous cell carcinoma and normal lung were evaluated for MIF and PTRF using IHC. All tumours show positive staining for MIF and negative staining for PTRF, while all normal tissues were negative for MIF and positive for PTRF staining. MIF over-expression in tumor tissues was confirmed both by IHC and western blot (Figures 3 and 4). On the other hand, tumor samples confirmed loss of PTRF/cavin-1 expression when compared with normal lung (Figures 3 and 4) using both techniques. Pearson's correlation between Sieve label-free expression values and western blot quantification was r = 0.723 and r = 0.754 (p<0.005) for MIF and PTRF/cavin-1 respectively.

**Figure 5:**
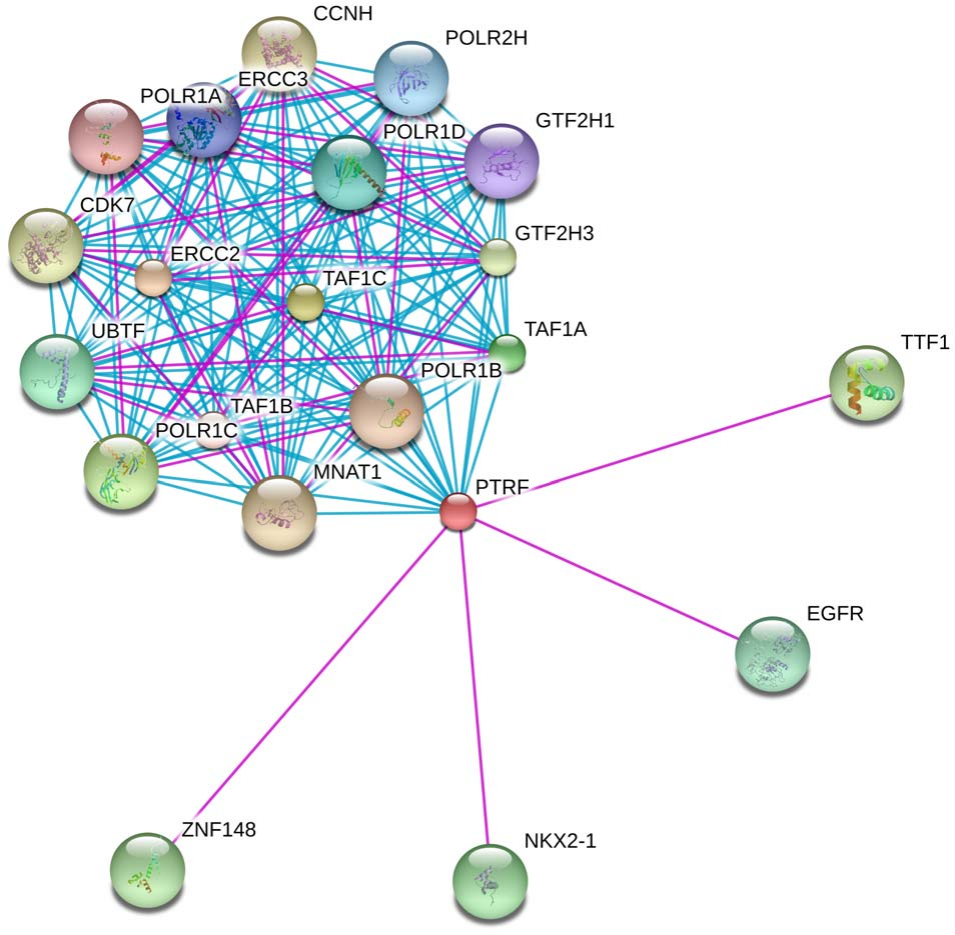
PTRF interactome network. A graph of PTRF network built using STRING v9.0 is shown. Different line colours represent the types of evidence for the association: pink for experiments and blue for databases.

**Figure 6:**
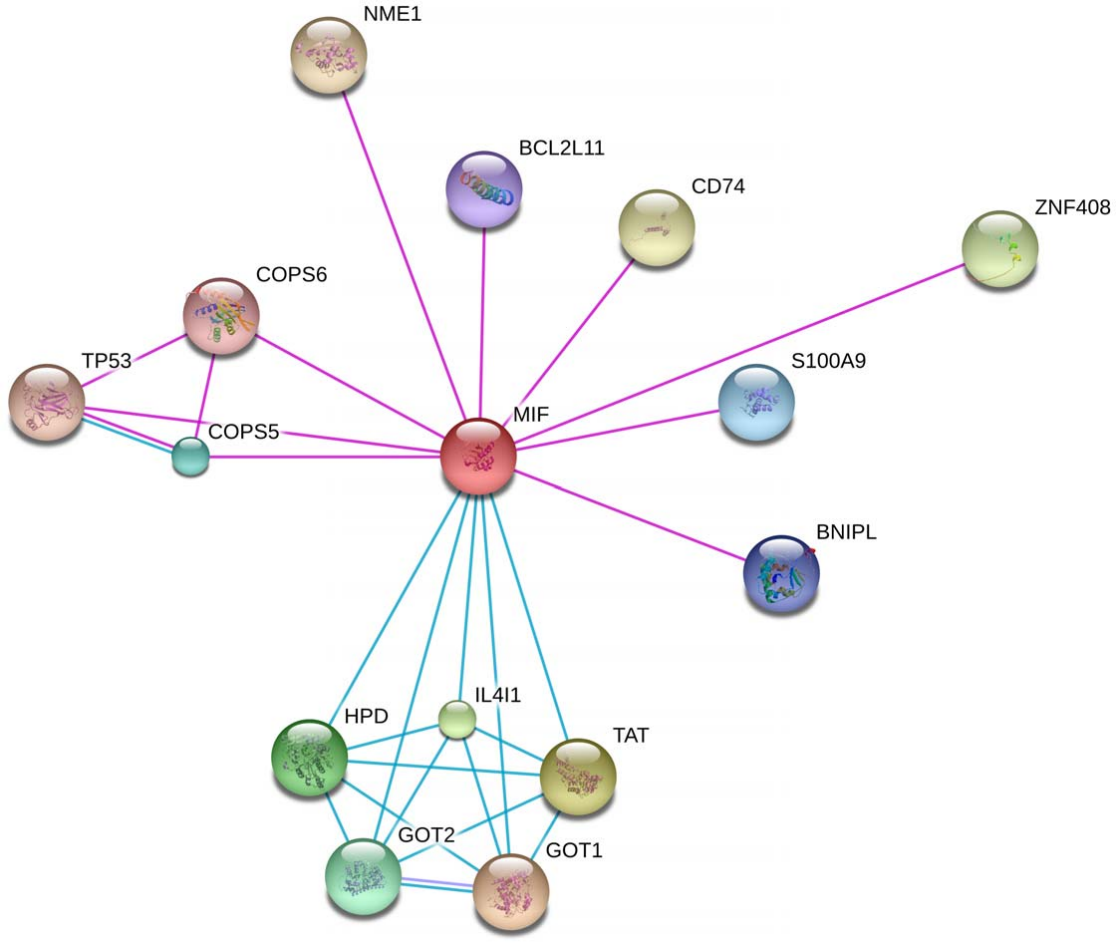
MIF interactome network. A graph of MIF network built using STRING v9.0 is shown. Different line colours represent the types of evidence for the association: pink for experiments and blue for databases.

We searched in the STRING database for interactomic connections of MIF and PTRF. In order to minimize the rate of false positives, we eliminated partners using stringent criteria, and only experimental protein-protein interactions and pathways from curated databases were taken into account. PTRF is included in the RNA transcription pathway, and physically interacts with TTF1. Other PTRF interactions comprise proteins involved in transcription regulation and EGFR (Figure 5). MIF is related to the phenylalanine metabolism pathway, and interacts with p53 and proteins of the COP9 signalosome complex, a complex involved in various cellular and developmental processes, including p53 phosphorylation-mediated degradation [19]. Other interactions comprise proteins related with cell death regulation and inflammatory process (Figure 6).

## Discussion

Proteomics in general and phosphoproteomics in particular are becoming the preferred methods of protein discovery-based analyses. The use of label-free, discovery-based approaches may help discover unexpected biological connections due to the absence of previous knowledge bias. Bioinformatics tools, such as gene ontology and interactome analyses, applied on clinical samples have great potential to identify pathways and molecules with implication at the therapeutic level and may offer clues to the genesis of diseases and their underlying molecular alterations. However, both the technology itself and data analysis tools should be further refined before their entry into the clinic.

Phosphopeptide enrichment of samples prior to MS analysis using PIMAC worked reasonably well, as 20% of measured peptides were phosphorylated. Previous studies have shown an enrichment of phosphopeptides of approximately 50% using an IMAC protocol similar to ours on tryptic digest of a mixture of several reference proteins [20]. Considering that our samples were very complex and that we did not use any fractionation step, phosphopeptide enrichment was successful and comparable with that obtained in previous works [21], [22]. However, most of the spectra showing a phosphate loss presented a poor fragmentation, and no peptide identification was generated. The use of new fragmentation techniques, as higher energy collisional dissociation, improve the quality of fragmentation spectra [23], allowing to perform large-scale phosphoproteome analysis.

Gene ontology analyses of biological process and pathways showed an increase in categories related to energy production in cells, such as glycolysis and generation of precursor metabolites and energy. These differences in energy metabolism between normal and tumor cells are known as Warburg effect [24]. From the signaling pathways under-represented in NSCLC tissues, chemokine- and cytokine-mediated inflammation has been previously shown to be under-represented in NSCLC [25]. It is remarkable the over-representation of proteins belonging to EGFR signaling pathway, in a context where the clinical use of EGFR inhibitors has become the paradigm of personalized therapy for NSCLC [26], [27], [28].

More than 10% of detected peptides showed a differential expression between normal and tumor samples. The percentage of differential peptides was less than 2% when comparing adenocarcinoma and lung squamous cell carcinoma samples, but still there were substantial differences between these two NSCLC histological subtypes, as we have previously demonstrated [11].

We were able to validate NSCLC potential biomarkers identified in shotgun proteomics analyses using IHC and western blot approaches. MIF (macrophage migration inhibitory factor) discriminated between normal lung and NSCLC samples. This well known factor is a proinflammatory cytokine capable of acting as soluble growth factor, expressed and secreted in response to mitogens and integrin-mediated signals. MIF protein is involved in many malignancies, as it promotes cellular transformation, inhibits cytolytic immune response against tumor cells and promotes neovascularization [29]. Interactome analyses revealed a close relation between cell death regulation and MIF, and it is not surprising that MIF over-expression was described in many types of cancer, including colorectal, breast, prostate, skin and lung cancer [30], [31], having a major role in the development of tumors in the central nervous system [32]. Tumors co-expressing MIF and its membrane receptor (CD74 protein) have increased vascularization [33]. Although there are several molecules that inhibit enzymatic activity of MIF, its high IC50 has limited its clinical use so far [34], but new molecules are under current development [35]. Our results show an increased expression of MIF in NSCLC samples by label-free proteomics, confirmed by both western blot and immunohistochemistry.

PTRF (Polimerase I and Transcript Release Factor), also known as cavin-1, is a protein essential for RNA transcription [36] and caveolae formation [37]. These invaginations of the cell surface are associated with processes of vesicular transport, cholesterol homeostasis, signal transduction [38] and lipolysis control [39]. Therefore, it is not surprising that PTRF/cavin-1 mutations are associated with congenital generalized lipodystrophy, type 4 in humans [40]. PTRF/cavin-1 colocalizes with caveolin 1 (CAV1) within caveolae [41], and positively modulates its expression [42]. Interactome analyses suggest that PTRF harbors unknown functions beyond some recently described [43], [44], [45]. Loss of PTRF/cavin-1 expression in prostate cancer has been related with progression [46], and it has been demonstrated that its expression decreases the migration of PTRF/cavin-1-deficient prostate cancer cells [47]. The loss of PTRF/cavin-1 expression in tumorigenic HBE cells as compared with normal human bronchial epithelial cells has been proved recently [48]. Bai and colleagues have reported recently that PTRF protein was down-regulated in breast cancer cell lines and breast tumor tissue, and that down-regulation of PTRF in breast cancer cells was associated with the promoter methylation [49]. PTRF/cavin-1 phosphorylated species have been described in cells that over-express EGFR, which suggests a function in this signaling pathway [50]. Our label-free proteomics results indicate that PTRF expression is lost in NSCLC samples. These results were confirmed using both western blot and immunohistochemical staining. This is the first study showing PTRF/cavin-1 loss of expression in NSCLC tumor tissue at the protein level. This loss of expression, along with PTRF-EGFR interaction and EGFR pathway deregulation in NSCLC samples, suggests a role of PTRF in NSCLC development.

Our work demonstrates that it is possible to identify potential biomarkers using a label-free differential proteomics strategy on real clinical samples. We identified several differential markers, two of which were validated by alternative classical proteomic methods. Moreover, we show that gene ontology and interaction analyses can identify pathways and processes altered on tumor tissue, which may provide clues to the genesis of the disease and its underlying molecular alterations, and could be susceptible to therapeutic intervention. In this sense, this work indicates that PTRF role in NSCLC and its relationship with EGFR pathway deserves further exploration.

## Supporting Information

Figure S1 **Analysis of differences in GO Molecular Function between NSCLC and normal lung.** Comparison of number of proteins assigned to each GO pathway category. Normal tissue sample categories are represented as fold-change in relation to this category. Statistical significance is tested using the binomial test. Only significant categories (p<0.05) are shown.
(TIF)

Figure S2 **Analysis of differences in GO Biological Process between NSCLC and normal lung.** Comparison of number of proteins assigned to each GO pathway category. Normal tissue sample categories are represented as fold-change in relation to this category. Statistical significance is tested using the binomial test. Only significant categories (p<0.05) are shown.
(TIF)

Figure S3 **Fragmentation spectra from PTRF SLKESEALPEK tryptic peptide.** Diagram shows fragment ions corresponding to main fragmentation series (b-amino and y-carboxy). * indicates water loss; 2+, doubly charged fragment. Parental ion is marked with an arrow.
(TIF)

Figure S4 **Fragmentation spectra from MIF PMFIVNTNVPR tryptic peptide.** Diagram shows fragment ions corresponding to main fragmentation series (b-amino and y-carboxy). * indicates water loss. Parental ion is marked with an arrow.
(TIF)

Table S1 **Gene Ontology analyses performed with PANTHER.** Normal lung protein list was used as reference list.
(PDF)

Table S2 **SIEVE label-free quantification.** Data obtained from SIEVE analyses, including relative expression values.
(PDF)

Table S3 **PTRF and MIF MS2 spectra.**
(PDF)

Table S4 **Peptide Mass Fingerprint and Protein Identification settings.**
(DOC)

## References

[1] Mountain CF (1997). Revisions in the International System for Staging Lung Cancer.. Chest.

[2] Shedden K, Taylor JM, Enkemann SA, Tsao MS, Yeatman TJ (2008). Gene expression-based survival prediction in lung adenocarcinoma: a multi-site, blinded validation study.. Nat Med.

[3] Groseclose MR, Massion PP, Chaurand P, Caprioli RM (2008). High-throughput proteomic analysis of formalin-fixed paraffin-embedded tissue microarrays using MALDI imaging mass spectrometry.. Proteomics.

[4] Conrad DH, Goyette J, Thomas PS (2008). Proteomics as a method for early detection of cancer: a review of proteomics, exhaled breath condensate, and lung cancer screening.. J Gen Intern Med.

[5] Hanash S (2003). Disease proteomics.. Nature.

[6] Marko-Varga G, Ogiwara A, Nishimura T, Kawamura T, Fujii K (2007). Personalized medicine and proteomics: lessons from non-small cell lung cancer.. J Proteome Res.

[7] Pastwa E, Somiari SB, Czyz M, Somiari RI (2007). Proteomics in human cancer research.. Proteomics Clin Appl.

[8] Shepherd FA, Rodrigues Pereira J, Ciuleanu T, Tan EH, Hirsh V (2005). Erlotinib in previously treated non-small-cell lung cancer.. N Engl J Med.

[9] Stoeckli M, Chaurand P, Hallahan DE, Caprioli RM (2001). Imaging mass spectrometry: a new technology for the analysis of protein expression in mammalian tissues.. Nat Med.

[10] Cohen P (2002). Protein kinases–the major drug targets of the twenty-first century?. Nat Rev Drug Discov.

[11] Gamez-Pozo A, Sanchez-Navarro I, Nistal M, Calvo E, Madero R (2009). MALDI profiling of human lung cancer subtypes.. PLoS One.

[12] Dennis G, Sherman BT, Hosack DA, Yang J, Gao W (2003). DAVID: Database for Annotation, Visualization, and Integrated Discovery.. Genome Biol.

[13] Huang DW, Sherman BT, Lempicki RA (2009). Systematic and integrative analysis of large gene lists using DAVID bioinformatics resources.. Nat Protoc.

[14] Thomas PD, Campbell MJ, Kejariwal A, Mi H, Karlak B (2003). PANTHER: a library of protein families and subfamilies indexed by function.. Genome Res.

[15] Cho RJ, Campbell MJ (2000). Transcription, genomes, function.. Trends Genet.

[16] Jensen LJ, Kuhn M, Stark M, Chaffron S, Creevey C (2009). STRING 8–a global view on proteins and their functional interactions in 630 organisms.. Nucleic Acids Res.

[17] Bodenmiller B, Mueller LN, Mueller M, Domon B, Aebersold R (2007). Reproducible isolation of distinct, overlapping segments of the phosphoproteome.. Nat Methods.

[18] Aryal UK, Ross AR (2010). Enrichment and analysis of phosphopeptides under different experimental conditions using titanium dioxide affinity chromatography and mass spectrometry.. Rapid Commun Mass Spectrom.

[19] Bech-Otschir D, Kraft R, Huang X, Henklein P, Kapelari B (2001). COP9 signalosome-specific phosphorylation targets p53 to degradation by the ubiquitin system.. EMBO J.

[20] Jensen SS, Larsen MR (2007). Evaluation of the impact of some experimental procedures on different phosphopeptide enrichment techniques.. Rapid Commun Mass Spectrom.

[21] Han G, Ye M, Zhou H, Jiang X, Feng S (2008). Large-scale phosphoproteome analysis of human liver tissue by enrichment and fractionation of phosphopeptides with strong anion exchange chromatography.. Proteomics.

[22] Sugiyama N, Masuda T, Shinoda K, Nakamura A, Tomita M (2007). Phosphopeptide enrichment by aliphatic hydroxy acid-modified metal oxide chromatography for nano-LC-MS/MS in proteomics applications.. Mol Cell Proteomics.

[23] Nagaraj N, D'Souza RC, Cox J, Olsen JV, Mann M (2010). Feasibility of large-scale phosphoproteomics with higher energy collisional dissociation fragmentation.. J Proteome Res.

[24] Warburg O (1956). On the origin of cancer cells.. Science.

[25] Bremnes RM, Al-Shibli K, Donnem T, Sirera R, Al-Saad S (2010). The Role of Tumor-Infiltrating Immune Cells and Chronic Inflammation at the Tumor Site on Cancer Development, Progression, and Prognosis: Emphasis on Non-small Cell Lung Cancer.. J Thorac Oncol.

[26] Ciardiello F, Tortora G (2008). EGFR antagonists in cancer treatment.. N Engl J Med.

[27] Gazdar AF (2010). Epidermal growth factor receptor inhibition in lung cancer: the evolving role of individualized therapy.. Cancer Metastasis Rev.

[28] Ray M, Salgia R, Vokes EE (2009). The role of EGFR inhibition in the treatment of non-small cell lung cancer.. Oncologist.

[29] Mitchell RA (2004). Mechanisms and effectors of MIF-dependent promotion of tumourigenesis.. Cell Signal.

[30] Lee H, Rhee H, Kang HJ, Kim HS, Min BS (2008). Macrophage migration inhibitory factor may be used as an early diagnostic marker in colorectal carcinomas.. Am J Clin Pathol.

[31] Rendon BE, Roger T, Teneng I, Zhao M, Al-Abed Y (2007). Regulation of human lung adenocarcinoma cell migration and invasion by macrophage migration inhibitory factor.. J Biol Chem.

[32] Bach JP, Deuster O, Balzer-Geldsetzer M, Meyer B, Dodel R (2009). The role of macrophage inhibitory factor in tumorigenesis and central nervous system tumors.. Cancer.

[33] McClelland M, Zhao L, Carskadon S, Arenberg D (2009). Expression of CD74, the receptor for macrophage migration inhibitory factor, in non-small cell lung cancer.. Am J Pathol.

[34] Winner M, Meier J, Zierow S, Rendon BE, Crichlow GV (2008). A novel, macrophage migration inhibitory factor suicide substrate inhibits motility and growth of lung cancer cells.. Cancer Res.

[35] Garai J, Lorand T (2009). Macrophage migration inhibitory factor (MIF) tautomerase inhibitors as potential novel anti-inflammatory agents: current developments.. Curr Med Chem.

[36] Jansa P, Grummt I (1999). Mechanism of transcription termination: PTRF interacts with the largest subunit of RNA polymerase I and dissociates paused transcription complexes from yeast and mouse.. Mol Gen Genet.

[37] Hill MM, Bastiani M, Luetterforst R, Kirkham M, Kirkham A (2008). PTRF-Cavin, a conserved cytoplasmic protein required for caveola formation and function.. Cell.

[38] Cohen AW, Hnasko R, Schubert W, Lisanti MP (2004). Role of caveolae and caveolins in health and disease.. Physiol Rev.

[39] Aboulaich N, Ortegren U, Vener AV, Stralfors P (2006). Association and insulin regulated translocation of hormone-sensitive lipase with PTRF.. Biochem Biophys Res Commun.

[40] Hayashi YK, Matsuda C, Ogawa M, Goto K, Tominaga K (2009). Human PTRF mutations cause secondary deficiency of caveolins resulting in muscular dystrophy with generalized lipodystrophy.. J Clin Invest.

[41] Aboulaich N, Vainonen JP, Stralfors P, Vener AV (2004). Vectorial proteomics reveal targeting, phosphorylation and specific fragmentation of polymerase I and transcript release factor (PTRF) at the surface of caveolae in human adipocytes.. Biochem J.

[42] Liu L, Pilch PF (2008). A critical role of cavin (polymerase I and transcript release factor) in caveolae formation and organization.. J Biol Chem.

[43] Bai L, Deng X, Li J, Wang M, Li Q (2011). Regulation of cellular senescence by the essential caveolar component PTRF/Cavin-1.. Cell Res.

[44] Volonte D, Galbiati F (2011). Polymerase I and transcript release factor (PTRF)/cavin-1 is a novel regulator of stress-induced premature senescence.. J Biol Chem.

[45] Zhu H, Lin P, De G, Choi KH, Takeshima H (2011). Polymerase transcriptase release factor (PTRF) anchors MG53 protein to cell injury site for initiation of membrane repair.. J Biol Chem.

[46] Gould ML, Williams G, Nicholson HD (2010). Changes in caveolae, caveolin, and polymerase 1 and transcript release factor (PTRF) expression in prostate cancer progression.. Prostate.

[47] Aung CS, Hill MM, Bastiani M, Parton RG, Parat MO (2010). PTRF-cavin-1 expression decreases the migration of PC3 prostate cancer cells: role of matrix metalloprotease 9.. Eur J Cell Biol.

[48] Shen J, Behrens C, Wistuba II, Feng L, Lee JJ (2006). Identification and validation of differences in protein levels in normal, premalignant, and malignant lung cells and tissues using high-throughput Western Array and immunohistochemistry.. Cancer Res.

[49] Bai L, Deng X, Li Q, An W, Deli A (2011). Down-regulation of the cavin family proteins in breast cancer.. J Cell Biochem.

[50] Guha U, Chaerkady R, Marimuthu A, Patterson AS, Kashyap MK (2008). Comparisons of tyrosine phosphorylated proteins in cells expressing lung cancer-specific alleles of EGFR and KRAS.. Proc Natl Acad Sci U S A.

