# Supplementary figures and images for: PTRF/Cavin-1 and MIF Proteins Are Identified as Non-Small Cell Lung Cancer Biomarkers by Label-Free Proteomics
Source: PLoS One. 2012 Mar 26;7(3):e33752. doi: 10.1371/journal.pone.0033752 (PMC3312891; doi:10.1371/journal.pone.0033752)

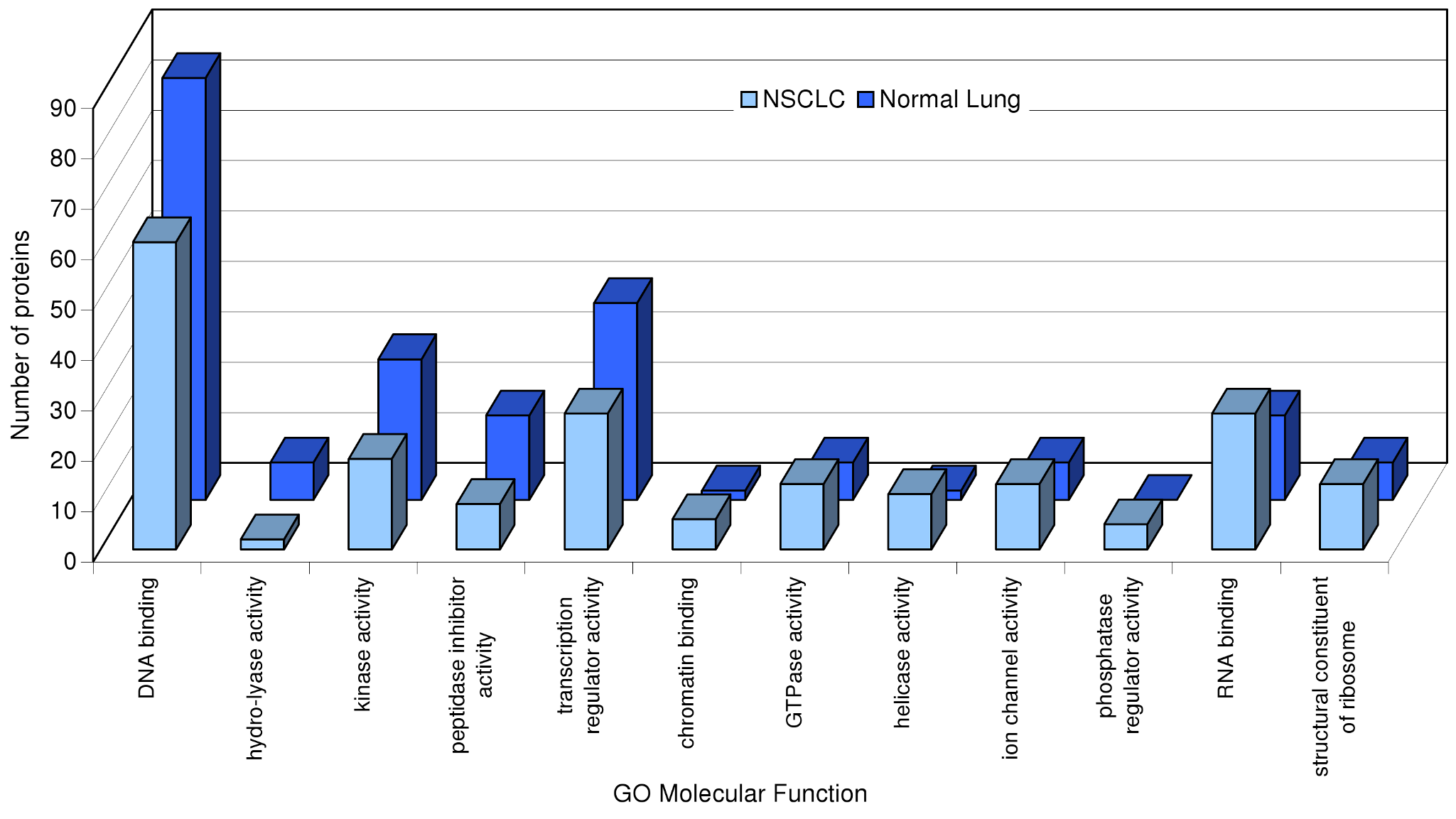

Supplement: Figure S1 — Analysis of differences in GO Molecular Function between NSCLC and normal lung. Comparison of number of proteins assigned to each GO pathway category. Normal tissue sample categories are represented as fold-change in relation to this category. Statistical significance is tested using the binomial test. Only significant categories (p<0.05) are shown. (TIF) [file pone.0033752.s001.tif]

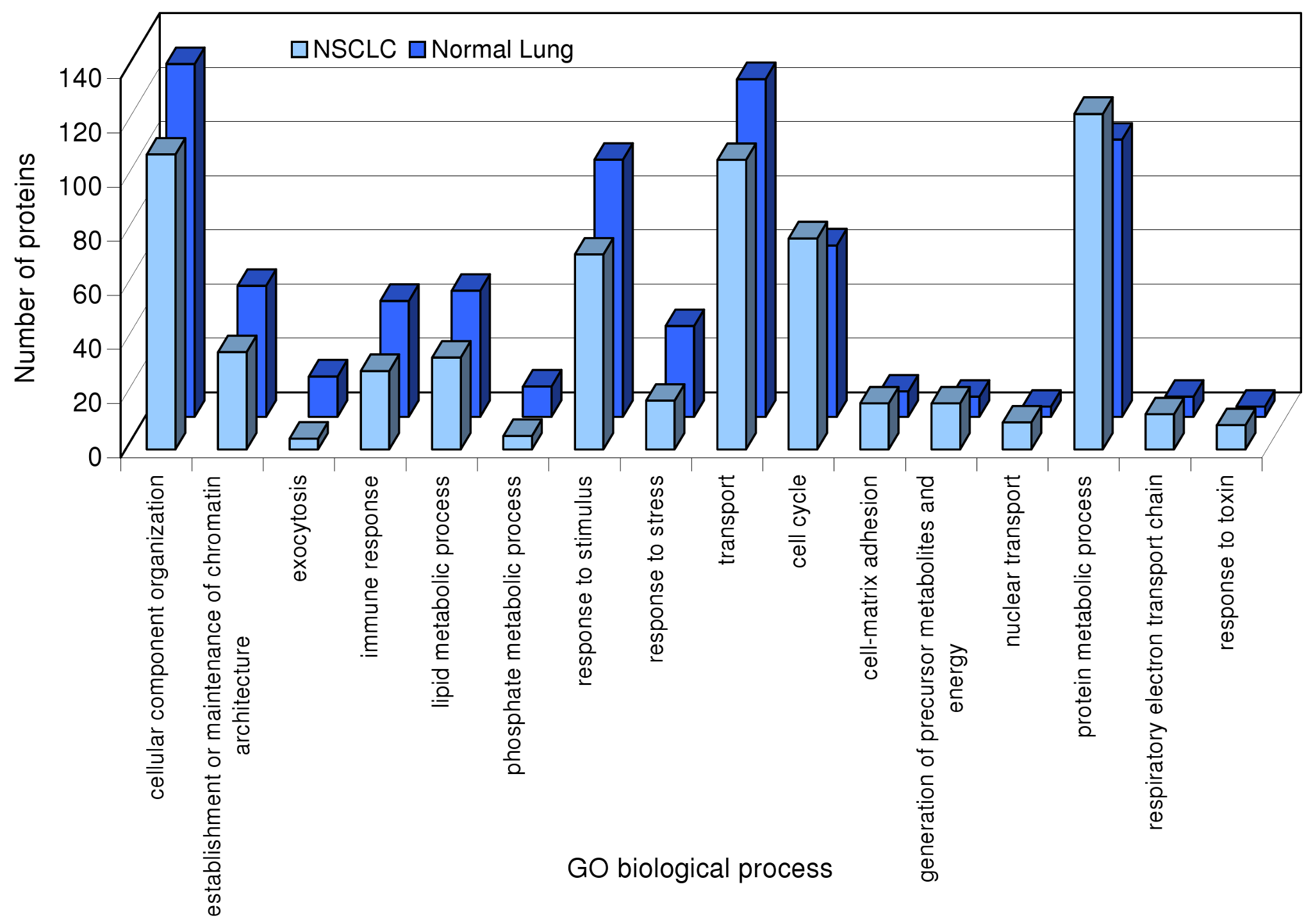

Supplement: Figure S2 — Analysis of differences in GO Biological Process between NSCLC and normal lung. Comparison of number of proteins assigned to each GO pathway category. Normal tissue sample categories are represented as fold-change in relation to this category. Statistical significance is tested using the binomial test. Only significant categories (p<0.05) are shown. (TIF) [file pone.0033752.s002.tif]

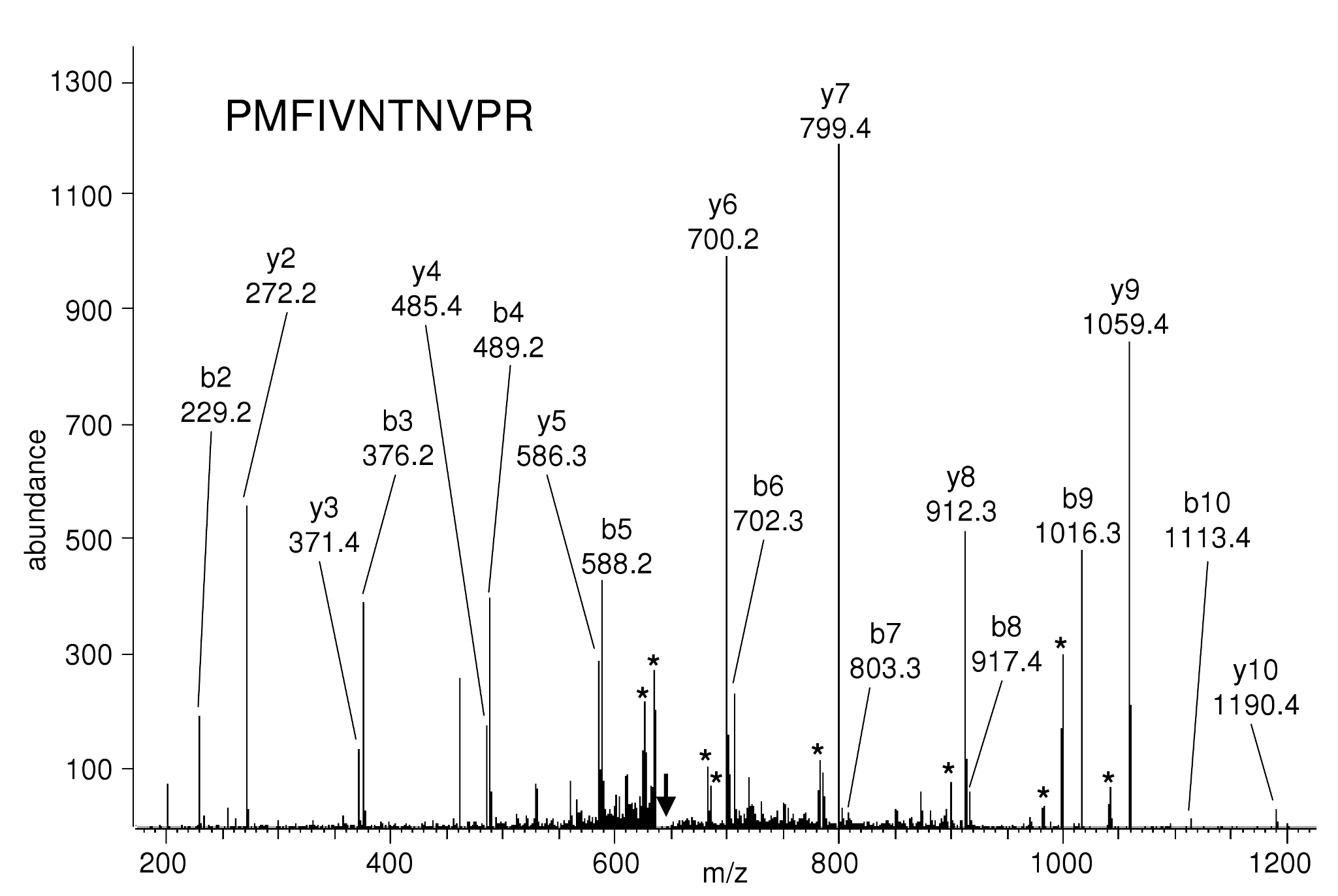

Supplement: Figure S3 — Fragmentation spectra from PTRF SLKESEALPEK tryptic peptide. Diagram shows fragment ions corresponding to main fragmentation series (b-amino and y-carboxy). * indicates water loss; 2+, doubly charged fragment. Parental ion is marked with an arrow. (TIF) [file pone.0033752.s003.tif]

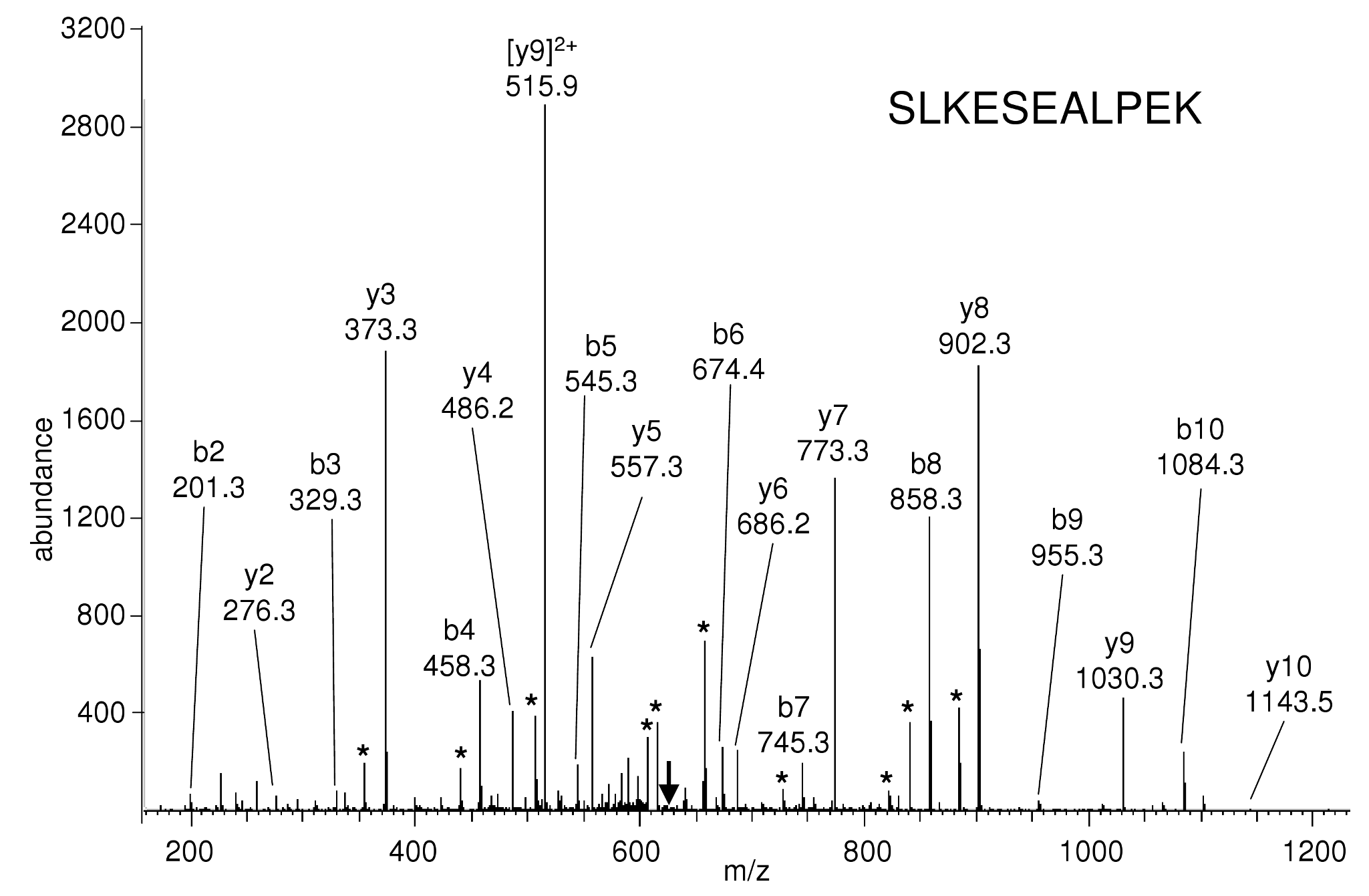

Supplement: Figure S4 — Fragmentation spectra from MIF PMFIVNTNVPR tryptic peptide. Diagram shows fragment ions corresponding to main fragmentation series (b-amino and y-carboxy). * indicates water loss. Parental ion is marked with an arrow. (TIF) [file pone.0033752.s004.tif]
