# Supplementary material for: PTRF/Cavin-1 and MIF Proteins Are Identified as Non-Small Cell Lung Cancer Biomarkers by Label-Free Proteomics
Source: PLoS One. 2012 Mar 26;7(3):e33752. doi: 10.1371/journal.pone.0033752 (PMC3312891; doi:10.1371/journal.pone.0033752)
Supplement: Table S1 — Gene Ontology analyses performed with PANTHER. Normal lung protein list was used as reference list. (PDF) [file pone.0033752.s005.pdf]

Molecular Function	Normal(268)	Tumor(499)	Tumor(expected)	Tumor(over/under)	Tumor(P-value)
acyltransferase activity	3	6	5,59	+	4,86E-01
adenylate cyclase activity	1	0	1,86	-	1,55E-01
amino acid transmembrane transporte	3	2	5,59	-	8,20E-02
aminoacyl-tRNA ligase activity	1	2	1,86	+	5,56E-01
anion channel activity	1	4	1,86	+	1,19E-01
antioxidant activity	3	4	5,59	-	3,43E-01
ATPase activity, coupled to transmeml	1	1	1,86	-	4,44E-01
binding	143	259	266,26	-	2,72E-01
calcium ion binding	20	31	37,24	-	1,64E-01
calcium-dependent phospholipid bindir	7	11	13,03	-	3,47E-01
calmodulin binding	7	17	13,03	+	1,64E-01
carbohydrate transmembrane transpor	2	4	3,72	+	5,11E-01
carboxy-lyase activity	0	2	0	+	0,00E+00
catalytic activity	88	184	163,85	+	3,14E-02
cation channel activity	2	3	3,72	-	4,89E-01
cation transmembrane transporter acti	5	5	9,31	-	9,60E-02
chromatin binding	1	6	1,86	+	1,19E-02
cyclic nucleotide-gated ion channel ac	1	1	1,86	-	4,44E-01
cytokine receptor activity	2	3	3,72	-	4,89E-01
cytoskeletal protein binding	11	26	20,48	+	1,30E-01
deacetylase activity	0	1	0	+	0,00E+00
deaminase activity	0	1	0	+	0,00E+00
DNA binding	45	61	83,79	-	2,86E-03
DNA helicase activity	1	6	1,86	+	1,19E-02
DNA photolyase activity	0	1	0	+	0,00E+00
DNA polymerase processivity factor ac	0	1	0	+	0,00E+00
DNA-directed DNA polymerase activity	2	3	3,72	-	4,89E-01
DNA-directed RNA polymerase activity	0	1	0	+	0,00E+00
enzyme activator activity	2	3	3,72	-	4,89E-01
enzyme inhibitor activity	10	14	18,62	-	1,65E-01
enzyme regulator activity	27	50	50,27	-	5,21E-01
extracellular matrix structural constitue	5	8	9,31	-	4,14E-01
gap junction channel activity	0	2	0	+	0,00E+00
G-protein coupled receptor activity	1	2	1,86	+	5,56E-01
GTPase activity	4	13	7,45	+	3,96E-02
guanylate cyclase activity	1	0	1,86	-	1,55E-01
guanyl-nucleotide exchange factor acti	1	2	1,86	+	5,56E-01
helicase activity	1	11	1,86	+	3,97E-06
hydrogen ion transmembrane transpor	2	1	3,72	-	1,13E-01
hydrolase activity	32	63	59,58	+	3,38E-01
hydrolase activity, acting on ester bonc	12	16	22,34	-	9,86E-02
hydrolase activity, hydrolyzing N-glyco:	2	1	3,72	-	1,13E-01
hydrolase activity, hydrolyzing O-glyco	0	2	0	+	0,00E+00
hydro-lyase activity	4	2	7,45	-	2,04E-02
intramolecular transferase activity	1	1	1,86	-	4,44E-01
ion channel activity	4	13	7,45	+	3,96E-02
isomerase activity	6	17	11,17	+	6,03E-02
kinase activator activity	2	3	3,72	-	4,89E-01
kinase activity	15	18	27,93	-	2,75E-02
kinase inhibitor activity	1	1	1,86	-	4,44E-01
kinase regulator activity	5	9	9,31	-	5,46E-01
ligand-dependent nuclear receptor acti	1	1	1,86	-	4,44E-01
ligand-gated ion channel activity	2	2	3,72	-	2,80E-01
ligase activity	6	9	11,17	-	3,20E-01
lipid binding	0	1	0	+	0,00E+00
lipid transporter activity	2	5	3,72	+	3,17E-01
lyase activity	7	10	13,03	-	2,45E-01
methyltransferase activity	3	2	5,59	-	8,20E-02
microtubule motor activity	2	5	3,72	+	3,17E-01
motor activity	3	9	5,59	+	1,12E-01
nucleic acid binding	70	122	130,34	-	2,13E-01
nucleotidyltransferase activity	2	6	3,72	+	1,73E-01
oxidoreductase activity	23	48	42,82	+	2,24E-01
peptidase activity	9	23	16,76	+	8,16E-02
peptidase inhibitor activity	9	9	16,76	-	2,76E-02
peroxidase activity	3	4	5,59	-	3,43E-01
phosphatase activity	0	5	0	+	0,00E+00

phosphatase inhibitor activity	0	4	0	+	0,00E+00
phosphatase regulator activity	0	5	0	+	0,00E+00
protein binding	73	143	135,92	+	2,53E-01
protein disulfide isomerase activity	1	4	1,86	+	1,19E-01
racemase and epimerase activity	1	3	1,86	+	2,86E-01
receptor activity	23	42	42,82	-	4,88E-01
receptor binding	38	62	70,75	-	1,44E-01
RNA binding	9	27	16,76	+	1,15E-02
RNA helicase activity	0	7	0	+	0,00E+00
RNA splicing factor activity, transesteri	5	14	9,31	+	8,85E-02
small GTPase regulator activity	12	21	22,34	-	4,40E-01
SNAP receptor activity	0	1	0	+	0,00E+00
structural constituent of cytoskeleton	39	80	72,62	+	1,90E-01
structural constituent of myelin sheath	0	1	0	+	0,00E+00
structural constituent of ribosome	4	13	7,45	+	3,96E-02
structural molecule activity	51	105	94,96	+	1,39E-01
transaldolase activity	1	1	1,86	-	4,44E-01
transaminase activity	0	1	0	+	0,00E+00
transcription cofactor activity	6	7	11,17	-	1,30E-01
transcription factor activity	21	27	39,1	-	2,23E-02
transcription regulator activity	21	27	39,1	-	2,23E-02
transferase activity	26	45	48,41	-	3,36E-01
transferase activity, transferring glycos	1	1	1,86	-	4,44E-01
transketolase activity	0	1	0	+	0,00E+00
translation elongation factor activity	3	8	5,59	+	2,00E-01
translation factor activity, nucleic acid I	6	14	11,17	+	2,33E-01
translation initiation factor activity	4	9	7,45	+	3,30E-01
translation regulator activity	6	14	11,17	+	2,33E-01
translation release factor activity	0	2	0	+	0,00E+00
transmembrane receptor protein kinas	3	3	5,59	-	1,91E-01
transmembrane receptor protein serine	1	0	1,86	-	1,55E-01
transmembrane receptor protein tyrosi	3	3	5,59	-	1,91E-01
transmembrane transporter activity	13	21	24,21	-	2,94E-01
transporter activity	14	22	26,07	-	2,41E-01
tumor necrosis factor receptor activity	1	1	1,86	-	4,44E-01
ubiquitin-protein ligase activity	3	5	5,59	-	5,14E-01
Unclassified	75	130	139,65	-	1,81E-01
voltage-gated ion channel activity	2	4	3,72	+	5,11E-01
voltage-gated potassium channel activ	2	3	3,72	-	4,89E-01

**Biological Process**	**Normal(268)**	**Tumor(499)**	**Tumor(expected)**	**Tumor(over/under)**	**Tumor(P-value)**
acyl-CoA metabolic process	0	1	0	+	0,00E+00
amino acid transport	1	0	1,86	-	1,55E-01
anatomical structure morphogenesis	43	73	80,06	-	2,13E-01
angiogenesis	12	17	22,34	-	1,46E-01
anion transport	0	5	0	+	0,00E+00
antigen processing and presentation	4	5	7,45	-	2,45E-01
antigen processing and presentation o	1	2	1,86	+	5,56E-01
apoptosis	12	26	22,34	+	2,42E-01
B cell mediated immunity	6	9	11,17	-	3,20E-01
blood circulation	14	15	26,07	-	1,19E-02
blood coagulation	7	13	13,03	-	5,69E-01
carbohydrate metabolic process	25	44	46,55	-	3,83E-01
carbohydrate transport	9	9	16,76	-	2,76E-02
cation transport	7	9	13,03	-	1,60E-01
cell adhesion	22	47	40,96	+	1,82E-01
cell communication	78	138	145,23	-	2,55E-01
cell cycle	34	78	63,31	+	3,08E-02

cell motion	25	44	46,55 -	3,83E-01
cell surface receptor linked signal trans	22	33	40,96 -	1,09E-01
cell-cell adhesion	19	31	35,38 -	2,54E-01
cell-cell signaling	23	34	42,82 -	8,85E-02
cell-matrix adhesion	5	17	9,31 +	1,41E-02
cellular amino acid and derivative meta	7	12	13,03 -	4,58E-01
cellular component morphogenesis	43	73	80,06 -	2,13E-01
cellular component organization	70	109	130,34 -	1,56E-02
cellular defense response	9	10	16,76 -	5,21E-02
cellular glucose homeostasis	0	4	0 +	0,00E+00
cellular process	137	255	255,09 -	5,15E-01
chromosome segregation	8	17	14,9 +	3,24E-01
coenzyme metabolic process	0	1	0 +	0,00E+00
complement activation	9	10	16,76 -	5,21E-02
cytokinesis	8	15	14,9 +	5,25E-01
defense response to bacterium	8	11	14,9 -	1,88E-01
developmental process	62	118	115,44 +	4,10E-01
ectoderm development	32	59	59,58 -	5,02E-01
embryonic development	6	3	11,17 -	4,02E-03
endocytosis	15	25	27,93 -	3,26E-01
establishment or maintenance of chror	26	36	48,41 -	3,19E-02
exocytosis	8	4	14,9 -	8,18E-04
extracellular transport	3	5	5,59 -	5,14E-01
female gamete generation	5	4	9,31 -	4,40E-02
ferredoxin metabolic process	0	1	0 +	0,00E+00
fertilization	2	3	3,72 -	4,89E-01
gamete generation	11	11	20,48 -	1,52E-02
generation of precursor metabolites ar	4	17	7,45 +	1,67E-03
gut mesoderm development	0	1	0 +	0,00E+00
heart development	4	8	7,45 +	4,68E-01
hemopoiesis	2	2	3,72 -	2,80E-01
homeostatic process	3	8	5,59 +	2,00E-01
immune response	23	29	42,82 -	1,31E-02
immune system process	65	105	121,03 -	5,08E-02
induction of apoptosis	6	7	11,17 -	1,30E-01
intracellular protein transport	40	73	74,48 -	4,57E-01
intracellular signaling cascade	27	51	50,27 +	4,79E-01
ion transport	8	16	14,9 +	4,21E-01
lipid metabolic process	25	34	46,55 -	2,78E-02
lipid transport	3	7	5,59 +	3,27E-01
localization	0	2	0 +	0,00E+00
macrophage activation	12	17	22,34 -	1,46E-01
meiosis	5	7	9,31 -	2,87E-01
mesoderm development	26	47	48,41 -	4,53E-01
metabolic process	150	281	279,29 +	4,57E-01
mitochondrion organization	1	0	1,86 -	1,55E-01
mitosis	22	37	40,96 -	2,91E-01
muscle contraction	13	19	24,21 -	1,63E-01
muscle organ development	4	10	7,45 +	2,16E-01
natural killer cell activation	1	1	1,86 -	4,44E-01
negative regulation of apoptosis	5	7	9,31 -	2,87E-01
nervous system development	17	34	31,65 +	3,58E-01
neurological system process	23	44	42,82 +	4,48E-01
neuronal action potential propagation	1	2	1,86 +	5,56E-01
neurotransmitter secretion	5	6	9,31 -	1,77E-01
nitrogen compound metabolic process	1	4	1,86 +	1,19E-01
nuclear transport	2	10	3,72 +	4,88E-03
nucleobase, nucleoside, nucleotide an	58	110	107,99 +	4,31E-01
nucleobase, nucleoside, nucleotide an	2	2	3,72 -	2,80E-01
organelle organization	27	36	50,27 -	1,69E-02
oxidative phosphorylation	1	3	1,86 +	2,86E-01
oxygen and reactive oxygen species m	3	9	5,59 +	1,12E-01
pattern specification process	1	1	1,86 -	4,44E-01
peroxisomal transport	0	1	0 +	0,00E+00
phosphate metabolic process	6	5	11,17 -	3,24E-02
phosphate transport	3	3	5,59 -	1,91E-01
porphyrin metabolic process	0	2	0 +	0,00E+00
primary metabolic process	142	266	264,4 +	4,61E-01

protein metabolic process	55	124	102,41	+	1,09E-02
protein transport	40	73	74,48	-	4,57E-01
regulation of biological process	2	1	3,72	-	1,13E-01
regulation of liquid surface tension	3	4	5,59	-	3,43E-01
regulation of vasoconstriction	2	1	3,72	-	1,13E-01
reproduction	11	12	20,48	-	2,88E-02
respiratory electron transport chain	4	13	7,45	+	3,96E-02
response to external stimulus	7	13	13,03	-	5,69E-01
response to pheromone	2	2	3,72	-	2,80E-01
response to stimulus	51	72	94,96	-	4,20E-03
response to stress	18	18	33,51	-	1,92E-03
response to toxin	2	9	3,72	+	1,39E-02
RNA localization	0	2	0	+	0,00E+00
segment specification	1	1	1,86	-	4,44E-01
sensory perception	8	17	14,9	+	3,24E-01
sensory perception of sound	1	4	1,86	+	1,19E-01
signal transduction	71	132	132,2	-	5,15E-01
skeletal system development	10	19	18,62	+	4,97E-01
spermatogenesis	5	7	9,31	-	2,87E-01
sulfur metabolic process	2	6	3,72	+	1,73E-01
synaptic transmission	9	14	16,76	-	2,97E-01
system development	28	56	52,13	+	3,06E-01
system process	41	65	76,34	-	8,68E-02
transport	67	107	124,75	-	3,56E-02
tricarboxylic acid cycle	0	4	0	+	0,00E+00
Unclassified	56	92	104,27	-	9,62E-02
vesicle-mediated transport	26	42	48,41	-	1,87E-01
visual perception	5	9	9,31	-	5,46E-01
vitamin biosynthetic process	0	2	0	+	0,00E+00
vitamin metabolic process	0	2	0	+	0,00E+00
vitamin transport	5	7	9,31	-	2,87E-01

**Pathway**	**Normal(268)**	**Tumor(499)**	**Tumor(expected)**	**Tumor(over/under)**	**Tumor(P-value)**
5HT1 type receptor mediated signaling	0	1	0	+	0,00E+00
5HT2 type receptor mediated signaling	1	0	1,86	-	1,55E-01
5-Hydroxytryptamine biosynthesis	0	1	0	+	0,00E+00
5-Hydroxytryptamine degredation	0	1	0	+	0,00E+00
Adrenaline and noradrenaline biosynth	0	1	0	+	0,00E+00
Alpha adrenergic receptor signaling pa	0	1	0	+	0,00E+00
Alzheimer disease-amyloid secretase		1	4	1,86	+	1,19E-01
Alzheimer disease-presenilin pathway	4	5	7,45	-	2,45E-01
Angiogenesis	5	6	9,31	-	1,77E-01
Apoptosis signaling pathway	6	7	11,17	-	1,30E-01
ATP synthesis	1	1	1,86	-	4,44E-01
Axon guidance mediated by semaphor	0	2	0	+	0,00E+00
B cell activation	1	0	1,86	-	1,55E-01
Beta1 adrenergic receptor signaling pa	0	1	0	+	0,00E+00
Beta2 adrenergic receptor signaling pa	0	1	0	+	0,00E+00
Blood coagulation	5	8	9,31	-	4,14E-01
Cadherin signaling pathway	4	5	7,45	-	2,45E-01
Circadian clock system	1	2	1,86	+	5,56E-01
Cytoskeletal regulation by Rho GTPas	8	8	14,9	-	3,74E-02
De novo purine biosynthesis	1	2	1,86	+	5,56E-01
De novo pyrimidine deoxyribonucleotic	0	1	0	+	0,00E+00
De novo pyrmidine ribonucleotides bio	0	1	0	+	0,00E+00
DNA replication	1	3	1,86	+	2,86E-01
EGF receptor signaling pathway	2	8	3,72	+	3,58E-02
Endothelin signaling pathway	1	4	1,86	+	1,19E-01
FAS signaling pathway	2	4	3,72	+	5,11E-01
FGF signaling pathway	3	9	5,59	+	1,12E-01

Fructose galactose metabolism	1	2	1,86	+	5,56E-01
GABA-B_receptor_II_signaling	0	2	0	+	0,00E+00
Gamma-aminobutyric acid synthesis	0	1	0	+	0,00E+00
General transcription by RNA polymera	2	2	3,72	-	2,80E-01
General transcription regulation	1	0	1,86	-	1,55E-01
Glutamine glutamate conversion	1	1	1,86	-	4,44E-01
Glycolysis	4	12	7,45	+	7,47E-02
Hedgehog signaling pathway	0	2	0	+	0,00E+00
Heme biosynthesis	0	1	0	+	0,00E+00
Heterotrimeric G-protein signaling path	4	5	7,45	-	2,45E-01
Heterotrimeric G-protein signaling path	0	1	0	+	0,00E+00
Histamine H1 receptor mediated signa	1	0	1,86	-	1,55E-01
Histamine H2 receptor mediated signa	0	1	0	+	0,00E+00
Huntington disease	7	9	13,03	-	1,60E-01
Inflammation mediated by chemokine a	14	9	26,07	-	7,97E-05
Insulin/IGF pathway-mitogen activated	1	2	1,86	+	5,56E-01
Insulin/IGF pathway-protein kinase B s	0	1	0	+	0,00E+00
Integrin signalling pathway	9	7	16,76	-	5,69E-03
Interferon-gamma signaling pathway	1	1	1,86	-	4,44E-01
Interleukin signaling pathway	2	1	3,72	-	1,13E-01
Lysine biosynthesis	1	0	1,86	-	1,55E-01
Mannose metabolism	1	0	1,86	-	1,55E-01
Metabotropic glutamate receptor group	0	1	0	+	0,00E+00
Metabotropic glutamate receptor group	0	1	0	+	0,00E+00
Muscarinic acetylcholine receptor 1 an	1	2	1,86	+	5,56E-01
Muscarinic acetylcholine receptor 2 an	1	3	1,86	+	2,86E-01
Nicotinic acetylcholine receptor signali	5	7	9,31	-	2,87E-01
Notch signaling pathway	0	1	0	+	0,00E+00
Oxidative stress response	0	1	0	+	0,00E+00
Oxytocin receptor mediated signaling p	1	0	1,86	-	1,55E-01
p53 pathway	2	9	3,72	+	1,39E-02
p53 pathway by glucose deprivation	0	1	0	+	0,00E+00
p53 pathway feedback loops 2	1	1	1,86	-	4,44E-01
Parkinson disease	6	12	11,17	+	4,41E-01
PDGF signaling pathway	1	3	1,86	+	2,86E-01
Pentose phosphate pathway	3	4	5,59	-	3,43E-01
Phenylethylamine degradation	1	1	1,86	-	4,44E-01
PI3 kinase pathway	0	6	0	+	0,00E+00
Plasminogen activating cascade	3	3	5,59	-	1,91E-01
Pyrimidine Metabolism	0	1	0	+	0,00E+00
Pyruvate metabolism	1	3	1,86	+	2,86E-01
Ras Pathway	1	1	1,86	-	4,44E-01
T cell activation	1	0	1,86	-	1,55E-01
TCA cycle	0	1	0	+	0,00E+00
TGF-beta signaling pathway	1	3	1,86	+	2,86E-01
Threonine biosynthesis	1	0	1,86	-	1,55E-01
Thyrotropin-releasing hormone recepto	1	0	1,86	-	1,55E-01
Toll receptor signaling pathway	1	1	1,86	-	4,44E-01
Transcription regulation by bZIP transc	2	1	3,72	-	1,13E-01
Triacylglycerol metabolism	1	0	1,86	-	1,55E-01
Tryptophan biosynthesis	0	1	0	+	0,00E+00
Ubiquitin proteasome pathway	0	2	0	+	0,00E+00
Unclassified	223	425	415,21	+	1,32E-01
VEGF signaling pathway	3	3	5,59	-	1,91E-01
Vitamin D metabolism and pathway	1	1	1,86	-	4,44E-01
Wnt signaling pathway	6	5	11,17	-	3,24E-02
