# Supplementary material for: PTRF/Cavin-1 and MIF Proteins Are Identified as Non-Small Cell Lung Cancer Biomarkers by Label-Free Proteomics
Source: PLoS One. 2012 Mar 26;7(3):e33752. doi: 10.1371/journal.pone.0033752 (PMC3312891; doi:10.1371/journal.pone.0033752)
Supplement: Table S2 — SIEVE label-free quantification. Data obtained from SIEVE analyses, including relative expression values. (PDF) [file pone.0033752.s006.pdf]

ID	MZ	Time	MS2Corr	MS2	Ratio_AvsB	StdDev_AvsB	PValue_AvsB	TICNorm Ratio	TICNorm StdDev	TICNorm PValue	GoodID	II_LUNG_9	II_LUNG_12	II_LUNG_15	II_LUNG_30	II_LUNG_33	II_LUNG_36	II_LUNG_37	II_LUNG_43	II_LUNG_45	II_LUNG_53	II_LUNG_54	II_LUNG_57	II_LUNG_59	II_LUNG_3	II_LUNG_7	AvgCharge
1	459,479	39,082	0,901	69	0,38	0,289	1,20E-04	0,404	0,253	4,80E-06	0	8737634	37666982	30430958	14591473	34872927	2958982	22128957	8436638	39682320	49794545	54310555	53817736	64531606	22486116	17168805	3,9
2	459,22974	39,082	0,902	15	0,381	0,289	1,30E-04	0,405	0,253	4,90E-06	0	8652079	36811802	29656742	14300989	34196876	2946224	21612911	8312741	38727344	48701974	53124162	52667781	63235247	21996984	17033854	3,85
3	510,58398	26,034	0,576	94	0,294	0,249	2,50E-05	0,266	0,215	1,50E-05	1	4814141	22539994	15544029	6023955	16595868	1416668	7740696	3327652	24862331	31706131	34216388	38256608	37332234	11856204	7842106	3
4	637,86713	51,81	0,806	80	0,379	0,36	1,80E-03	0,442	0,325	1,00E-04	1	3345419	24911926	16703004	8889711	21795286	1314356	11870981	4368489	22371905	34255464	29591727	22761271	39564853	12086642	7180171	2,07
5	593,82764	40,18	0,776	69	1,883	3,698	2,90E-01	2,13	4,048	2,30E-01	0	158822	6713382	5012275	25723852	2101930	17556714	2833184	6703390	2202321	4067637	7673636	391967	3845617	962438	714059	2,08
7	652,36169	38,523	0,717	70	2,324	2,676	2,60E-02	2,435	2,862	1,70E-02	1	3885274	11125131	13922603	19044511	1517398	8849043	7460116	3196856	5507908	3050704	4210289	706606	4717439	10489789	5077139	2,99
9	652,02856	38,523	0,747	5	2,324	2,687	2,60E-02	2,434	2,875	1,70E-02	0	3514619	10283105	12854133	17683427	1424681	8191306	6855781	2911998	5106196	2823045	3868659	651371	4358755	9724033	4689828	3,01
12	600,30505	51,267	0,584	26	0,306	0,487	4,90E-02	0,366	0,529	2,70E-02	1	806395	10474878	19642732	3894240	10213595	122225	1798739	14497995	6692126	29322030	16769101	16795504	37954299	3192618	1185303	4,9
13	536,28076	47,988	0,782	89	0,372	0,324	2,10E-04	0,401	0,315	7,20E-05	1	2508809	17060178	12704057	7192974	18120811	1089643	8096964	2999047	20576051	24426628	22224170	19611280	30525280	11574841	5867464	2,08
14	660,35657	33,438	0,908	2	0,874	1,509	7,70E-01	0,95	1,599	9,10E-01	0	173142	7772818	6054463	15981766	1785668	11755766	3031587	2483362	3718015	7197476	12172852	783977	5725069	1813909	874941	1,38
15	506,58334	33,234	0,619	64	2,362	3,349	6,10E-02	2,359	3,122	4,10E-02	0	2034933	3664602	3340613	17084234	23819571	6458176	7723530	11408303	5083574	999528	2627983	2890290	6806260	7650103	3762958	2,86
17	547,31873	34,143	0,711	58	0,649	0,558	1,20E-01	0,684	0,534	1,10E-01	0	1324323	14573003	14110057	11535925	5960493	6436852	9041844	8906699	12951932	7066429	15128881	11631108	21125400	11444431	4864033	3
18	600,50385	57,173	0,746	16	0,486	0,686	1,60E-01	0,525	0,661	1,40E-01	1	936268	15444928	14931600	8789387	13659496	1026211	19391727	2522042	10383867	25877668	28130639	24128665	1373285	6366895	4247054	4,99
19	418,22104	30,283	0,711	25	1,102	1,693	8,10E-01	1,138	1,7	7,50E-01	0	391573	5288198	5310893	13921748	2253593	7478734	2900234	5914027	1901411	5342586	7065225	965494	5204107	1017306	644352	1,74
20	557,30212	49,744	0,848	95	0,343	0,329	7,10E-03	0,382	0,299	1,20E-03	1	2052012	12484955	11286689	5767194	12827366	795633	8429475	3853548	12913797	15858086	25354462	17144809	28080878	6342628	4333177	3,04
21	657,83746	34,087	0,77	77	0,332	0,307	3,20E-05	0,344	0,271	1,10E-05	1	1150339	12500872	7734454	3845628	8071799	337220	5110567	1321069	11642294	14707273	15526380	14281466	17786637	6191059	2878969	2
22	489,95297	41,664	0,719	39	0,573	0,904	2,70E-01	0,627	0,927	3,10E-01	0	529704	14486429	7495935	5865743	1065086	1581888	10560381	7416230	2086764	17138477	14347040	3412588	9709165	3132495	1373492	2,97
23	547,65222	34,143	0	1	0,648	0,56	1,20E-01	0,682	0,537	1,10E-01	0	1108562	12841348	12421923	10112845	5215053	5670966	7945037	7766009	11396170	6228245	13324334	10198055	18685597	10136706	4296708	3,02
24	557,63562	49,744	0,958	15	0,341	0,332	7,30E-03	0,379	0,304	1,20E-03	1	1736209	11362914	10205874	5135711	11632255	622890	7346434	3406176	11546669	14246054	22973933	15489127	25333971	5686145	3876506	2,99
26	652,69501	38,523	0,652	2	2,188	2,704	4,30E-02	2,321	2,794	2,50E-02	0	1957905	7188864	8252293	11169107	766378	5555018	4227511	1610160	3071684	1663748	2494792	399394	3685638	6018288	2775623	2,98
28	483,9375	27,916	0,872	69	0,318	0,28	2,00E-03	0,301	0,237	4,60E-04	0	1870740	11113989	7814394	3342159	6506157	1057129	8549843	3335453	9355398	17314535	21485982	17484528	16957585	5614005	3254633	2,97
30	391,22794	20,088	0,776	44	0,193	0,215	4,60E-03	0,172	0,19	3,50E-03	0	1181217	3961086	6752227	2189953	3863123	649197	883013	1007468	10608633	11802897	9268179	20274998	17628050	1507103	4860255	2,26
32	400,7713	26,574	0,955	31	1,852	2,366	9,50E-02	1,826	2,222	9,30E-02	0	2762943	6881465	9945714	10230612	1426117	4453273	3621556	1552773	2370499	1719996	2814570	948979	5292829	4648281	3163684	1,64
33	416,25076	32,203	0,917	58	2,622	2,206	1,60E-03	2,71	1,836	2,30E-04	1	4249406	9288187	9261502	10747110	7325054	3959789	14939828	7894065	2409427	2806975	4564237	1478373	4075800	9001876	3737980	1,68
36	600,30542	57,814	0,6	69	0,408	0,645	1,20E-01	0,413	0,516	8,60E-02	1	1105786	17060632	3026351	9737357	14885718	1191487	21817234	636908	11272474	29505471	31354556	26532756	1140721	7183057	4842494	4,96
37	409,5408	29,3	0,863	29	0,545	0,499	3,90E-02	0,528	0,431	1,40E-02	0	616837	8240303	7959518	9673390	6146413	2505267	7790367	5980874	8059307	11275082	14044271	5210056	11049885	3192609	2034098	2,59
40	796,9118	52,58	0,781	46	1,712	2,279	1,60E-01	2,052	2,628	7,30E-02	0	440285	3150622	8340909	8294259	1415065	4346209	6291193	4303273	2043725	1856848	3034843	480733	4101728	2193809	667478	3,92
41	484,27188	27,873	0,673	6	0,314	0,278	1,80E-03	0,296	0,235	3,80E-04	0	1505005	9254437	6385805	2716283	5327435	852549	6868250	2700584	7879835	14149336	17831594	14487865	13959077	4632346	2626449	2,96
45	559,93951	42,365	0,822	37	1,321	2,435	5,80E-01	1,531	2,749	4,10E-01	0	104017	3159551	2131701	8978125	1042445	6397129	937417	2340662	1067199	1983590	3893627	248374	2614368	455187	373885	3
46	612,30444	39,082	0,782	61	0,334	0,3	3,10E-04	0,352	0,27	1,40E-05	1	1884525	9445450	6463782	2799301	9810660	615841	5056371	1452948	10604664	11607443	17735315	14198136	15286536	5341799	3539871	2,88
47	797,16156	52,58	0,862	12	1,688	2,252	1,70E-01	2,005	2,616	8,30E-02	0	381221	2921267	7793524	7703202	1287293	3939995	5819270	3962681	2093145	1721308	2772840	400608	3823058	2083014	610768	3,98
48	611,97052	39,082	0,598	17	0,329	0,3	3,00E-04	0,346	0,271	1,50E-05	1	1710574	9034771	6197130	2612589	9417383	532437	4736672	1330147	10212661	11120353	17064222	13763249	14741936	5075556	3313661	3
49	684,29401	21,013	0,791	39	3,223	3,416	1,70E-03	2,988	2,915	2,10E-03	0	853342	2924690	5599442	2750177	2159478	5033059	3539026	2262091	238099	1082220	901531	685494	1658270	2001471	2311576	4,66
50	560,27386	42,409	0,795	14	1,279	2,298	6,20E-01	1,481	2,584	4,40E-01	0	152783	3119092	2051955	8548977	968675	6120459	866594	2244248	1074193	2013737	3829881	351808	2489568	463532	424190	3,21
52	544,9978	19,822	0,804	67	1,648	4,546	5,70E-01	1,331	3,358	7,00E-01	0	110879	1280492	4447473	2173144	42291100	122905	706491	8455166	1377973	2846808	5066809	1774798	8297264	4138051	105229	3,98
53	575,31213	36,15	0,75	60	0,792	0,834	4,20E-01	0,804	0,773	4,10E-01	0	794624	5340423	6327902	8051816	3226029	1934444	5496273	5560525	5012177	5018153	8094895	2072731	4896708	1943333	1097116	2,24
54	684,49371	21,013	0,865	20	3,321	3,575	1,60E-03	3,082	3,063	2,00E-03	0	756462	2584665	4987630	2430159	1941668	4497772	3166119	1955659	188546	936943	781428	588529	1440743	1786071	2039368	4,74
55	625,92413	55,337	0,755	53	0,385	0,512	5,60E-02	0,404	0,481	4,50E-02	0	343768	8059224	6925696	3269985	7038931	302585	8195915	1520546	4402723	12925469	15072035	14875523	4565544	2694559	1535348	5
56	544,74774	19,822	0,86	9	1,648	4,531	5,70E-01	1,329	3,342	7,00E-01	0	105155	1135301	3821198	1940913	37388571	108020	617006	7441197	1255111	2522006	4472783	1591069	7250096	3655054	109243	3,96
57	583,29565	56,417	0,963	22	4,778	15,486	3,90E-01	5,165	16,849	3,80E-01	0	7268143	221094	86333	83237	113759	76585	91525	97996	152568	120350	250571	260134	73897	63407	92951	3,48
60	626,12433	55,275	0,759	30	0,38	0,498	4,80E-02	0,398	0,464	3,70E-02	0	297066	7446896	6349358	2966412	6460867	268120	7486624	1525110	4169931	11773717	13989123	13673972	4635405	2478552	1390158	4,99
64	634,00433	55,275	0,787	7	1,1	1,621	8,10E-01	1,222	1,455	5,30E-01	0	232715	6968227	4487928	3394559	4816090	682553	3799994	3081790	1566058	3917011	5036995	771331	1765748	860309	390356	3,06
65	419,75577	26,649	0,408	19	1,883	3,587	2,80E-01	2,025	4,016	2,80E-01	0	88025	3140996	1539257	6302050	625797	4374443	729898	1201311	731519	2056960	3022954	273783	1934688	392123	11807518	1,66
67	581,3136	33,438	0,673	62	1,927	2,462	9,20E-02	2,066	2,534	6,00E-02	0	683593	2960508	4587479	5949618	445811	3521310	2259393	753305	1531798	838626	1533426	364156	2190303	2536489	1193033	2,22
68	480,78513	35,313	0,855	40	0,555	0,56	7,20E-02	0,575	0,509	4,30E-02	0	277461	5784424	5719009	4806346	2465987	1663414	4537618	4163416	4350521	6210692	9045415	2892429	6899854	2059455	1175214	2,28
69	661,57495	56,796	0	1	17,119	69,381	3,50E-01	20,717	79,738	3,50E-01	0	6217634	30122	14935	16336	106005	6309	30527	15711	12397	105941	27848	22388	20066	13325	7725	5,1
70	513,30908	32,923	0,688	5	1,975	7,379	5,20E-01	2,251	8,661	4,70E-01	0	5299073	338233	252849	415866	346381	299247	340391	180039	121425	1654763	106087	42123	87141	322008	151311	2,17
72	661,7746	56,626	0	1	17,985	74,92	3,50E-01	22,603	90,479	3,50E-01	0	5931330	34824	13039	17082	91222	6381	32066	15575	8461	101651	29924	8348	22862	12752	5484	5
73	489,6062	48,028	0,758	58	2,68	3,175	8,90E-03	3,023	2,86	6,70E-04	0	2324186	5810025	4420931	5214931	4791670	2444118	12161771	5531863	899773	1637050	3519408	709317	2697892	5045172	2987185	2,98
74	477,30518	42,498	0,641	56	2,218	1,758	2,20E-03	2,446	1,547	1,20E-04	1	3290980	5355435	4898583	6747329	7973018	3677336	9759090	5145532	2226682	1993155	4212669	1382639	3144842	7041749	3600102	1,74
75	521,77142	55,321	0,672	68	0,395	0,513	4,80E-02	0,417	0,488	4,00E-02	0	400193	7163272	6165878	2743731	5426996	252558	7290073	1525101	3440496	11193748	11818932	12443254	4719449	2179915	1342654	5,91
76	575,34167	25,855	0,809	72	0,288	0,299	2,80E-03	0,255	0,251	2,30E-03	1	538441	4547741	3703474	1421555	4219150	174913	2348991	808255	5280471	4759310	8117949	9263756	9209696	2082699	1242609	2
77	407,74319	23,379	0,966	8	5,992	12,957	2,10E-02	6,762	14,629	3,30E-02	1	5485519	585990	3193714	203313	212236	2061427	1343561	602213	148885	90711	222492	94014	974518	3283937	1370582	1,85
78	505,92184	30,484	0,724	20	2,498	2,843	2,40E-02	2,636	2,744	1,50E-02	1	2439455	4902438	3386282	3966402	753025	1263065	1159181	317609	831475	720673	1474914	375056	1062171	2825780	1291053	2,97
79	508,31076	42,993	0,796	46	1,046	1,433	9,00E-01	1,163	1,429	6,50E-01	0	91478	1954542	5616535	3044480	3293148	2085421	2876641	8168194	854145	4078566	2264822	2178235	4558887	1418877	592558	2,04
80	1030,47766	56,524	0,819	74	0,444	0,684	1,80E-01	0,49	0,647	1,50E-01	1	275837	4473300	3324140	1688775	3787296	274130	3130064	252615	3940542	8859350	7472432	2400538	345727	2285693	961711	2,01
82	737,12671	51,164	0,784	52	14,022	19,287	6,50E-04	18,647	25,174	9,00E-04	0	537839	5263813	3857238	1955955	1622548	1473828	5725242	2051664	77388	249878	480589	43092	242359	3534864	4638195	4
83	689,31232	43,627	0,831	22	1,664	3,767	4,20E-01	1,93	4,209	2,90E-01	0	56891	1109189	2066969	5489155	1658520	68066	609546	548252	167465	371716	1429868	90013	2096376	2148120	74502	4,7
84	633,67047	55,275	0,918	2	1,091	1,62	8,30E-01	1,212	1,449	5,50E-01	0	208605	6110655	3890862	2947263	4252768	602077	3307680	2649034	1384542	3419460	4477954	676233	1521175	735321	350956	3
85	395,24103	26,746	0,815	36	0,736	0,621	1,90E-01	0,67	0,511	6,10E-02	0	741643	4334491	4373582	5992604	3402494	1833729	4972261	3982581	4316647	3438132	5544092	2850595	6357768	2294495	1214943	2,29
86	570,41296	20,996	0,85	22	3,121	3,183	1,30E-03	2,895	2,677	1,50E-03	0	809156	2392519	4520591	2449332	1644875	4089660	3122084	1954972	246865	909409	771582	587187	1464486	1878588	1977857	6
87	895,95007	50,175	0,732	56	2,235	3,201	6,50E-02	2,489	4,188	6,90E-02	1	512915	2005044	4463239	3807687	282124	4263037	2445624	1064284	1727603	496011	824888	205940	1454998	1586579	624929	2
90	797,41138	52,58	0,75	3	1,679	2,2	1,70E-01	1,96	2,625	9,60E-02	0	280558	1734751	5085739	4949925	721642	2614517	3654804	2502389	1592651	1005479	1656407	319848	2353417	1224015	491266	3,97
91	583,04559	56,417	0,99	2	5,976	20,599	3,90E-01	6,482	22,442	3,90E-01	0	5756157	142702	33717	31944	63732	14250	36698	47137	93239	74365	164519	148908	34961	15770	25148	4,03
92	570,57965	20,996	0,929	9	3,101	3,15	1,40E-03	2,875	2,655	1,60E-03	0	701236	2126934	4050695	2188413	1451870	3653970	2783273	1731853	224801	837119	701830	515158	1297798	1711302	1783922	5,98
95	521,93768	55,3	0,76	28	0,393	0,514	4,80E-02	0,413	0,485	3,90E-02	0	324589	6739686	5579681	2469116	4931093	221501	6725817	1378998	3213411	10206890	11088701	11464778	4288622	1991082	1247469	5,94
98	566,76849	29,164	0,648	34	1,71	2,42	1,90E-01	1,679	2,454	2,10E-01	0	435514	1802595	4348413	4761490	393646	2478028	2192127	1259278	1944529	493775	1295494	307805	1734252	1620454	463035	2,1
99	600,33435	44,307	0,701	25	0,302	0,313	5,80E-03	0,331	0,28	4,50E-04	1	978545	4673452	3817923	1588527	5066792	259893	2829408	973869	4576719	6519298	10594830	7086520	10359250	2102244	1325111	3
100	442,58945	27,998	0,81	18	2,36	1,812	1,00E-03	2,293	1,395	1,80E-04	0	2355216	4468555	3618647	4726352	4542938	1776975	5800400	2674204	1266653	1080764	2275893	882441	2124828	3498401	2560252	2,87
101	608,34778	47,558	0,767	47	2,965	4,894	3,50E-02	3,558	5,83	2,90E-02	1	740974	1927891	1620548	4924623	2087199	1244889	4287890	1354796	665544	378579	2231156	170290	1685802	8520731	3722656	2,11
102	410,89105	21,631	0,728	49	0,122	0,129	3,50E-03	0,112	0,113	1,40E-03	1	412153	3574480	3601722	847627	952636	2080988	755668	1491664	11846872	7540203	11372962	14988486	19058382	996204	1075461	2,43
103	516,94318	30,816	0,705	44	2,403	2,848	3,20E-02	2,534	2,919	2,80E-02	1	617784	3052942	4219543	2195988	202319	3981958	2733555	685568	746908	981705	1112312	188884	1233184	1882824	918099	2,99
104	600,00049	44,307	0,687	37	0,295	0,322	5,50E-03	0,321	0,292	4,00E-04	1	465242	4802408	3921486	1553900	5180125	228833	2831000	957444	4631977	6612577	10751135	7190724	10563767	2142274	1336262	3
106	687,32306	56,724	0,595	24	0,475	0,675	1,70E-01	0,512	0,651	1,70E-01	1	254143	3579008	2419746	1785550	2867954	258281	2493788	174574	3794141	6474918	5211625	2017610	225666	2024736	969282	2,99
107	896,45105	50,175	0,719	42	2,245	3,219	6,60E-02	2,5	4,215	6,90E-02	1	476337	1865766	4162420	3549404	265115	3946663	2223512	980278	1589604	475966	746363	187547	1338816	1444570	560928	2
108	1029,97632	56,524	0,829	13	0,444	0,684	1,90E-01	0,491	0,644	1,50E-01	1	236996	3990215	2920293	1573681	3309438	244645	2757152	255628	3437276	7875650	6700517	2072831	345024	2027897	836160	2,03
110	689,11237	43,627	0,818	7	1,719	3,97	4,00E-01	1,996	4,466	2,80E-01	0	51888	956461	1834855	4878678	1453206	38259	527315	487703	123720	307744	1255618	54253	1790647	1881123	31203	4,99
112	653,02887	38,523	0,876	11	1,947	2,745	1,00E-01	2,104	2,726	5,50E-02	0	828345	3089530	3244678	4424030	260914	2241410	1519014	434630	1068736	572411	1046722	209062	2013958	2214475	866447	3
113	737,37677	51,164	0,821	5	11,121	12,707	6,10E-04	14,347	15,601	8,90E-04	0	671726	4459297	3234800	1685268	1391946	1240573	4833679	1715749	123121	264169	446537	106885	236988	2960180	4000126	3,88
114	489,9404	48,028	0,717	45	2,69	3,312	1,00E-02	3,043	3,035	8,80E-04	1	1763473	4594067	3515966	4103870	3767162	1905302	9723821	4393095	660092	1246056	2828063	520802	2198389	4002698	2337168	2,88
115	425,76672	26,106	0,959	38	2,055	1,749	6,80E-03	1,909	1,304	4,50E-03	1	4220946	4496907	3936719	3324915	7875425	2882490	6490357	2663716	1797114	962391	3706995	1640278	2831929	4425872	4650433	1,88
116	506,25592	30,484	0,667	11	2,667	2,574	7,20E-03	2,762	2,381	4,10E-03	1	2206404	4337549	3025092	3809607	713037	1270139	2818558	661428	790453	669888	1358431	457573	961887	2528990	1236308	3
118	583,54578	56,469	0	1	6,315	22,265	3,90E-01	6,973	24,363	3,80E-01	0	4785000	113531	25806	23096	56137	12988	27406	36498	71754	62104	144055	108449	18482	15164	17320	3,98
119	750,12958	51,267	0,701	25	0,285	0,454	4,20E-02	0,337	0,489	2,00E-02	1	88180	2737082	4735239	989667	2812788	18676	354626	3549713	1900055	7020273	4875271	4417702	10426717	833719	203087	3,96
124	612,63831	39,082	0,913	4	0,309	0,299	3,10E-04	0,324	0,272	1,80E-05	0	764103	4967968	3281645	1248039	5117256	268122	2416290	588488	5622132	6111569	9541821	7754804	8165387	2645483	1666100	2,99
127	937,96533	54,892	0,993	37	1,066	2,849	9,30E-01	1,392	3,366	6,20E-01	0	46224	1413386	541515	4966969	403082	1808779	344356	220294	495365	893985	2904245	109056	348848	186999	193879	2,02
130	937,46436	54,923	0,981	24	1,059	2,758	9,40E-01	1,38	3,236	6,20E-01	0	62619	1348013	546288	4796219	396507	1731794	352448	267022	494656	937875	2765094	134472	333349	184908	192782	2,05

131	649,271	19,986	0,79	51	3,927	3,658	1,90E-03	3,518	3,093	3,10E-03	1	232980	992552	2116819	1792472	1004525	2534948	1639014	709588	181788	261754	451349	280508	469193	763747	1130778	2,88
133	714,34558	44,915	0	1	3,879	5,029	4,90E-03	4,326	4,711	9,80E-04	0	978024	3409775	2912765	4535811	4094690	1519722	8512379	3576983	580656	671288	1799047	263366	1162300	3983193	1202222	2,54
134	682,70483	51,261	0,903	17	0,901	1,058	7,40E-01	0,995	1,187	9,90E-01	0	104476	2259437	3831974	2628363	3470702	744199	1982140	2960700	2699072	1496563	2897136	595920	3038422	610516	728800	3,51
135	541,95209	51,258	0,597	26	1,48	1,883	2,70E-01	1,719	2,3	1,50E-01	0	256293	1761934	4062339	2979023	778472	2893022	3174725	2583302	766069	761157	782061	2664758	1859733	1150348	594888	3,12
136	409,72424	28,114	0,922	16	0,293	0,263	4,80E-03	0,276	0,22	1,70E-03	0	585974	4333287	3929421	1868844	3518477	376536	3872683	2379514	5062492	6187430	9699849	9852605	11242094	2569646	1171898	1,91
137	416,75192	32,203	0	1	2,71	2,064	3,20E-03	2,763	1,862	1,40E-03	1	1670350	6091670	7228230	4653838	3319651	1737769	6544533	3257596	1444482	1578141	1708300	859072	1860553	3846255	2030479	1,78
139	415,25275	35,739	0,597	2	0,955	0,648	8,20E-01	1,033	0,892	8,90E-01	0	4979656	2177815	2610164	3620341	2441477	5059318	2469249	3244855	4830493	3440688	4863676	2714164	1819102	3377571	3774144	1,57
140	686,98883	56,724	0,724	5	0,474	0,68	1,70E-01	0,508	0,648	1,60E-01	1	201592	3147933	2128509	1564778	2594717	217465	2209931	182408	3324333	5692930	4582286	1740806	231528	1780579	740653	3,02
141	797,8634	59,06	0,8	30	4,776	9,852	3,50E-02	5,082	11,872	3,20E-02	1	191063	467995	753431	4724274	1378951	819295	1246341	198602	785075	136329	524492	64812	30252	3222235	1716186	2,04
142	750,3797	51,267	0,785	17	0,29	0,444	4,10E-02	0,345	0,475	1,90E-02	1	168080	2488268	4196804	963982	2544163	71134	385065	3047288	1712897	6326861	4413211	3975266	9200659	771959	232275	3,91
143	861,38568	43,602	0,877	57	1,528	3,364	4,80E-01	1,755	3,732	3,50E-01	0	37681	828978	1194842	3585122	1274654	33790	414966	370984	124501	283173	1291231	53794	1200839	1186010	97810	3,97
144	499,74667	51,358	0,972	35	1,645	2,771	2,80E-01	2,016	3,131	1,20E-01	0	364445	1223560	4420975	4540750	651957	2035022	2045623	1260934	977010	498745	1088268	282144	2687719	1194900	472293	2,19
145	525,94012	34,963	0,901	3	2,953	9,687	4,70E-01	3,526	11,842	4,40E-01	0	3617911	100949	50904	43743	48252	35320	32740	50981	118989	66351	178778	241204	86000	41203	60433	2,99
147	721,71832	43,647	0,64	50	1,171	1,718	6,90E-01	1,318	1,748	4,50E-01	0	47717	1468915	4055093	2756147	2854414	1986650	2400419	7359584	664520	3488556	1785728	1234536	3167482	925564	370407	2,99
148	436,19992	19,877	0,635	49	1,685	4,505	5,30E-01	1,382	3,344	6,50E-01	0	102227	719686	2556177	1369444	21458214	100370	464492	5183653	745229	1748573	2223573	812785	4797152	2735877	118725	4,27
151	517,2771	30,816	0	1	2,393	2,64	2,60E-02	2,517	2,674	2,30E-02	0	542646	2568589	3538956	1961774	262086	3328446	2342412	613042	628608	819670	940207	241582	1045511	1616726	813729	2,79
152	724,35803	41,453	0,685	10	3,167	7,618	3,70E-01	3,834	10,071	3,50E-01	1	3183326	114616	97912	114790	88013	66352	180587	238228	134997	121100	148389	155855	113355	93705	90140	2,69
153	649,6048	19,986	0,792	20	3,931	3,647	3,10E-03	3,516	3,104	4,80E-03	1	195765	822245	1818914	1455963	717199	2166615	1287887	563008	151218	224713	359264	241300	355241	573482	868690	2,94
155	450,25168	44,291	0,526	26	0,332	0,307	3,90E-03	0,365	0,272	1,30E-04	0	670248	4369766	4188419	1923030	4337366	296660	2949968	1232084	4707939	6158198	8144486	6830869	10373256	2386793	1720013	3,82
156	689,85419	33,684	0,868	62	0,247	0,26	9,40E-03	0,257	0,247	3,80E-03	1	229079	3066982	2768346	1113550	2241192	141011	1955288	1076033	3525105	3861544	8716026	7008226	7976126	2045272	690752	2,21
158	606,57172	21,146	0,836	18	1,756	3,03	2,40E-01	1,71	2,923	2,70E-01	0	110564	365435	870031	2491752	525514	1432453	316475	570128	215441	204139	194247	576056	1063403	788554	442254	6,25
159	437,754	29,683	0,777	16	1,956	3,22	2,10E-01	1,952	3,403	2,20E-01	0	387400	2565663	3948649	652647	561584	637844	382793	117498	1080684	791102	165125	642120	446761	268411	2703875	2,14
160	487,20444	19,986	0,712	25	2,894	2,377	2,20E-03	2,621	1,936	4,50E-03	0	361605	1209207	2329346	2058859	811214	2526338	1557302	920699	314745	469066	535928	389860	734823	1144610	1228341	3,89
161	849,04956	54,448	0,831	11	1,098	3,098	9,00E-01	1,507	3,939	5,70E-01	0	25206	839693	534444	4072283	276025	1745294	312217	311896	271846	706920	2559171	55883	235492	169024	124608	3,71
162	798,36481	59,06	0,771	7	4,741	9,644	3,70E-02	5,049	11,658	3,30E-02	1	183931	406281	637361	4139716	1139894	717876	1057090	172615	673630	123433	442057	71722	31796	2798068	1478044	2,04
163	639,35034	51,035	0,793	23	4,702	8,99	2,40E-02	6,819	12,811	2,70E-02	1	3370614	464535	1602064	230740	218649	604726	1095511	263737	147857	117027	222886	133883	782831	2629232	2728586	2,02
166	658,8772	58,367	0,977	2	9,649	30,785	3,60E-01	9,726	30,934	3,60E-01	0	3313341	35566	17540	29867	63705	10839	47324	25921	32218	55087	29016	34922	33721	12880	12250	2,29
167	394,74323	26,961	0,763	29	2,991	2,694	4,50E-04	2,784	1,962	7,10E-05	0	2056616	3625981	3325323	4438625	4371120	2033763	6248818	4036649	837609	701311	2125590	749895	1512771	3368058	1950515	2,01
168	765,37115	26,046	0,797	101	0,193	0,208	1,60E-05	0,171	0,181	7,40E-05	1	351885	2922382	1710308	551445	2133233	101854	642489	219452	3983350	5726914	5131442	6384471	5394619	1094728	538806	1,95
169	495,29251	44,539	0,863	35	1,89	2,177	6,20E-02	2,187	2,107	1,00E-02	0	2199743	3393303	2911399	4120203	4658566	1055550	5266158	2393784	867591	870566	3394585	617946	2652523	3578085	2189994	2,08
171	542,28601	51,258	0,394	2	1,642	1,739	1,10E-01	1,953	2,027	4,80E-02	0	267041	1548787	3445278	2513693	668231	2370428	2595611	2047471	663228	755494	736089	1347082	1643713	959566	481666	3,05
174	442,92334	27,998	0,57	3	1,827	1,422	1,20E-02	1,775	1,117	4,70E-03	0	1867277	3701476	2846529	4567186	4078500	1441522	4850430	2209512	1149699	1720024	2353209	887899	2271190	2987629	2069657	2,81
175	526,27386	34,963	0,669	3	1,629	3,867	5,80E-01	1,914	4,761	5,20E-01	0	3159029	208321	122264	123134	150531	109400	170306	139226	214538	334180	315899	317898	190509	148798	141821	3,13
176	400,2403	34,714	0,489	53	1,696	1,884	1,00E-01	1,858	1,972	5,30E-02	0	1524665	3422570	3985419	2482533	552104	1885719	1217804	285800	1277065	1077532	1011749	336929	1914788	1866706	1828313	2,35
178	721,38446	43,647	0,345	2	1,143	1,677	7,30E-01	1,294	1,723	4,80E-01	0	65511	1084712	3213097	1967578	2091539	1612835	1963712	5541992	469664	2799240	1435245	959054	2470690	737475	318161	3
179	450,50201	44,291	0,585	29	0,325	0,311	4,10E-03	0,357	0,278	1,50E-04	1	538816	4283558	4037827	1764241	4172662	240958	2789022	1139148	4477533	5977660	7930469	6656099	10088056	2289086	1605171	3,9
181	446,21613	29,3	0,797	23	1,315	1,195	2,90E-01	1,327	1,251	2,90E-01	0	2172330	1925927	2893334	4427517	4048039	1516186	5226060	1671948	3393012	1686484	3902121	962998	2661250	4405550	4854135	2,5
182	500,80579	38,065	0,799	38	0,549	0,564	1,00E-01	0,568	0,534	9,00E-02	0	179835	2596387	2742844	3298665	1407686	1135953	1567883	1651945	3318245	1525742	3126724	2276494	5494452	1993785	715062	2
183	861,13568	43,602	0,914	10	1,564	3,522	4,70E-01	1,789	3,91	3,50E-01	0	23532	721153	955195	3124471	1071264	18937	353118	331762	104873	225500	1085431	39478	992982	1046432	13692	3,97
184	442,75854	26,788	0,873	25	1,501	2,777	4,40E-01	1,26	2,227	6,30E-01	0	452092	642054	456219	3563292	6211269	1797628	418628	2160850	2100868	226950	483190	1074506	1681294	634841	369209	2,1
185	464,25058	35,008	0,907	41	0,79	0,698	3,20E-01	0,794	0,703	3,60E-01	0	275337	3295158	3695533	3294781	2632933	1260374	2408838	2492531	3575642	2003257	3131817	1484125	3356841	1124325	921056	2,27
187	438,75668	36,924	0,763	24	3,083	4,014	7,30E-03	3,306	3,819	2,10E-03	1	1100128	3079038	1475158	1958730	1030005	648074	3917026	1575480	244640	711280	1198341	150287	600897	2277065	851501	3,56
189	601,86176	19,694	0,954	39	2,087	3,211	9,20E-02	1,845	2,489	1,10E-01	0	303383	943781	1192180	1138747	1782210	2852808	4086709	5690508	231271	654084	1279126	771778	2672831	2778662	2640861	5
190	477,58478	24,743	0,78	26	0,535	0,54	5,90E-02	0,492	0,481	3,70E-02	0	182558	3093743	3501471	2147970	2491974	1259724	2179456	1472348	3146026	3489051	5391488	1804478	2784010	612686	829762	3,04
191	564,99042	19,986	0,846	33	2,503	7,209	4,10E-01	1,992	5,311	4,70E-01	0	56602	661109	1933848	762012	32860161	99164	958508	6262506	561247	1958037	1954695	889040	3909115	2755944	71825	3,99
193	421,75916	28,995	0,683	40	0,705	0,61	2,00E-01	0,707	0,624	2,10E-01	0	2423840	2566918	1834334	3448302	1931682	4527523	4708294	5160151	4064085	8833521	4952883	3473019	4554714	7003316	2876483	1,82
194	575,84271	25,836	0,892	2	0,263	0,288	3,30E-03	0,232	0,243	2,80E-03	1	271734	2648894	2100884	708487	2379406	121230	1268666	423677	3132882	2772553	4952416	5654061	5639368	1106592	606064	1,99
195	438,50629	36,973	0,79	46	3,112	4,334	9,60E-03	3,36	4,212	3,00E-03	1	1125517	3146331	1382713	1823932	1042644	606237	4012184	1435678	190576	719069	1232069	129340	585497	2331730	874381	3,82
196	435,99973	19,877	0,56	3	1,675	4,483	5,40E-01	1,373	3,333	6,60E-01	0	79824	665451	2182098	1194151	18845965	71217	409476	4682616	635535	1523203	2049908	698852	4230498	2405378	71813	4,41
197	759,37018	33,234	0,744	67	3,366	6,209	1,20E-01	3,245	5,747	9,70E-02	1	214644	291224	267716	2878758	6290756	866478	920760	1720363	646399	119404	300096	394602	737116	930027	412196	1,98
198	660,06506	22,49	0	1	6,812	13,035	1,90E-02	6,093	11,355	1,30E-02	0	285358	2291953	2324356	143488	360210	2288989	4763202	4142745	33728	682672	196170	97539	299985	991163	256479	8,75
199	498,76306	29,726	0,727	26	3,009	4,169	1,90E-02	3,196	4,296	1,90E-02	1	638945	1643794	1174461	3325087	1122473	1085938	3350042	1077479	500259	346270	1405307	286363	1004591	5415563	2486211	2,46
200	522,95679	35,69	0,762	3	10,23	35,312	3,40E-01	12,361	43,149	3,40E-01	0	2935117	37389	28505	55747	61100	14972	44074	22977	19004	66256	12990	34361	27301	60878	11174	3
201	487,4549	19,986	0,742	9	3,011	2,52	2,20E-03	2,726	2,071	4,30E-03	0	319287	1070123	2046855	1808650	708853	2256924	1340911	810533	273585	391471	472779	303659	615193	975380	1046383	3,93
206	644,34814	42,643	0,794	32	21,365	19,64	3,00E-04	23,973	20,497	1,80E-04	1	1877721	1724145	3198557	1938731	2624087	4446644	6941575	7360543	179620	119736	290177	118821	214184	5634765	3673518	2,18
210	587,29456	53,307	0,942	5	2,879	7,682	4,20E-01	3,466	9,546	3,90E-01	0	3469207	169922	95881	82482	80323	101653	115697	76187	131936	113057	193399	184740	136341	83097	98664	3,99
211	654,64398	44,433	0,798	24	2,139	2,968	6,40E-02	2,452	3,167	2,50E-02	1	711172	2248964	2465736	2803173	439350	950636	629168	192522	742100	259803	819493	125134	1199607	1926577	1089087	2,99
212	826,71667	56,893	0,849	4	8,446	30,496	3,60E-01	9,701	32,918	3,60E-01	0	2614826	18913	14799	19958	65885	15609	21092	15366	17221	74114	27598	26508	21125	16147	11181	3,98
216	590,81519	36,309	0,818	48	1,755	2,186	1,10E-01	1,86	2,028	5,40E-02	0	1018332	2857269	2129883	3315365	2923426	698750	4228355	1631100	982943	589534	2827156	436278	1688661	2861510	1236695	2,04
217	523,29022	35,71	0	1	0,528	0,936	1,40E-01	0,591	1,117	2,40E-01	0	2706740	190314	227315	171043	1151839	118248	223304	170328	1419698	414824	1187156	1019370	1755786	962096	206000	2,62
218	826,96674	56,688	0	1	8,433	28,507	3,60E-01	9,703	30,791	3,50E-01	0	2444799	36792	18102	21985	62803	10951	23441	30974	16558	58159	36023	26908	20946	20685	4295	3,88
223	404,20422	28,086	0,697	10	1,27	0,713	1,30E-01	1,261	0,761	1,70E-01	0	3103173	2057235	2840159	2181663	997341	1924428	2921743	3421339	2058903	2571658	2315974	1202588	2092378	3686038	2876379	2,68
227	544,31708	31,489	0,835	35	0,237	0,237	5,30E-03	0,24	0,217	2,10E-03	1	247157	2478958	1818537	707656	1649942	127038	1848014	866903	2763796	3561836	6509451	5818556	6153356	1405452	625794	2,02
228	659,95422	22,476	0,961	2	6,564	12,481	2,00E-02	5,848	10,813	1,40E-02	0	284145	2161477	2217679	137438	342702	2120845	4472609	3972207	42654	667943	193153	100464	279557	912162	232793	8,82
229	525,62408	34,225	0,835	2	7,688	29,643	3,60E-01	9,333	36,211	3,60E-01	0	2456950	28188	24916	34421	23330	22495	25076	33449	13111	94571	14507	39300	13657	28346	15828	3
231	856,52844	68,33	0,581	17	0,515	1,58	4,90E-01	0,538	1,487	4,80E-01	0	99219	3834389	63871	313217	385842	121497	4227530	82390	210966	3642919	5106796	29813	90349	193455	28922	10,41
232	457,78729	27,668	0,691	13	6,152	10,24	7,60E-02	6,385	11,532	1,10E-01	0	2870002	335910	397210	517856	326355	305161	566311	293730	75735	141537	119302	55830	102558	366452	110820	1,93
234	690,35657	33,506	0,864	6	0,231	0,247	1,00E-02	0,241	0,234	4,50E-03	1	237413	2215842	1929930	745481	1566902	132095	1338364	724138	2631205	2755709	6628861	5202277	6007091	1385024	438881	2,49
235	654,30981	44,462	0,805	9	1,973	2,509	7,20E-02	2,252	2,659	2,80E-02	0	716378	2122160	2441110	2665669	456988	940417	632618	243029	778140	288418	814103	244840	1208611	1860200	1080906	3,2
237	564,73956	20,013	0,942	4	2,48	7,132	4,10E-01	1,969	5,253	4,80E-01	0	43583	571210	1683684	659642	28647015	129146	813363	5436153	495191	1732722	1710440	795669	3410732	2311449	110665	3,86
238	476,86606	20,303	0,765	52	2,718	3,003	2,40E-02	2,344	2,81	4,50E-02	0	522603	441912	2216393	2304553	1574145	5578483	1633915	2894487	1084715	376919	926715	1083828	631614	999254	4144141	2,83
239	725,40289	27,766	0,791	73	0,235	0,245	3,20E-03	0,227	0,231	1,60E-03	1	193652	2237290	1214252	458130	902324	128538	1399888	360304	2008789	4525542	5188875	4183387	3515971	827817	1419564	1,92
240	587,82092	24,276	0,815	11	8,349	19,555	1,80E-01	8,521	21,211	1,90E-01	1	99905	89144	89230	2274045	71517	125126	91986	28226	67536	18801	41467	57524	33548	664277	121445	2,29
241	577,44769	58,958	0,771	10	2,115	4,575	2,20E-01	2,78	5,658	1,00E-01	0	256848	2819302	2429904	2607989	41027	48447	2949681	444244	59488	1535161	1029437	119232	186504	702028	93193	6,02
244	849,383	54,462	0,869	12	1,152	3,112	8,50E-01	1,544	3,783	5,20E-01	0	32933	695472	495415	3427066	226395	1294397	280252	292512	256004	564732	1928001	81601	219712	143455	138510	3,44
245	765,87231	26,051	0,869	4	0,182	0,198	2,60E-05	0,161	0,173	1,00E-04	1	257997	2147627	1174664	409447	1506549	74705	456801	150793	2982933	4343061	3924238	4891679	4093684	774855	400659	1,98
247	504,75455	24,384	0,91	11	1,033	1,577	9,40E-01	0,98	1,337	9,50E-01	0	139157	573256	2516416	1408166	899439	524308	914834	2807526	367206	971635	666137	981525	2064474	395180	254182	2,89
248	594,34314	44,539	0,667	6	4,212	5,768	8,80E-03	4,842	5,82	3,20E-03	1	558547	810189	703425	3009913	540849	964328	1490924	807887	149873	265379	498575	75051	242565	886043	601190	2,12
249	602,06183	19,694	0,93	5	2,086	3,193	9,00E-02	1,846	2,471	1,10E-01	0	239322	733065	991044	914490	1322599	2203641	3182236	4440966	190592	528673	958732	611396	2093065	2201537	2056424	4,98
251	820,47345	34,168	0,848	74	0,533	0,588	9,60E-02	0,558	0,56	7,30E-02	0	112835	2061509	1693891	1419292	598457	585878	957424	853051	1600340	761092	2099549	1563489	3134054	1133767	339047	1,98
252	705,14124	59,035	0,988	4	1,35	2,51	5,60E-01	1,265	2,058	5,90E-01	0	1533251	6119763	370994	4103923	14690035	24493	10541276	3294873	1655614	3679498	5609324	50600	4963601	2410314	13595	11,98
253	617,93713	20,85	0,948	2	4,897	6,316	7,10E-03	4,381	5,538	7,50E-03	0	193477	795453	1558326	237371	200953	1139576	1122949	442141	46111	232372	173659	73143	143252	410513	446711	5,87
254	577,28088	58,958	0,87	4	2,061	4,291	2,20E-01	2,676	5,126	1,00E-01	0	284555	2722634	2357096	2575859	56497	84206	2889879	422217	91937	1497595	1027058	157011	191747	693416	134254	5,85
256	690,69421	51,382	0,753	22	6,794	13,843	2,00E-02	9,521	18,607	1,80E-02	0	2124727	321911	1466284	68681	98792	500901	976716	225205	38363	62639	114809	36807	344402	1937325	391450	3,4
257	564,8537	19,986	0,491	2	0,449	0,76	1,60E-01	0,391	0,632	1,10E-01	0	27719	401372	1293004	449443	174162	573773	434973	1740374	152689	2331187	752920	1174743	1660180	203562	148700	4,71
258	705,22339	59,01	0,805	21	1,342	2,494	5,70E-01	1,255	2,042	6,10E-01	0	1493755	6035111	357639	4038158	14624695	18965	10525582	3278836	1681229	3737414	5544717	41652	4930750	2392944	8668	12
259	560,27936	54,305	0,69	25	3,314	4,885	1,20E-02	3,793	5,776	1,20E-02	1	349278	1479898	994008	2972661	1078639	977676	2385446	775420	740179	183084	1119121	250035	244497	3961874	1840903	3,32
260	797,66199	52,58	0	1	1,658	2,268	2,00E-01	1,873	2,806	1,50E-01	0	131545	663309	2272705	2274588	298671	1087595	1547114	998984	868378	422743	663789	117181	928396	508479	167918	4
262	618,10413	20,819	0,913	6	4,78	6,101	8,10E-03	4,278	5,331	8,60E-03	0	192414	767119	1475031	199002	209362	1085253	1086967	388183	41263	216529	164028	79422	145242	375305	401242	5,22

263	625,64807	55,004	0,965	2	1,236	3,189	7,60E-01	1,573	3,629	4,90E-01	0	45312	915720	384277	3261611	195577	1177164	199276	106914	372577	492157	1492079	102317	182824	118434	128431	3,12
264	668,64105	51,265	0,738	2	2,626	4,018	4,10E-02	3,511	5,577	3,30E-02	0	1270370	1399121	2321735	733867	248538	503308	1717855	601990	553111	238078	855365	84841	1217731	3912573	2780994	3
265	625,31348	55,004	0,812	2	1,175	2,757	8,00E-01	1,474	3,059	5,10E-01	0	113090	962354	427918	3179124	225058	1167936	271593	164112	431598	545961	1544129	161648	230262	167551	168327	3,97
266	856,43805	68,33	0,773	9	0,505	1,576	4,80E-01	0,515	1,491	4,60E-01	0	103243	3873691	45531	263702	400335	31045	4192518	80770	127339	3672286	5100357	88937	85804	151066	27353	10,96
267	668,97522	51,265	0,798	6	2,63	4,022	4,10E-02	3,514	5,56	3,30E-02	0	1318560	1483997	2424061	726593	254232	526256	1795306	617760	569207	238091	882183	103317	1267427	4065340	2884334	3
269	801,09009	55,412	0,836	45	1,835	1,984	5,60E-02	2,042	2,826	7,00E-02	0	649781	1090832	1324536	1983843	3348457	1739412	1513496	2523188	1862291	564002	1578971	313874	739583	2101356	2291454	3,01
272	516,80176	22,577	0,789	43	2,155	1,972	6,80E-03	2,023	1,602	6,00E-03	1	1136995	2175677	1721667	1851737	1822846	910510	3067424	1828706	480733	467523	1453084	564166	962442	1373135	1044297	2
273	859,20056	60,772	0,468	3	6,752	25,523	3,10E-01	8,201	29,439	3,00E-01	0	3306210	143150	12960	11959	564905	20279	67900	13883	11706	56297	203254	19904	17669	13518	15760	3,99
274	521,30682	34,006	0,631	45	1,873	2,702	1,50E-01	2,217	3,211	9,80E-02	0	2340463	1572194	2381690	450969	385911	559255	95637	690685	782181	331080	1343629	299876	1239628	3716052	2780694	2,06
277	508,24042	39,923	0,843	16	4,448	5,495	2,30E-02	4,757	5,137	1,10E-02	0	383771	623996	471516	2677927	493717	603785	852746	583071	163381	134673	258847	106144	200386	588722	401416	3,07
279	523,27142	52,207	0,705	5	4,401	9,899	2,50E-01	5,644	13,063	2,30E-01	0	3401677	155177	132096	426723	98954	133216	166484	214405	91991	156340	92350	142150	99650	199511	199226	4,75
280	674,1875	21,762	0	1	6,396	12,854	3,30E-02	5,605	10,859	2,40E-02	0	187762	1446233	1170419	69585	129350	1108149	3387973	1771402	14463	397350	144314	55655	164562	548221	112281	8,79
281	445,90213	32,411	0,679	32	1,821	2,873	1,70E-01	1,849	2,711	1,30E-01	0	616293	2346873	2290442	2011872	1569275	1806740	6916380	4088421	445506	3256224	1280685	533237	1242239	1997804	965166	2,86
282	648,02197	52,712	0	1	9,165	26,954	3,10E-01	12,078	33,912	3,10E-01	0	2144565	38455	19201	41473	55581	35892	70692	86529	16884	37088	33973	20691	29362	20870	16117	3,15
283	859,45056	60,68	0,529	8	4,427	14,063	3,10E-01	5,334	15,223	2,70E-01	0	3737780	190891	24368	67747	685415	121752	102151	27677	30635	174794	276766	65652	30601	32394	131246	3,72
285	808,4256	42,154	0,87	23	3,763	10,676	2,60E-01	3,637	10,509	2,50E-01	1	82435	55837	49722	1745727	194066	51835	42750	68025	172201	15261	41902	27634	104116	306230	121268	2,01
286	675,10413	44,539	0,855	33	1,828	3,554	2,70E-01	2,121	3,921	1,70E-01	0	47108	571483	1258271	2442713	1099220	56874	284629	526699	94652	321266	588381	68740	988921	1215355	37022	4,98
289	647,68683	52,839	0,777	3	9,281	28,149	3,30E-01	11,876	35,501	3,30E-01	0	2146329	31055	18433	27625	46574	37469	60383	24663	18100	36878	26436	28472	20952	21835	14108	2,99
292	521,60718	30,837	0,682	4	3,697	7,603	2,50E-01	4,073	9,046	2,70E-01	0	2270756	115971	108749	108352	229935	164887	207769	207024	100125	120223	109037	78038	79896	108765	80630	2,9
295	498,5838	21,712	0,868	17	2,055	5,857	5,00E-01	1,835	5,281	5,50E-01	0	100529	50207	2089790	89440	58669	113895	97209	36562	183666	224565	45114	137659	81553	42945	84514	2,88
297	559,78888	44,433	0,765	16	4,005	5,756	2,60E-02	4,404	5,499	1,50E-02	0	278321	2413568	1247614	2285657	3924047	518648	608169	1083611	195485	244386	547035	162317	519603	614669	393020	5,66
298	1057,20422	59,01	0,835	7	1,907	4,201	3,20E-01	1,786	3,273	2,90E-01	0	482319	3497600	61887	1829901	7661229	9905	5881363	667186	741561	1261160	2798622	14878	760948	1177250	6004	7,93
299	784,66064	39,937	0,931	3	13,514	49,972	3,60E-01	16,101	62,636	3,60E-01	0	1840990	7867	7315	5862	7485	9008	6962	5278	29267	6454	11134	15901	7750	6171	8619	2,93
300	722,05237	43,647	0,971	2	1,261	1,988	5,90E-01	1,415	2,03	3,90E-01	0	39280	813002	2525813	1726128	1782946	1093636	1294593	4964395	392036	2040621	974843	667576	1854796	493960	216692	2,99
301	634,84668	40,18	0,816	26	4,776	12,934	2,70E-01	5,03	12,843	2,50E-01	1	25391	174620	92504	2431872	54172	417362	55188	138036	60031	103873	122492	19376	56741	43249	30438	2,08
302	750,12952	57,85	0,735	47	0,363	0,603	1,00E-01	0,355	0,476	6,80E-02	1	151155	4072292	484225	2322931	3981897	211247	4961650	120321	3000884	7076137	8767683	6781163	219137	1674292	785512	3,98
303	559,95514	44,45	0,806	9	3,157	4,772	4,60E-02	3,339	4,943	3,10E-02	0	240041	2319164	1242031	2195490	3847788	512415	491909	1143585	140324	210973	495054	744970	435196	501261	300701	5,6
304	942,07928	68,33	0,541	7	0,523	1,686	5,20E-01	0,534	1,632	5,10E-01	0	51551	3212067	22176	214902	306937	7828	3162751	57966	79628	2419043	4281256	13990	59718	118185	8125	9,97
305	392,22394	34,511	0,849	16	2,821	3,344	2,00E-02	3,004	3,055	8,90E-03	0	469771	589766	562345	2406394	624975	634946	1231133	749910	197074	248469	514568	169188	349066	558576	512197	2,62
307	621,29523	31,619	0,764	7	1,964	4,898	4,90E-01	2,238	5,737	4,60E-01	0	1919758	102917	80438	65653	136800	73989	78282	95466	132246	88312	214161	137553	114296	72085	70844	3,47
308	493,92062	23,494	0,741	21	0,842	1,315	6,80E-01	0,775	1,126	5,10E-01	0	61896	498404	1900082	643943	708814	619979	696939	2487259	240852	1497086	624457	844172	1591728	290947	172821	2,92
311	555,8078	38,73	0,804	29	0,923	1,566	8,60E-01	0,965	1,571	9,40E-01	0	52879	887975	2408912	1356904	1381436	931938	1619477	5102946	369787	3061056	1194313	1014924	2155972	429016	220952	2,18
312	1057,32861	58,958	0,779	25	1,903	4,194	3,20E-01	1,77	3,263	3,00E-01	0	485986	3536454	49248	1866851	7717774	7111	5886254	538308	742083	1288894	2767099	10701	775858	1164846	2760	7,06
314	454,26617	37,192	0,914	28	4,8	6,08	2,40E-03	5,18	5,729	8,80E-04	1	685901	1347979	1896870	2392235	2203010	1419845	4211706	5608696	266061	329215	878821	197766	863554	2839407	1735209	2,3
317	560,61322	54,305	0,833	4	5,078	8,224	7,20E-03	6,19	9,53	9,20E-03	1	230631	1004324	803433	2221549	884209	655698	1858689	613418	263546	122175	657783	92519	163107	3438736	1483102	3,31
318	597,63629	50,332	0,651	24	2,187	3,308	7,80E-02	2,381	4,287	9,00E-02	1	209310	958049	2102391	2005536	156971	1998313	935702	555575	979008	202119	346946	127626	670436	943168	307764	3
319	941,98065	68,33	0,572	6	0,522	1,668	5,10E-01	0,531	1,601	5,00E-01	0	46958	3236935	22734	214747	338820	13747	3217512	53330	84935	2533751	4247777	38659	63476	118237	9901	9,94
320	674,90442	44,539	0,896	7	1,806	3,444	2,70E-01	2,087	3,777	1,70E-01	0	23103	534105	1124383	2202499	1001673	55659	266708	495627	101043	317658	533131	56360	886946	1107167	35560	4,97
321	690,36041	51,382	0,771	3	4,461	6,82	2,50E-02	5,965	8,505	2,00E-02	0	1903206	319454	1301554	102029	115827	476580	900605	229989	87960	124288	158397	116953	348939	1726467	387515	3,58
322	401,20749	33,234	0,824	25	3,219	4,208	5,40E-02	3,308	3,973	4,30E-02	0	224904	321905	448682	2517266	606059	279840	706836	833095	205590	171580	229074	142382	327130	729607	258149	2,54
325	523,02106	52,286	0,591	4	6,179	17,638	2,80E-01	8,311	23,437	2,70E-01	0	3380519	132472	106408	291776	43320	58920	144931	220393	48463	117720	63987	21319	114852	123966	24554	4,02
327	782,1532	55,249	0,791	45	0,321	0,478	5,70E-02	0,333	0,443	4,00E-02	1	78459	2099591	1737475	646640	1869648	82355	2182765	320031	1053333	4065384	4698263	4192370	1178063	516481	211930	3,99
328	705,30652	59,045	0,934	3	1,33	2,473	5,80E-01	1,243	2,013	6,20E-01	0	1225941	5072320	300313	3387918	12199048	28788	8812797	2670264	1445973	3058748	4749197	46265	4125470	1982551	36499	11,98
329	438,89368	34,063	0,683	38	0,321	0,29	2,30E-05	0,334	0,279	6,10E-06	1	227316	2351295	1475503	840177	1214498	109276	803339	270759	2566146	2886943	2631904	2618511	3600829	1326314	567292	2,97
333	586,33875	20,088	0,809	48	0,099	0,146	1,60E-02	0,087	0,132	1,90E-02	1	87787	555182	912637	194209	415888	63207	67472	69515	2343540	2229982	2132960	5898995	2987055	114626	598605	2,01
334	430,98346	31,852	0	1	1,524	6,857	7,30E-01	1,774	8,097	6,60E-01	0	1939377	65256	33360	40416	34767	32929	53781	30962	16065	666342	30528	22117	22668	31804	47543	3,64
335	820,97443	34,168	0,806	6	0,535	0,588	1,00E-01	0,561	0,559	7,70E-02	0	108381	1698068	1394930	1176798	503951	497268	782656	719601	1308430	643264	1747625	1260303	2617607	942866	283274	1,99
338	431,23441	31,852	0	1	1,315	3,401	7,00E-01	1,453	3,938	6,30E-01	0	1908857	140491	115687	140622	206251	119116	224319	189215	151088	690481	130166	181769	130378	171797	158987	2,53
339	617,05627	51,762	0,871	28	14,993	26,097	1,50E-02	19,173	28,725	7,70E-03	0	149166	2139726	1620815	82870	186359	247392	1300637	326689	14097	106402	54932	21754	44340	557073	631408	4
341	651,05304	59,04	0,969	12	1,346	2,531	5,70E-01	1,26	2,066	6,00E-01	0	1290105	5062618	293684	3122833	11932575	13941	9368002	2630307	1273551	3278116	4586590	32085	4046769	1857074	6226	12,9
342	398,23999	22,861	0,674	13	1,155	1,447	6,70E-01	1,127	1,456	7,30E-01	0	591714	1158397	2011280	2120320	245274	1005846	659673	424718	1275429	422347	687938	360681	1408006	858034	518067	2,33
343	458,24866	22,2	0,898	18	1,453	2,247	3,80E-01	1,437	2,185	4,00E-01	0	622461	446745	2020460	1565725	1282542	1306022	1521325	999828	1071353	318100	1178668	691561	2352843	1125207	5420823	2,12
344	573,33209	41,566	0,873	3	7,094	19,819	3,00E-01	8,629	25,32	3,00E-01	0	1914604	41104	70026	77458	25248	43037	68644	65031	33739	48765	20788	40010	25340	48137	39316	2,09
346	426,22952	28,946	0,746	2	1,105	1,358	7,70E-01	1,135	1,389	7,20E-01	0	2355058	449939	524681	814115	451309	372456	502961	1877722	383159	877697	964585	549841	864441	336056	360834	2,31
347	490,75168	34,511	0,771	27	0,264	0,529	2,00E-01	0,27	0,514	1,90E-01	1	137908	1833106	1490657	2073316	2086920	1331818	896778	5973567	2836364	18217253	138929	4112206	7576128	858792	672707	2,64
348	553,76367	35,044	0,702	3	0,597	1,124	4,30E-01	0,608	1,12	4,40E-01	0	89402	2128528	780374	2184256	1020541	499714	933578	3108977	58836	4333705	49534	2119399	2875235	448990	66497	5,89
349	650,97687	59,025	0,966	3	1,345	2,515	5,70E-01	1,262	2,043	6,00E-01	0	1334245	5041102	316314	3143930	12003768	34625	9441918	2650628	1308074	3318756	4663680	44803	4008827	1895339	22241	12,94
350	497,72601	42,539	0,724	9	0,951	2,339	9,40E-01	1,046	2,37	9,40E-01	0	45541	314217	320662	2342036	2153760	700753	701439	28058	388117	111451	357889	472757	2321248	211886	127532	2,58
351	642,33038	57,42	0,724	6	2,131	3,679	2,00E-01	2,492	4,488	1,50E-01	1	260855	197283	131717	2060788	528938	150310	306518	107756	311091	178217	420831	239803	58328	471807	932801	3,47
354	500,24792	51,358	0	2	0,619	0,833	2,50E-01	0,768	0,907	4,10E-01	-1	196048	1324699	3463431	2621696	1020430	996774	1189501	1533561	870013	2366204	1823876	1514791	4264430	766721	312340	4,06
355	415,26862	46,562	0,845	58	4,498	5,315	4,50E-03	5,012	4,708	1,00E-03	1	266301	545403	1036846	2178072	1255142	722877	2658394	1418826	111477	240672	336101	154924	445613	1004936	506734	1,9
357	521,94214	30,783	0,635	3	3,718	7,784	2,60E-01	4,114	9,264	2,70E-01	0	1862611	93130	97638	82120	201179	124325	133370	140486	80472	94699	87144	61313	65874	93263	68176	2,94
358	759,87134	33,204	0,921	2	3,36	6,535	1,40E-01	3,239	6,013	1,20E-01	1	123132	169776	191397	1876374	4591636	555061	605230	1068348	426814	72878	214753	265987	515811	596182	277907	1,98
359	624,30341	35,814	0,746	4	2,841	6,547	3,50E-01	3,304	8,115	3,40E-01	0	1776495	96048	93239	75952	92096	82075	98904	92127	94732	128622	71178	81477	80111	78608	105722	3,57
361	422,75778	21,112	0,885	16	2,164	3,209	1,00E-01	1,994	3,005	1,40E-01	1	197888	189271	1723432	924853	351918	1606102	302353	1550859	236219	167086	367063	793182	611941	136738	2432798	2,39
363	861,16455	51,254	0,844	32	2,056	3,183	1,10E-01	2,642	3,712	3,50E-02	1	300197	875495	1659095	362106	1097887	2411200	3210781	4932887	414438	534749	1887776	380241	1093532	1157656	1721509	3,98
366	717,89154	54,637	0,975	3	8,92	28,477	3,50E-01	10,667	33,072	3,40E-01	0	1823686	26685	8943	19752	55025	8510	22046	22922	18080	36489	23216	16859	18235	16831	9459	2,45
367	426,75122	22,964	0,82	10	0,84	1,25	6,50E-01	0,774	1,05	4,60E-01	0	56373	453652	2191706	1295707	918958	874325	340238	3014407	497835	917169	877054	1556103	1898072	272915	234479	2,3
368	750,38049	57,787	0,784	13	0,365	0,601	1,00E-01	0,36	0,472	6,90E-02	0	190180	3621394	450718	2042402	3549599	225875	4354993	138778	2616430	6299182	7818875	5982943	213878	1466531	704768	3,94
369	808,92676	42,154	0,89	10	3,74	10,716	2,60E-01	3,604	10,485	2,50E-01	1	66703	48961	47268	1411755	160705	43638	33301	56159	142425	7470	32295	23895	88986	244751	93665	1,99
370	445,69785	19,643	0,845	7	0,626	1,087	3,60E-01	0,543	0,956	2,70E-01	0	21508	53047	969660	500416	152189	574825	44546	774143	172143	482203	203175	1094755	755145	35514	266419	3,45
376	467,75217	30,877	0,602	3	2,034	3,659	3,70E-01	2,244	4,397	3,60E-01	0	1537009	111894	151434	254114	109548	80893	109181	95775	135208	110047	132643	126892	147974	127491	78679	3,19
377	897,55676	68,348	0,962	11	0,495	1,545	4,70E-01	0,51	1,484	4,50E-01	0	53364	3575338	22671	361706	251680	6354	2338447	80354	164028	2685935	3912021	11170	126060	126169	9103	9,97
378	450,25433	31,069	0,905	21	1,319	1,683	4,30E-01	1,436	1,856	3,40E-01	0	1796887	476507	849896	1175624	215339	198259	287697	232398	511741	319813	890387	343660	382046	650511	573032	3,02
379	429,75027	27,217	0,862	12	0,347	0,667	2,00E-01	0,313	0,568	1,70E-01	1	182881	1353957	1070739	1964951	1192021	509856	649772	4122379	1188954	7823503	182464	2309444	5550798	499241	291010	1,98
380	618,271	20,799	0,291	2	3,859	3,837	6,60E-03	3,428	3,37	8,50E-03	0	145376	527519	1023373	168924	156139	725934	741794	274813	89618	190887	126152	96275	114310	248621	750895	5,38
381	553,9303	34,587	0,975	5	0,583	1,134	4,20E-01	0,592	1,133	4,30E-01	0	19954	2021169	717092	2112443	948639	456988	865252	3012625	32233	4207256	38420	2014966	2743926	349074	30084	5,97
383	725,90393	27,766	0,841	7	0,201	0,227	3,80E-03	0,189	0,201	2,00E-03	1	144117	1622110	857503	342860	635910	90466	960708	275349	1453006	3442165	3985925	3074402	2597328	597201	315159	1,95
384	872,38367	45,036	0,983	40	1,608	2,791	3,20E-01	1,877	3,295	2,20E-01	0	16020	1086152	704857	1452567	764270	31738	66830	1009431	87120	790557	701520	31968	553928	1780548	50880	3,86
385	445,94833	19,643	0	1	0,611	1,068	3,40E-01	0,53	0,944	2,70E-01	0	18201	47706	846436	452963	121210	493989	45472	659787	146862	436888	174660	994606	661381	31629	232051	3,27
386	410,73288	34,092	0,688	3	0,686	1,121	3,50E-01	0,789	1,325	5,80E-01	0	2122238	506044	403568	213863	408766	228973	395559	243567	448447	637011	973315	403679	1111896	240499	144141	2,48
387	706,39996	38,515	0,746	48	7,517	9,373	9,10E-03	7,629	9,065	5,30E-03	1	106214	277474	484822	1464125	253804	627581	1157948	554468	88532	59833	79089	26607	105030	352544	119339	1,97
389	515,77496	36,9	0,793	14	1,666	2,657	2,60E-01	1,684	2,517	2,20E-01	0	818776	122335	176322	2122223	1593567	522532	259776	1225913	596984	127279	198037	444624	911788	567818	181835	2,31
390	554,32513	43,512	0,755	3	8,541	26,193	3,10E-01	10,953	34,956	3,10E-01	1	2054457	39605	36579	50765	34681	35778	49438	54349	14049	26460	27077	50275	25140	56857	30204	2,49
391	613,80817	29,3	0,873	48	0,407	0,493	6,40E-02	0,395	0,443	3,80E-02	1	107408	1427134	1035969	1677107	981418	333638	1087462	664407	1608395	1863966	3652597	923597	1625793	378401	190235	2
394	782,40369	55,275	0,805	22	0,323	0,467	5,70E-02	0,337	0,429	4,10E-02	1	146937	1913232	1537696	590889	1671871	113147	1947453	295198	971817	3605669	4299141	3854463	1090376	470709	229011	3,99
396	710,37732	51,266	0,815	10	2,424	4,025	8,90E-02	3,347	5,745	6,80E-02	1	1157366	726702	1790851	615107	175402	224686	508824	254376	406996	156343	557004	144099	961222	2896559	2438263	2,27
397	843,62653	44,568	0,911	50	1,678	3,103	3,10E-01	1,932	3,437	2,00E-01	0	24346	501457	732962	1506900	868214	25527	230466	338370	72474	235359	584780	27138	582850	793769	19656	3,96
398	500,42154	57,173	0,782	44	0,473	0,718	1,70E-01	0,507	0,686	1,50E-01	0	107765	1895234	1825659	1079703	1652652	92219	2571421	239597	1095827	3534521	3380454	3006485	140693	678626	410305	5,91

399	566,02393	37,997	0,686	7	5,05	6,205	6,50E-03	5,446	5,847	2,90E-03	1	324104	425476	308329	1528644	291169	474136	579041	289459	84518	75247	188229	54628	105742	435258	478731	3,97
400	661,95129	51,265	0,885	15	1,999	2,911	1,40E-01	2,41	3,648	8,00E-02	0	88053	1267840	2236717	185729	2666556	562195	61642	863772	669166	408193	403261	159961	857521	486781	1570104	3
401	498,91772	21,712	0,829	5	2,023	4,837	4,60E-01	1,841	4,447	5,00E-01	0	91922	122177	1502488	74138	29903	91911	57761	36892	130165	156406	66838	106296	82568	76546	109820	2,89
402	475,75534	55,3	0,802	33	1,103	1,607	8,00E-01	1,233	1,448	5,10E-01	0	110921	2243696	1432755	1039552	1331740	176962	1225993	940503	403421	1264373	1455603	268086	634202	243859	132561	3,88
404	780,09222	19,549	0,902	15	1,821	4,356	4,30E-01	1,741	4,107	4,60E-01	0	5612	162730	140793	611440	15009	1080518	11680	105447	32052	123580	209276	26295	200369	8566	13173	3,8
407	468,00272	30,877	0,786	2	5,775	17,905	3,50E-01	6,601	20,987	3,50E-01	0	1520940	24561	27452	22094	38697	18204	31652	31231	28113	53507	20716	24845	25819	28403	23854	3,88
408	649,68237	55,272	0,751	35	2,181	4,621	2,70E-01	2,623	4,883	1,80E-01	1	85575	1108075	958444	4238488	521239	1188667	233544	288031	344243	407565	901839	112952	282003	160303	154929	3,57
409	897,65631	68,346	0,963	3	0,507	1,579	4,80E-01	0,522	1,51	4,60E-01	0	57792	3403968	20410	342038	250000	7456	2207003	80123	158867	2487323	3630211	21276	110163	125402	4106	10,02
410	558,30328	49,744	0,938	4	0,502	0,523	4,10E-02	0,557	0,46	1,70E-02	0	327487	4026753	2402481	2379396	2045560	580769	1983927	1623879	1923208	2918673	4623095	2602460	4851092	975180	641773	3,19
411	501,30688	38,065	0,842	27	0,784	0,785	4,20E-01	0,828	0,755	4,80E-01	0	262804	1828295	2086185	2754217	716369	1188219	2006188	832033	1953738	832235	2081624	1275587	3190189	1838688	1117966	1,96
412	451,55441	31,211	0,982	2	2,496	5,138	2,70E-01	2,847	6,022	2,60E-01	0	1615978	138325	107347	297175	94472	130788	117536	67458	104196	121721	148245	34249	188416	310074	100393	2,74
413	472,76959	38,144	0,852	32	2,516	2,17	1,10E-03	2,723	2,098	2,50E-04	1	1032092	1788056	1357149	1584634	2456438	856511	1933005	763299	543442	293954	1071414	383924	753358	1959642	1596042	2,04
414	597,97021	50,332	0,69	9	2,238	3,41	7,30E-02	2,425	4,434	8,80E-02	1	189894	880248	1919304	1825202	138571	1850722	850664	516982	897178	186310	316493	110634	569546	873124	264043	3
418	661,61688	51,265	0,874	4	1,991	2,861	1,40E-01	2,398	3,628	8,20E-02	0	73219	1120063	1991517	175489	2412013	525359	54007	754622	615146	363885	380981	147324	749988	448114	1434649	3,01
419	548,32013	34,143	0,834	8	0,598	0,578	1,10E-01	0,627	0,569	1,10E-01	0	141838	1932362	1867632	1485891	776985	743954	1097342	1029290	1917995	804343	2171905	1475043	2987341	1614624	496059	2,95
420	675,30457	44,539	0	1	1,816	3,257	2,50E-01	2,082	3,48	1,50E-01	0	49931	467295	939883	1891202	848021	58988	232118	411692	114575	258806	456092	80693	718210	946824	68082	4,81
421	816,1427	68,348	0,83	4	0,496	1,518	4,60E-01	0,511	1,444	4,40E-01	0	61182	2991115	23121	319840	226089	10780	2179440	75565	144881	2545517	3226435	24961	107204	107014	6740	10,89
422	566,61823	54,625	0	1	13,151	10,142	3,30E-04	14,327	11,422	1,10E-04	0	239969	1118699	892089	1801809	854221	394538	1057126	609761	71233	85275	63886	53758	43991	772818	626796	3,17
424	640,57068	56,724	0,881	25	1,252	1,841	5,70E-01	1,376	1,94	4,10E-01	0	65883	1744587	2091635	1167422	1756922	387330	1440698	313925	812638	1151371	1362706	417943	88583	279917	351419	4
425	690,04706	58,244	0,858	18	0,366	1,101	5,00E-01	0,565	1,707	6,30E-01	0	82392	2285208	2799116	2095951	38074	103172	83628	1370878	210898	11454874	874648	376685	215686	671071	71755	5,99
426	564,85413	29,571	0,85	14	0,509	0,521	5,00E-02	0,497	0,465	2,70E-02	0	81382	1379042	1166994	896378	994279	367954	694749	547071	1357762	879339	2107988	791283	1531784	431719	233368	2,39
429	458,01541	40,997	0	1	6,839	22,01	3,70E-01	8,946	28,967	3,60E-01	0	1745488	9138	14467	9189	52832	7191	16506	19785	11402	29842	29867	35767	32033	20499	4919	3,95
432	540,3161	55,714	0,928	30	3,358	5,317	3,20E-02	3,794	5,468	1,80E-02	1	724062	865284	188504	1944867	449234	382767	295628	73208	210277	78470	395780	184806	54507	664528	615697	2,12
433	493,84647	51,81	0,892	26	17,507	34,042	1,60E-02	22,808	39,376	8,90E-03	0	121138	1928267	1489686	61095	153537	183721	1134603	331134	8618	93107	34005	10306	37448	452870	568554	4,96
434	458,26624	41,087	0	1	2,104	4,39	4,10E-01	2,561	5,697	3,70E-01	0	1803147	97052	115754	99855	188198	98404	119412	110494	133719	126422	137285	101090	172372	113869	76617	3,58
436	897,45752	68,353	0,951	2	0,516	1,55	4,80E-01	0,542	1,454	4,60E-01	0	101229	3183075	48179	355330	240960	45827	2124365	92886	179792	2313984	3466511	70255	121488	130852	25879	9,83
439	444,22159	34,381	0,891	18	5,022	5,12	6,50E-03	5,125	4,5	2,80E-03	0	431705	1247395	1709907	1793049	860659	1316091	4065894	2938949	266289	415131	359423	176106	364750	1145893	375722	2,65
441	523,28192	23,845	0,778	7	0,749	0,919	3,80E-01	0,759	0,977	4,20E-01	0	1391371	512380	1302814	568054	207221	612004	597355	568078	953040	2526180	1096416	1055464	1560529	2573736	2434393	2,02
442	649,34851	55,412	0,476	2	2,138	4,316	2,50E-01	2,584	4,558	1,50E-01	0	140064	1074269	836402	3887997	497747	1126756	275284	454696	343134	422683	877658	108798	267188	167314	174653	3,67
443	403,21964	26,788	0	1	3,669	3,168	4,80E-03	3,338	2,361	2,50E-03	1	467208	738824	885536	2083634	763410	917576	2692981	1936742	282996	354204	406977	214333	392476	1034419	595100	3,27
447	651,86169	53,118	0,785	3	1,333	5,531	8,00E-01	1,893	7,738	6,10E-01	0	94386	21986	1758893	13374	67390	11309	31940	60108	18036	572579	15419	18517	158185	15445	11385	9,29
448	427,50403	34,469	0,846	2	7,617	27,776	3,60E-01	9,442	34,812	3,50E-01	0	1629582	20999	17387	19452	18040	11141	40675	21998	10630	57068	23533	9804	17349	13799	10375	3,71
451	816,05243	68,348	0,767	8	0,491	1,447	4,40E-01	0,505	1,344	4,10E-01	0	59332	3140580	72950	343390	380924	12210	2270950	99719	162212	2724207	3491194	119744	133666	124499	13099	11,29
455	509,9198	40,923	0,718	39	3,985	5,352	1,10E-02	4,392	4,994	4,50E-03	0	231620	583787	765647	1717838	957217	626876	2757178	2684283	178121	214822	415259	149548	586025	1518194	460497	2,97
456	494,2547	23,494	0	1	0,875	1,249	7,30E-01	0,816	1,061	5,60E-01	0	179100	417140	1441061	542403	429203	528492	616533	1911350	211207	1159568	506867	674302	1213970	290049	236117	2,86
457	746,03113	43,341	0,771	8	2,183	6,395	5,20E-01	2,74	8,277	4,60E-01	1	1324759	57695	25296	15700	113964	18520	23211	14949	61204	49415	120540	90201	50966	15168	16436	3,03
462	660,28687	22,484	0	1	5,503	9,117	2,20E-02	4,897	7,724	1,50E-02	0	175313	1128354	1107032	100962	212509	1115223	2444593	2078632	49047	353722	151460	80811	181815	443073	184272	8,72
463	687,70996	56,724	0,489	15	3,222	6,926	1,00E-01	3,364	6,586	8,50E-02	0	36969	2811265	3565864	2715023	5959625	21075	1550034	86158	48752	83512	1412879	586017	588997	697180	86563	6,14
464	460,23862	33,629	0,668	26	0,353	0,274	1,30E-03	0,37	0,258	2,60E-04	1	635391	3019269	2535398	1270549	2069525	340308	1696227	1043349	3386113	3264140	5109520	5180613	5457339	2080981	1103038	2,85
465	694,61652	36,285	0,778	38	3,622	4,564	2,10E-02	3,894	4,459	1,60E-02	0	102189	239362	238562	1289872	174265	1547668	1525372	1469918	131427	139483	339465	160955	276436	552728	450935	3,89
466	475,50464	55,384	0,785	8	1,079	1,59	8,50E-01	1,204	1,423	5,60E-01	0	71461	1933490	1219801	855260	1159706	158656	1082294	773427	352971	1114408	1271009	235019	537719	214726	111170	3,95
467	412,74777	26,788	0,633	8	0,94	1,209	8,60E-01	0,892	1,133	7,30E-01	0	126483	662808	1465453	1722151	319056	525946	684746	635617	479232	931031	1091378	253762	768140	329459	154218	2,06
468	785,15216	68,33	0,951	4	0,501	1,566	4,80E-01	0,509	1,49	4,60E-01	0	70142	2334388	27933	163968	248070	7529	2670071	56266	69494	2375355	3133425	23218	73800	97460	6389	11,83
471	558,05823	53,79	0,802	3	1,739	3,249	2,90E-01	2,198	3,678	1,30E-01	0	47375	2094228	727745	2699138	259393	1326267	434319	1172411	163466	705591	1203690	108661	420781	153967	135359	3,89
472	538,80914	41,782	0	1	2,892	7,23	4,00E-01	3,526	9,487	3,80E-01	0	1663127	45257	89952	47495	66538	63094	50440	60390	80344	67933	57303	81178	90212	56586	37544	2,88
473	835,44916	49,752	0,748	63	0,243	0,307	1,90E-02	0,269	0,295	8,50E-03	1	132681	1347216	1027551	409521	1514581	69462	645855	328694	1450175	1928150	4297035	1883834	3467707	569916	273525	2,14
476	523,7785	24,648	0,791	3	1,261	1,565	5,10E-01	1,323	1,757	4,70E-01	0	1942840	937110	1153978	344877	100341	314694	212006	259155	677320	1226516	708923	515330	702795	2126216	2267009	1,98
477	546,95819	49,864	0,841	10	1,66	4,401	5,40E-01	1,92	4,582	4,20E-01	0	136176	72516	1611624	99213	58303	225169	116526	137506	112716	362799	53650	128678	113198	58390	45045	3,78
480	499,26437	29,726	0,75	6	2,623	2,871	9,80E-03	2,719	2,933	1,20E-02	0	518766	1351886	1232130	2037914	769567	638286	1781860	1004514	536797	266181	907427	267790	686383	3228383	1414718	2,26
481	574,0011	40,102	0,695	14	5,93	9,844	4,70E-02	6,475	9,876	3,90E-02	1	144999	1404938	1246339	483686	359015	92785	92960	98731	44040	62615	129764	37020	92910	383917	37530	3
482	718,39246	54,683	0	1	2,637	8,721	4,40E-01	2,973	10,306	4,20E-01	0	1395027	47045	34107	46645	88751	23556	46005	49377	21362	67187	33721	170738	43554	28261	16364	2,69
483	861,41498	51,255	0,885	25	2,009	2,998	1,10E-01	2,564	3,459	3,40E-02	0	336372	843436	1585972	417612	1099982	2312958	3016172	4734365	441464	524257	1835027	431403	1028461	1106464	1669638	3,96
486	712,08032	62,064	0,973	5	0,958	2,6	9,50E-01	0,978	2,321	9,70E-01	0	181202	1750815	30863	255116	1782355	26463	3564855	147171	76150	1321651	2296374	18548	389076	108540	9926	13,83
487	872,13293	45,051	0,966	9	1,599	2,832	3,40E-01	1,871	3,369	2,30E-01	0	14361	911625	598820	1248599	643080	20092	50522	881281	64234	681232	601645	19099	487716	1536367	23518	3,98
489	545,28607	55,432	0,769	13	0,428	0,811	2,30E-01	0,479	0,735	1,90E-01	0	148375	1961878	1700651	1110765	1042022	229387	3573348	304753	823265	5824163	3887898	1406202	686152	462648	272551	5,5
492	460,73523	38,144	0,59	12	0,257	0,562	2,40E-01	0,267	0,553	2,40E-01	1	160088	1370695	1089082	1536590	1756389	1173558	640807	5254661	2682668	17479629	183806	2706876	5210872	781360	785722	3,7
493	488,72858	22,678	0,732	12	1,109	1,301	7,50E-01	1,07	1,331	8,40E-01	0	494659	1073020	1560974	1189733	163694	1152689	377425	126760	1157449	456234	635533	238712	897188	605049	765477	3,2
494	476,27264	25,836	0,838	29	0,311	0,292	5,50E-03	0,281	0,242	3,00E-03	0	290149	1273005	1203861	547455	1191194	136147	668582	282162	1877898	1309138	2100599	2359046	3205585	727041	437286	2,37
496	599,38049	59,103	0,907	10	1,857	2,986	1,80E-01	1,92	3,935	2,50E-01	0	734225	1051918	1224991	1653142	1071801	1732151	457716	996579	1935511	286883	592928	518897	34520	2708832	881783	2
497	687,87756	56,665	0	1	2,987	6,251	1,10E-01	3,031	5,949	1,00E-01	0	44436	2423645	3049656	2337762	5221909	19203	1336187	86329	42402	53177	1179147	503051	765266	585074	88349	5,92
498	649,30298	45,917	0,814	28	0,208	0,233	2,30E-06	0,229	0,281	1,50E-03	0	138152	385445	1507775	159086	474870	143919	221725	203326	2058530	1857817	1812077	2485382	1829795	509894	442488	3,94
499	769,24384	59,01	0,982	13	1,453	2,793	4,90E-01	1,351	2,255	5,10E-01	0	597013	2941482	170995	1904974	7249871	9052	5058084	1402848	731967	1548111	2605551	33522	2084509	1016186	3586	11
500	699,30206	39,303	0,883	5	2,336	7,161	4,80E-01	2,959	9,132	4,40E-01	0	1566847	71649	41187	25771	72496	51059	25730	41334	64922	61160	175613	59904	58487	30895	35835	4,11
501	566,95197	54,585	0,678	3	12,534	10,114	3,40E-04	13,651	11,029	1,20E-04	0	219039	932722	750974	1532781	762227	292193	832442	539236	57802	79150	47609	60986	36010	641781	554846	3,12
503	428,26947	28,995	0,94	3	1,616	2,607	3,30E-01	1,689	2,689	3,10E-01	0	124010	741980	575617	2098016	224360	1838145	215326	388796	250989	477537	669439	146666	482334	157550	188915	1,95
504	500,58881	57,231	0,85	17	0,466	0,706	1,60E-01	0,498	0,669	1,50E-01	0	100364	1652904	1504735	947467	1389978	82071	2213466	187571	968666	3055937	2916347	2665252	102598	599727	365816	5,86
505	569,67035	34,469	0,827	4	7,974	23,369	2,80E-01	9,615	28,751	2,90E-01	0	1306128	41852	42049	25845	23017	43899	51747	43274	13776	38125	22194	15077	14263	41911	29831	2,96
507	440,72443	23,3	0,86	14	0,455	0,671	7,30E-02	0,446	0,697	6,90E-02	1	95882	370162	865717	1817875	208088	441060	213547	542253	1543023	413161	939536	956571	1835378	167058	457683	2,15
508	617,30676	51,749	0,882	3	4,133	4,353	2,20E-02	4,835	4,698	9,00E-03	0	191054	1533431	1168219	142723	183990	243994	857749	298220	100778	149444	113098	159825	145345	435575	470249	3,76
511	834,44043	71,237	0,952	2	3,176	9,493	4,10E-01	3,113	9,224	4,00E-01	0	1438146	57224	23159	37743	44497	18259	74196	22210	25338	86339	60419	76496	30410	37632	18940	3,05
512	643,40674	59,534	0,781	14	1,849	2,836	1,70E-01	1,885	3,699	2,60E-01	0	675002	1065767	1263017	1600220	1173031	1205444	535061	1186466	1858111	319947	601506	431073	59452	2535964	851077	2
514	636,29932	53,087	0,572	6	1,712	3,295	3,80E-01	2,127	3,992	2,80E-01	0	1599690	198819	308392	595878	127322	122126	161309	175364	121292	223329	359098	68781	228385	61043	76502	3,29
518	545,45264	55,363	0,783	18	0,417	0,801	2,10E-01	0,457	0,729	1,50E-01	0	71697	1776787	1591357	889868	935357	82696	3172196	259806	725277	4832062	3619673	1264305	705854	361588	157358	5,98
524	494,26236	38,476	0,888	31	2,955	3,93	2,60E-02	3,465	4,706	2,80E-02	0	1498472	878809	1613695	655725	209250	612906	1341887	393721	442949	217687	630932	174158	739092	3454659	2370465	2,96
526	453,71576	25,43	0,883	11	2,243	3,795	2,10E-01	2,228	3,966	2,30E-01	1	119041	110258	187943	1526867	116074	197255	189361	226930	214679	87362	164033	80934	205682	435099	267692	3,23
527	506,23953	25,346	0,853	20	1,575	1,639	1,50E-01	1,533	1,53	1,60E-01	0	270205	593332	1290851	980710	208462	545027	317113	299540	367462	223502	343027	219237	521228	500241	268361	2,8
528	761,39429	54,108	0,478	3	2,295	4,622	3,10E-01	2,824	5,491	2,50E-01	0	1347929	68477	167813	56894	119981	65478	100780	259218	67079	147466	80367	84996	138976	41618	153871	2,76
529	612,9726	39,22	0	1	0,273	0,287	7,30E-04	0,287	0,263	7,60E-05	0	227877	1608423	958054	344781	1668478	105711	700733	170278	1895227	2003129	3441277	2726354	2787997	771100	467306	2,99
535	843,37628	44,63	0,919	9	1,579	2,735	3,40E-01	1,81	2,968	2,10E-01	0	28147	465409	686585	1331960	803437	39673	220496	303512	92997	244437	538362	43756	552981	732712	39177	3,94
537	575,27716	51,944	0,898	22	3,281	4,962	2,20E-02	4,461	6,8	2,20E-02	1	358011	1095003	914895	1551180	764756	802098	1732605	713204	249206	214833	832987	140785	726878	4126777	2144959	3
538	690,2135	58,216	0,973	4	0,359	1,085	4,90E-01	0,557	1,695	6,20E-01	0	65723	1940165	2449122	1763374	41716	76768	61924	1204864	170417	9938240	797575	320693	191291	543309	61454	6
539	391,19604	21,712	0,559	5	0,763	0,963	4,80E-01	0,728	0,919	4,10E-01	0	283350	1234720	1629488	959396	190690	836161	474627	191078	598597	1836832	1026650	283121	1000996	707164	737249	2,81
540	424,90265	38,251	0,767	76	3,162	7,919	2,40E-01	3,32	7,663	2,10E-01	1	459888	827874	1570913	1160336	17463928	497211	1095738	4175673	366053	583123	1382961	363825	2109138	2274772	864807	2,55
541	815,96191	68,346	0	1	0,487	1,482	4,50E-01	0,502	1,416	4,30E-01	0	47795	2806454	23845	299368	268192	5830	1996804	70476	128468	2437803	3082721	18555	115774	108233	6689	11,12
542	785,23535	68,328	0,901	5	0,501	1,581	4,80E-01	0,51	1,516	4,70E-01	0	41001	2368274	23315	163888	254010	6025	2617270	49919	59690	2320217	3144113	15377	68928	91439	6364	11,87
543	742,36353	48,594	0,873	9	2,662	3,37	8,10E-02	2,918	3,747	5,70E-02	1	192118	329232	224537	1376652	225471	149337	184388	223692	162385	128162	133430	93283	193469	648493	229858	4,57
544	905,81024	76,539	0,87	4	3,121	9,294	4,10E-01	2,975	9,297	4,20E-01	0	1141954	43002	21600	34535	40232	7127	48421	20889	22767	70620	43640	66033	24042	52134	7458	3,02
546	604,80804	40,647	0,701	9	3,366	3,201	2,50E-03	3,587	3,829	1,70E-03	0	237989	502307	760034	1405786	281752	1006617	1091158	771692	342493	210257	211210	90012	255687	1095639	316942	3,9
548	574,33447	40,068	0,771	14	3,754	4,649	5,20E-02	4,027	4,542	4,00E-02	1	396863	1348146	1155218	453187	355013	90665	85182	93206	93043	98210	151852	115820	127881	385409	43222	2,85

550	687,43317	66,084	0,697	28	1,393	2,763	5,50E-01	1,43	3,572	6,10E-01	0	682284	858752	253239	1494977	48041	1417401	543193	59162	1812352	263154	465965	373219	88915	2219936	788090	2,03
551	602,85242	47,329	0,662	13	0,806	1,627	7,50E-01	0,928	1,717	9,00E-01	0	56027	1228197	764243	1536378	1688359	1148683	2815233	533761	153486	549101	1172880	561916	3988956	426887	165898	2,01
553	712,00891	61,597	0,911	3	0,971	2,575	9,70E-01	0,995	2,271	9,90E-01	0	186317	1730396	60993	279005	1752833	22958	3483330	207582	76869	1336925	2218568	28954	385191	111687	22697	13,41
554	574,94275	52,013	0,848	24	3,639	5,822	2,00E-02	4,994	8,073	2,10E-02	0	303518	1095358	924206	1600029	753636	804104	1790955	718744	216016	193345	777501	88588	712375	4264208	2212077	3
555	766,77997	61,965	0,949	6	0,975	2,633	9,70E-01	0,99	2,335	9,90E-01	0	142073	1422659	27054	199768	1425113	8858	2741416	115979	69518	994711	1790837	19057	295645	91710	7191	13
556	649,84723	68,115	0,792	10	4,429	11,071	1,90E-01	4,649	8,859	1,10E-01	1	103830	438393	174417	1576406	47783	83889	3104033	71573	67204	271343	222266	41443	60828	220645	52541	4,53
557	427,75455	34,469	0	1	6,675	18,321	2,60E-01	7,926	22,446	2,70E-01	0	1489305	44678	59305	68822	52294	47525	85014	60129	24774	57037	20714	19841	29135	53261	62101	2,73
558	602,32269	39,541	0,677	12	0,374	0,937	2,80E-01	0,395	0,948	2,80E-01	1	156188	953161	1191340	1460469	1584348	977484	1004520	8123763	1162148	11893795	195563	2682212	5917653	518171	384197	2,28
559	758,63495	29,164	0,813	47	1,319	3,487	7,00E-01	1,282	3,166	7,20E-01	0	34582	121527	123186	1049050	2055398	428178	286354	2810689	200788	59689	186716	431232	2013426	535186	182452	2,89
560	831,02271	66,571	0,929	3	3,146	9,146	3,90E-01	3,244	8,212	3,70E-01	0	1232237	61648	19042	35727	32409	19800	118873	19703	26551	83497	72171	22630	48360	37055	16764	6,6
561	529,80432	20,819	0,836	3	4,464	5,836	5,40E-03	3,995	5,061	5,10E-03	0	156872	469443	955305	149439	171033	620472	803691	300284	28374	182608	105228	49827	106782	271963	322434	6,81
563	563,78699	29,571	0,728	21	0,251	0,306	7,90E-03	0,246	0,282	7,30E-03	1	234274	1197920	714393	261319	1587967	115281	361125	137125	2167014	901892	3050479	2988244	2639321	884211	400742	2,09
565	689,88007	58,216	0,371	3	0,329	0,841	3,80E-01	0,482	1,189	4,50E-01	1	92923	1762359	2279379	1804553	34165	366192	61163	1117454	157810	9069949	673706	2415994	176306	603944	103338	5,51
567	621,39362	59,312	0,758	17	1,857	2,802	1,60E-01	1,918	3,753	2,40E-01	0	744586	1121284	1308695	1657157	1181559	1581729	520097	1268907	1947990	321902	633660	494948	89131	2686328	881090	2,05
569	393,20947	33,34	0,763	5	1,734	3,447	3,90E-01	2,1	4,652	3,40E-01	0	1628139	445381	351643	871311	603751	1615361	419860	339043	1090202	754491	1326561	341929	313917	557436	6439747	2,56
572	769,15356	59,01	0,99	7	1,418	2,701	5,10E-01	1,321	2,166	5,40E-01	0	614100	2984219	190334	1895594	7294396	20428	5076292	1396695	783492	1653692	2674170	37959	2081358	1033326	7551	10,99
573	486,79068	34,278	0,938	2	4,522	14,413	3,40E-01	5,418	17,545	3,30E-01	1	1350299	45181	30875	35864	31971	25419	45210	42220	20035	87617	18646	43487	20381	25971	87027	2
574	665,41986	60,391	0,727	17	1,848	2,777	1,60E-01	1,875	3,649	2,60E-01	0	617468	946478	1285938	1549028	1236639	1139419	562081	1162824	1781814	294007	543573	406655	109159	2318445	766282	2,75
575	640,82117	56,618	0,719	2	1,256	1,804	5,60E-01	1,378	1,895	4,00E-01	0	71297	1378071	1681498	905157	1397882	306572	1107715	278669	637258	913410	1054251	319459	109743	222886	269111	3,9
584	439,22781	34,063	0,315	2	0,372	0,287	4,10E-05	0,387	0,276	1,80E-05	0	285318	1611537	1048364	825502	786910	202903	625096	292991	1745507	1909290	1678583	1706181	2358238	907903	407967	2,59
586	855,11237	20,987	0,984	20	2,811	2,783	4,40E-03	2,582	2,351	6,10E-03	0	151245	489151	972348	441725	373720	940790	556192	351364	60756	189330	184013	162815	287424	310610	385404	3,97
590	712,15186	61,965	0,954	2	0,957	2,584	9,50E-01	0,977	2,301	9,70E-01	0	212767	1508278	34189	223291	1556155	11389	3085069	134058	66874	1124671	2033746	18037	347087	99632	7683	13,95
591	687,86652	25,546	0,91	6	5,887	21,514	3,40E-01	6,815	25,59	3,50E-01	1	1020136	20786	35810	6990	12556	18745	26797	31223	5673	55908	24034	6765	10010	24275	8143	2,23
593	532,57941	51,661	0,896	12	5,662	8,386	1,20E-02	6,861	9,245	6,20E-03	0	201832	387102	305503	1585229	427214	318441	620046	395757	70135	58341	159236	18003	121693	344665	253858	2,99
594	786,39557	47,578	0,576	42	2,28	2,338	3,20E-02	2,514	2,478	2,00E-02	1	215414	370564	263151	1228320	713208	273941	367000	279071	202189	131981	218480	206198	335819	939654	342189	2,98
595	440,86621	23,311	0,699	3	2,984	8,51	3,80E-01	2,657	7,739	4,10E-01	0	58686	45962	1367917	50685	34950	139618	43035	19335	112259	73922	22492	59383	38146	31785	35605	2,86
598	547,29285	49,908	0,68	9	1,72	3,161	3,50E-01	1,994	3,26	2,30E-01	0	218777	168783	1527719	228400	110829	370433	189148	307398	172140	383918	98471	152550	167470	127767	103662	3,69
599	804,40637	55,151	0,799	10	3,575	4,559	5,50E-02	3,887	4,794	4,10E-02	1	166306	1077045	793698	345636	227811	98490	88175	62532	71812	113794	101908	114889	67856	452489	50160	2,23
601	766,70337	61,914	0,99	2	0,961	2,569	9,60E-01	0,973	2,227	9,60E-01	0	138419	1378635	33835	206296	1387315	17437	2742726	120848	78398	965813	1808357	56192	288849	98779	19359	12,9
603	603,50366	50,088	0,683	31	0,419	0,571	7,20E-02	0,468	0,56	4,70E-02	0	105521	1465948	2068533	422854	917904	71218	817976	1189721	828916	1235286	2028976	1782689	3448827	488564	267497	4,99
604	785,31781	68,33	0	1	0,501	1,573	4,80E-01	0,509	1,514	4,70E-01	0	34402	1996987	13847	129962	247417	4004	2173558	40021	46506	1986971	2622110	4698	62736	79551	9080	11,91
606	745,89819	45,333	0,796	2	1,884	6,323	5,90E-01	2,497	8,33	5,00E-01	0	1229083	52490	18640	8583	91110	13289	18314	12254	44845	65293	185897	47953	47599	12705	19210	2,46
607	835,94971	49,744	0,813	27	0,239	0,307	2,20E-02	0,266	0,297	1,10E-02	1	108235	1141920	857903	363033	1305248	57678	556258	267185	1218730	1626280	3780290	1621774	3034995	505133	234862	2,15
608	438,24054	25,43	0,989	2	1,132	1,376	7,30E-01	1,067	1,316	8,60E-01	0	281047	416352	325522	1673957	217089	163889	938355	491966	324525	563032	478878	359965	410260	173137	154273	3,93
609	517,30347	22,577	0,897	2	2,156	2,082	9,70E-03	2,004	1,701	8,50E-03	0	445553	1022321	761871	844401	819189	388456	1510465	824681	198584	186792	651581	349576	393330	620352	437725	2,02
610	630,84436	55,79	0,835	3	6,569	20,872	3,60E-01	7,015	23,372	3,50E-01	0	1063654	15414	14607	10318	19399	9767	23885	20870	24967	25996	13552	18917	7998	12851	10390	4
614	460,57266	33,629	0,711	6	0,354	0,3	6,80E-03	0,367	0,269	1,80E-03	0	179198	1211761	1055436	651224	743755	188287	903024	1348977	1572089	1344107	2791077	2288565	2817483	962634	400818	2,78
615	494,04639	51,749	0	1	16,807	31,986	1,70E-02	21,905	37,024	9,10E-03	0	84051	1305953	1004585	48484	110745	120601	770357	211572	4632	62944	24389	9345	27752	297332	384650	4,95
616	545,77026	24,367	0,875	3	1,834	4,312	4,80E-01	1,687	3,959	5,20E-01	0	83149	81163	1225816	67362	125727	73491	73834	59012	90200	95353	70836	185103	79185	51351	69247	3,06
618	826,4657	56,688	0	1	5,49	17,209	3,60E-01	6,094	18,878	3,50E-01	1	1135107	20547	16184	23669	39672	10757	27088	10059	14270	34767	26251	32945	11322	20442	9144	4,14
620	714,38275	52,159	0,871	3	2,964	8,683	3,90E-01	3,924	11,269	3,50E-01	0	1035750	51506	27967	25368	43095	25808	62439	47195	28592	97277	29882	45010	31053	34727	20565	3,37
623	692,73633	58,958	0,899	7	2,283	4,488	1,50E-01	2,985	5,368	8,00E-02	0	114899	1290542	1073018	1324615	34109	41486	977744	203187	43194	510253	468796	69240	105416	340212	65726	5
625	709,44629	67,229	0,727	40	1,382	2,624	5,50E-01	1,427	3,481	6,10E-01	0	685094	743869	267849	1440726	87773	1296459	549987	143823	1721791	278939	443400	347055	111440	2052377	754478	2,46
626	529,94696	20,799	0,907	8	4,009	5,045	5,90E-03	3,577	4,363	6,00E-03	0	141379	460054	907087	163939	149107	625489	731532	291529	38782	193940	112074	55946	104715	279304	303716	6,34
629	586,29034	21,694	0,861	28	1,43	1,774	3,20E-01	1,391	1,636	3,40E-01	0	207104	496959	1030241	756781	83647	607105	112335	54286	304042	168079	304648	162743	455258	262978	377446	2,09
631	450,58871	31,069	0	1	1,418	2,599	4,90E-01	1,587	2,914	3,90E-01	0	1111309	202319	456966	596314	54825	38426	43298	27904	247087	124011	484186	70141	171755	292455	287063	2,89
632	586,66077	49,562	0,703	26	2,73	3,163	9,10E-03	2,934	3,601	6,50E-03	0	177323	525888	973930	1582139	264413	1049714	1144124	1364731	239179	121406	250207	603814	337542	1048961	343819	2,97
634	694,86584	36,254	0	1	3,224	3,716	1,90E-02	3,464	3,608	1,50E-02	0	120282	198835	189972	897530	159893	1125616	1064791	1031494	113602	121146	264625	139192	219494	395477	348019	3,86
635	603,70386	50,088	0,837	9	0,415	0,56	6,80E-02	0,463	0,548	4,40E-02	0	95315	1297989	1731322	374995	786674	58308	686961	984313	711713	1059142	1764884	1551646	2931170	418072	228581	4,99
637	577,36688	59,061	0,797	7	1,694	2,853	2,70E-01	1,772	3,774	3,30E-01	0	677845	970329	1139519	1556700	961003	1765741	354951	359684	1880664	281621	586413	513329	63929	2625650	859438	2,15
640	690,04742	51,273	0,846	10	0,266	1,079	4,80E-01	0,358	1,442	5,30E-01	0	40582	108361	2630940	43626	37159	20457	56146	3179979	25319	10905484	33819	24978	570370	24533	7769	5,82
641	780,34332	19,528	0,97	8	1,755	3,875	4,20E-01	1,657	3,617	4,60E-01	0	11370	142427	127248	501449	29085	889204	27208	96553	38580	113762	188102	28003	165464	13088	36465	3,75
642	458,28848	27,828	0	1	2,351	3,783	2,00E-01	2,48	4,207	2,30E-01	1	1297611	160647	166466	212394	122932	140572	177985	183521	106045	98549	157484	74592	147820	162939	123363	2,04
643	699,55243	39,303	0	1	2,238	7,514	5,30E-01	2,899	9,716	4,70E-01	0	1276486	61671	20275	9009	45696	13008	13833	12970	38660	49440	146469	45766	50870	14499	15257	3,94
644	656,52295	20,345	0,861	10	0,685	1,299	4,20E-01	0,598	1,153	2,80E-01	0	9985	470029	273373	22970	24714	17290	117119	78072	58963	230973	252325	137389	112024	34405	36914	3,91
645	544,27856	43,155	0,849	31	0,668	0,487	4,60E-02	0,74	0,568	1,60E-01	0	1101982	1617343	1095298	675902	614352	774043	527914	436169	1095727	791244	1081149	1445686	1425497	623811	336546	2,97
646	510,29187	33,621	0	1	0,755	0,909	3,60E-01	0,851	1,045	6,10E-01	0	1113823	464320	286462	299693	114591	201330	276841	186298	343414	257620	604311	426098	645088	333598	160860	2,67
648	663,38135	28,087	0,839	41	2,735	2,605	3,80E-03	2,622	2,073	1,30E-03	1	378049	892413	551130	973485	1159810	313516	1245341	376613	206862	172711	440704	172590	283365	756805	332928	1,96
649	578,82117	26,524	0,822	9	13,517	18,24	3,60E-03	12,361	16,477	2,20E-03	1	587111	501713	1086814	257110	332439	2346919	2844871	3138908	23614	146615	99901	180908	68935	1588988	1372285	2,09
653	391,45776	21,254	0,794	11	1,746	4,532	4,90E-01	1,471	3,486	5,80E-01	0	61111	336403	1243660	806875	10887723	62395	330913	4053260	235803	929893	1396498	405349	2523949	1340924	57821	3,24
655	692,53571	58,958	0,857	5	2,23	4,329	1,60E-01	2,904	5,128	8,00E-02	0	124780	1226559	1021253	1278068	34953	49359	962855	190983	43174	506218	455927	72110	108023	335808	63429	5
657	758,3009	29,119	0,853	3	1,33	3,507	6,90E-01	1,291	3,179	7,10E-01	0	35119	103883	119308	978324	1887794	379966	266061	2591614	178151	60930	169881	399916	1833154	478776	184778	2,88
659	745,69586	43,385	0,63	2	1,786	4,955	5,70E-01	2,259	6,412	4,90E-01	0	975075	67641	26472	18269	93847	22144	18159	19135	61540	59144	128783	53421	56165	17546	24536	2,96
660	478,76782	30,771	0,864	27	4,001	3,486	1,20E-03	4,067	2,844	3,40E-04	0	323804	499568	634355	1219015	1102111	440551	1211975	335838	128984	155713	241367	105301	231027	701392	432340	3,73
663	498,22708	42,498	0,794	5	1,271	2,805	7,00E-01	1,422	2,861	5,20E-01	0	58081	186026	228027	1602305	1064243	414962	439133	97354	198678	87606	196966	241621	1153487	565998	116831	2,71
664	715,34869	45,003	0	1	2,748	3,115	1,50E-02	2,999	2,783	3,20E-03	0	353283	1008425	857122	1323123	1444128	603313	3024708	1394078	361070	334126	732758	193911	505851	1256221	430138	2,92
665	566,63489	52,393	0,567	7	0,365	0,57	1,00E-01	0,431	0,558	8,00E-02	0	424565	1184553	809297	446855	510384	60910	1784616	438268	372866	3031905	2391849	856193	1521022	205900	100387	5,72
667	510,25397	40,923	0,653	3	3,477	3,788	1,20E-02	3,759	3,259	4,50E-03	0	321137	506185	630996	1549960	852085	576057	2481046	2245919	215230	252023	396716	220903	469465	1233722	412863	2,87
670	839,44672	47,318	0,532	5	4,797	19,284	3,80E-01	5,667	23,59	3,70E-01	1	1285893	17922	17659	13397	46155	26522	8632	18374	6506	19194	14464	103464	10846	18104	29455	2,1
671	521,80847	34,092	0,893	4	2,278	2,907	5,60E-02	2,625	3,253	4,10E-02	1	1064031	723239	1082816	176321	150105	318517	48935	1573518	330099	167508	598362	227102	507807	1859651	1346141	1,99
673	796,92291	45,937	0,781	11	3,462	7,306	2,50E-01	3,783	7,803	2,10E-01	0	30344	154913	73744	1108224	17285	356992	35682	48453	59816	63765	66553	27392	52639	27289	17741	2,17
674	632,07098	57,941	0,754	3	5,555	16,978	3,40E-01	6,131	17,634	3,30E-01	0	1127133	31162	16568	19212	100938	8500	24620	15252	9062	41427	30212	22067	19862	12220	6851	3,98
676	848,21954	22,497	0,975	3	4,863	8,385	2,00E-02	4,383	7,294	1,40E-02	0	152723	867114	744612	68677	194191	852306	1619243	1431279	18411	301937	126129	50119	159138	327762	119976	6,98
677	522,05017	44,606	0,85	20	0,3	0,276	3,80E-03	0,323	0,261	1,60E-03	0	161586	1060179	938543	512494	1122001	120661	534852	378782	1812464	1310463	2025271	1615199	2843891	596938	337609	4,98
678	551,47968	56,796	0,819	3	10,473	34,689	3,30E-01	11,633	36,618	3,30E-01	0	1169735	11410	10422	5701	19415	27339	39030	9114	10248	23843	10674	10305	7362	8125	7356	5,94
679	687,43317	58,787	0,74	10	1,513	2,419	3,50E-01	1,477	2,736	4,40E-01	0	9537	285101	1153619	1260162	1193076	8030	141951	1038239	704925	218342	75130	341058	669617	960081	31389	2
680	611,31915	43,384	0,725	37	1,086	2,266	8,90E-01	1,253	2,367	6,70E-01	0	183119	861048	822709	1299318	1556785	1657242	3935337	363052	252033	295812	915603	693270	3187484	558751	368784	2,34
682	656,34003	42,474	0,471	3	1,19	3,638	8,30E-01	1,516	4,713	6,40E-01	0	976395	78260	58329	67538	58923	69128	82153	85808	48864	436496	46709	87971	52015	54264	68791	3,23
683	757,39691	45,515	0,856	35	6,077	8,412	2,10E-02	6,638	7,523	1,00E-02	1	249112	185012	293456	982633	456964	491887	1815810	1870282	64491	106831	167970	66924	160565	410166	133034	2,09
684	413,88492	27,644	0,665	6	2,805	4,1	7,40E-02	3,01	4,579	8,70E-02	0	1155553	811104	583095	199884	179590	123078	79637	77937	245902	110997	387295	161976	212467	1143220	1922524	2,64
685	533,34106	58,284	0,877	28	1,437	2,596	4,70E-01	1,475	3,299	5,30E-01	0	578671	752783	388946	1210227	812518	1443594	243343	101986	1640925	239865	529822	434023	95904	2270589	647698	2,05
686	514,78027	49,788	0,785	38	0,876	1,25	7,30E-01	0,985	1,212	9,60E-01	0	76894	1250210	673578	572200	385787	130757	948996	1217623	270377	984915	978440	174668	770061	228361	84855	3,99
687	547,95978	60,587	0,765	7	1,832	3,816	3,60E-01	2,434	5,362	2,80E-01	0	21011	166267	1340776	427349	318651	55299	649710	2367527	65146	559125	128339	316621	413461	55941	28900	3,02
690	1208,23071	58,948	0,958	22	1,68	3,47	3,80E-01	1,575	2,698	3,50E-01	0	371417	2171380	43096	1272263	4543982	6977	3081525	494877	544619	846885	1805741	6072	601944	793050	5046	6,99
691	761,89539	54,108	0	1	2,566	7,146	4,30E-01	3,133	8,586	3,70E-01	1	875816	32606	28098	19220	37285	20928	32908	39539	26604	72916	29197	65141	32479	20203	54789	2,17
692	649,55304	45,991	0,812	3	0,178	0,226	1,30E-05	0,197	0,266	1,50E-03	0	73618	199894	983726	70190	257078	66339	119906	106745	1386425	1316478	1257452	1830816	1260849	359597	275306	4
694	1208,08716	59,063	0,932	8	1,687	3,488	3,70E-01	1,589	2,729	3,50E-01	0	354771	2182622	48358	1282240	4525455	5234	3026621	507637	549475	860796	1789860	4757	565101	788325	1446	7
696	494,59686	38,449	0,874	2	3,243	4,714	2,80E-02	3,83	5,651	3,00E-02	0	1073659	634362	1163324	442776	128433	424915	946224	228360	289736	118794	451219	94764	497517	2625876	1748718	2,94
698	891,87231	24,881	0,973	2	4,976	8,686	2,40E-02	4,696	7,853	1,60E-02	0	175949	684215	865942	91910	175384	777801	1486771	220046	8875	211948	143915	41026	87388	153907	275605	5,96

699	480,75974	29,866	0,776	3	0,908	1,062	7,60E-01	0,888	0,958	6,80E-01	0	118744	514653	731682	1233842	572645	403564	813007	1807025	257629	1182315	899413	668155	829169	618382	154059	3,7
700	999,83307	57,062	0,895	37	0,429	0,643	1,50E-01	0,471	0,617	1,10E-01	1	40937	1081016	1077732	543107	955273	78004	1226049	176716	762699	1818110	2588950	1549041	100150	500955	175734	3,03
702	558,28882	23,555	0,842	36	1,07	1,277	8,50E-01	1,072	1,161	8,20E-01	0	228909	523834	1062826	359449	458220	377917	505965	242589	312650	207064	595199	281456	1026847	811990	615951	3,88
703	891,70471	24,881	0,977	2	5,143	8,983	2,40E-02	4,857	8,096	1,50E-02	0	208258	689245	915968	98865	183057	802932	1590796	232886	9059	213382	147494	40768	91226	159601	281479	5,93
705	523,62457	35,71	0,877	6	2,117	4,628	3,30E-01	2,491	5,524	3,00E-01	0	1016188	70729	53269	300318	171110	50443	64450	27292	53562	65341	81740	63087	189108	80587	82665	2,97
706	855,36267	21,013	0,967	16	2,803	2,612	3,00E-03	2,572	2,186	4,20E-03	0	145571	439073	848660	403218	342442	807350	502894	321769	60973	158889	176901	145657	252559	291349	353568	3,95
707	508,74173	39,923	0	1	3,614	5,01	4,90E-02	3,788	4,943	2,90E-02	0	157815	223429	190404	1251026	222206	241984	345440	241531	66909	50821	101576	141377	92811	241667	162730	2,3
709	549,24438	19,664	0,746	10	1,898	2,582	9,90E-02	1,691	1,907	1,20E-01	0	258145	428044	485921	597770	1129661	844540	1099721	1604437	161628	232357	427616	237887	1043497	847010	689250	4,65
710	586,3266	49,562	0,652	33	2,051	3,181	9,90E-02	2,524	3,44	1,90E-02	0	227624	550967	1027068	1570467	269844	1025706	1179320	1402644	246205	1143140	249431	100501	359917	1032626	323147	3,06
711	615,8335	21,631	0,723	37	0,058	0,069	3,70E-03	0,053	0,064	3,50E-03	1	78235	633542	508409	115866	130387	316497	96454	151836	3846669	1698300	4113813	5295839	4809340	111372	130832	1,98
712	557,80774	53,79	0	1	1,45	2,658	4,60E-01	1,876	3,168	1,90E-01	0	92101	954972	354662	1311941	172143	777616	270102	734140	90222	428102	832326	70528	248092	92809	79175	3,97
713	734,37103	47,847	0,777	23	4,839	5,955	3,10E-02	5,065	5,038	1,70E-02	0	136268	294095	529834	833421	211451	693785	1669971	2051926	113241	171281	143528	98550	179317	309507	101778	3,97
715	849,37952	55,105	0	9	1,148	3,368	8,60E-01	1,547	4,158	5,60E-01	-1	27705	690710	299188	3435662	238378	1272089	281051	98507	262765	496687	1925438	67483	117348	142606	102052	3,92
716	628,35455	39,587	0,754	2	2,267	4,946	3,40E-01	2,808	6,334	3,10E-01	0	1089157	98974	77339	152665	48440	155100	73057	79283	52908	97116	127336	38134	90945	49706	19411	2,34
717	849,71649	54,502	0,956	2	1,242	3,139	7,50E-01	1,635	3,699	4,40E-01	0	22271	325382	244702	1596974	121469	582984	143119	166658	140498	272492	791143	32725	115199	75976	78911	3,06
718	777,39252	56,565	0,815	3	1,905	3,822	4,40E-01	2,054	4,289	4,00E-01	0	1072264	89376	70431	71737	71759	51864	89662	88680	73751	95746	89524	105669	81445	54551	39743	3,09
720	532,91364	51,661	0	1	5,558	8,079	1,30E-02	6,784	8,856	6,30E-03	0	179680	337206	255183	1341470	362103	262039	531053	322235	54633	56548	132090	17793	107123	290308	211222	3,03
721	555,35406	59,007	0,837	18	1,612	2,871	3,40E-01	1,681	3,744	4,00E-01	0	620153	912535	745406	1362482	909007	1662177	303284	110395	1739470	262328	578848	463093	25711	2485396	786418	2,01
722	394,72955	25,339	0,993	3	3,312	5,1	1,10E-01	3,401	5,181	1,20E-01	0	148928	201027	108957	1218581	129479	1352952	118156	143455	85213	143227	149800	78515	118541	177718	211611	3,33
723	543,94739	36,639	0,8	18	2,496	2,643	2,70E-02	2,628	2,729	2,40E-02	0	103839	411935	1096538	526614	105984	818719	676824	388673	193259	160250	168587	113081	288135	335721	144448	3,06
726	491,25269	34,777	0,884	8	0,265	0,43	1,10E-01	0,272	0,412	1,00E-01	0	279885	1682021	1164403	1184622	1708745	730222	677243	3851817	2256768	10968011	1432034	3584354	5438946	757033	522836	2,93
727	787,2016	59,474	0	1	1,391	5,657	7,70E-01	1,475	5,836	7,20E-01	0	35691	1193064	24898	20085	4880565	6671	409380	63470	13558	301134	1986028	22834	71501	20703	8515	13,9
728	566,27356	38,02	0	1	2,167	1,868	6,20E-03	2,371	1,762	1,80E-03	0	329056	526888	586390	1152521	400636	506664	544759	247894	199659	153680	324978	189707	406286	662002	565282	3,63
729	630,26892	36,922	0,826	18	2,912	3,133	2,70E-02	3,11	3,443	2,40E-02	1	62360	534707	958288	182658	101314	808699	932750	95716	201761	147359	244377	114703	155554	1011121	342373	3,97
730	642,14673	42,221	0,954	9	0,285	0,832	3,20E-01	0,311	0,9	3,30E-01	0	10223	946800	70478	587405	49235	14375	243434	77854	74658	950371	2381825	14120	186506	47400	6706	5,99
733	478,51718	30,771	0,811	11	4,411	4,366	1,30E-03	4,507	3,682	3,60E-04	0	269906	436372	562611	1111695	1028943	401337	1097707	296320	99299	101734	214348	83368	207232	640243	383311	3,97
735	576,81403	58,981	0,954	5	3,782	7,64	3,20E-02	4,734	8,558	1,60E-02	1	504440	699384	151796	1285185	208458	229148	212769	28439	125820	57196	331467	34462	10593	441061	471912	2,71
736	1046,52893	68,328	0,686	7	0,546	1,701	5,40E-01	0,564	1,615	5,30E-01	0	73411	1673109	18459	138978	208427	10589	1489905	45493	63081	1063234	2232860	32167	46759	86081	10574	8,85
737	742,87341	56,893	0,954	5	3,107	8,168	3,60E-01	3,426	8,687	3,40E-01	0	947899	50220	38153	37233	33467	29032	50972	30885	24162	62508	34152	54389	31924	37600	31645	4,53
738	422,76859	42,889	0,875	2	6,333	20,429	3,20E-01	8,018	26,84	3,20E-01	0	1316762	11494	17652	29162	69804	27855	36619	64672	9449	41028	14267	47426	14532	24467	6330	1,96
739	566,80164	52,393	0,65	15	0,352	0,581	9,90E-02	0,412	0,577	7,90E-02	0	83230	1136551	851864	466628	487728	75292	1768919	431123	348377	2967001	2365306	813373	1487839	207816	105392	5,72
740	706,90112	38,467	0,867	5	4,991	5,202	8,40E-03	5,081	4,931	4,50E-03	1	69451	246744	317900	913861	193051	415531	747981	374611	79741	69411	77317	39611	97165	252632	94219	1,98
741	545,60315	38,299	0,747	33	5,082	4,471	1,30E-03	5,365	4,49	9,90E-04	0	122825	351128	922188	581538	188857	513936	796707	801908	109608	75375	150481	69543	127098	881666	247495	3
742	764,46393	24,969	0,979	3	5,587	10,003	3,10E-02	5,211	8,789	2,00E-02	0	171384	600206	868983	90520	175287	696722	1664555	215466	11430	184395	119074	39490	82116	147723	246423	6,67
746	753,47284	68,526	0,769	26	1,43	2,487	4,80E-01	1,446	3,389	5,80E-01	0	624094	526395	299241	1163556	349494	1017740	572558	152955	1420201	251928	344390	288960	115268	1612752	603526	2,58
747	652,3631	53,118	0,891	4	1,557	3,46	4,90E-01	1,95	4,182	3,40E-01	0	111785	154993	1363536	340400	162080	124182	142547	88668	113688	422200	81835	64214	212206	222995	72339	8,82
748	587,58154	48,757	0,778	4	4,205	12,847	3,90E-01	5,158	16,69	3,80E-01	0	1089164	24284	20461	12849	23545	13297	27188	21529	40764	43123	24875	17214	24209	14024	16614	4
749	717,93121	25,917	0,492	2	1,161	2,264	7,80E-01	1,102	2,476	8,70E-01	0	839867	148965	128100	103540	109936	123564	73598	317978	387319	104644	142396	86753	124540	108955	9932	10,9
751	560,94696	54,305	0	1	5,27	8,006	9,30E-03	6,366	9,286	1,20E-02	0	108886	457474	362342	1027575	411740	284637	868303	252499	114976	64261	266087	51105	87136	1699228	677828	3,14
752	447,73944	40,568	0,678	4	0,78	1,628	6,40E-01	0,956	2,104	9,40E-01	0	1199791	212418	165889	78817	51194	80297	77324	81252	294730	155462	320101	156326	430651	96424	74999	2,16
753	445,54065	28,392	0	1	0,532	1,388	5,30E-01	0,496	1,333	5,20E-01	0	0	354190	999	285068	0	150904	0	48842	893485	10969	185977	103617	46611	357905	122510	2,89
754	485,91266	26,544	0,736	19	2,728	8,287	4,00E-01	2,153	5,935	4,30E-01	1	127057	381733	1055858	834637	17359748	76184	286257	2231597	375101	464496	1254569	316009	1983161	1476124	141420	2,98
755	1046,64014	68,33	0,683	8	0,539	1,72	5,40E-01	0,55	1,647	5,20E-01	0	28363	1634395	11236	123221	185123	4588	1463046	28205	55198	1052126	2133319	6785	53944	78833	3171	9
756	548,29395	60,527	0,918	4	1,622	3,146	4,20E-01	2,122	4,423	3,10E-01	0	65301	171984	1262474	414292	313656	69895	628679	2240199	89559	557427	162646	376869	444171	67123	55367	3,15
757	689,1239	57,109	0,759	5	3,274	5,673	6,50E-02	3,738	5,749	3,50E-02	0	100855	227864	198422	1101398	69904	308151	506040	144894	107604	61789	198978	53143	37594	177346	171641	4,53
761	726,65436	38,664	0,811	45	4,011	4,318	8,60E-03	4,174	3,805	3,50E-03	1	94027	155800	188752	728398	307277	236339	674486	268624	65468	77079	114155	38429	93156	344537	116556	2,88
762	551,64648	56,796	0	1	1,506	3,566	5,40E-01	1,869	4,072	3,70E-01	0	1106204	208264	129770	131344	60932	77134	285129	76997	59459	262806	312430	82484	12751	93897	28994	3,3
763	712,99591	24,125	0,871	14	0,723	1,264	4,50E-01	0,734	1,338	5,00E-01	0	25426	217783	161941	989973	131993	164894	83449	154612	163109	283822	426618	207909	334848	89915	28021	2,7
765	637,85284	51,98	0,835	3	0,459	0,453	8,50E-03	0,546	0,436	5,90E-03	1	48926	156666	75991	40541	128222	13438	54468	19841	91330	142579	122087	145463	199290	47148	58739	3,86
769	927,48743	67,931	0,803	3	0,498	1,159	3,40E-01	0,622	1,098	3,90E-01	0	254132	401247	243760	1229161	33747	199263	3025000	91202	177245	2299441	3211679	1160354	64050	1173718	237313	5,89
771	804,9082	51,262	0	1	5,625	7,426	4,70E-02	6,462	7,953	3,60E-02	1	113153	810285	941775	259941	193099	68459	58902	71959	43752	60252	60854	39222	52554	344416	25019	2,07
772	538,00659	26,861	0,818	18	2,395	4,517	1,10E-01	2,432	4,493	1,10E-01	0	389987	377526	98622	846585	116156	148576	85283	37838	119097	42282	279942	28173	67654	274740	197115	3,91
773	766,85632	61,999	0	1	0,809	2,069	7,70E-01	0,792	1,697	6,90E-01	0	125976	1232455	60230	282914	1975731	22924	2452652	95903	362315	995358	2253123	67343	265203	109134	21966	11,47
774	978,03461	38,487	0,882	79	2,273	2,621	3,80E-02	2,365	2,739	2,40E-02	1	152561	527876	625197	1046748	101027	418710	376355	152035	253662	167828	211498	46095	222490	500231	197541	2,04
779	731,45953	67,963	0,744	30	1,383	2,714	5,60E-01	1,395	3,499	6,40E-01	0	655490	589993	180966	1225090	130203	1128486	550695	80715	1557506	215851	359769	310838	85239	1801895	653688	2,03
781	572,32068	47,329	0,816	36	3,226	2,941	9,80E-03	3,456	3,201	6,20E-03	1	147901	271793	483796	1105827	147566	524422	622861	487272	149165	123895	117102	106112	189516	505497	127911	2,62
783	531,29803	42,89	0,76	22	1,788	1,838	5,40E-02	2,027	1,77	1,20E-02	0	286719	629920	997508	1343781	528068	475462	669936	1105239	162214	641650	397530	240315	542248	887461	170432	2,57
785	460,59079	45,066	0,805	12	5,305	7,144	1,50E-02	6,049	6,626	5,00E-03	0	1268197	612767	1087889	765729	1851872	437463	4095344	397300	138106	248561	414263	128494	259660	1045566	1054650	3,74
787	522,80682	27,658	0,805	29	2,197	5,114	2,10E-01	1,895	4,183	3,10E-01	1	479359	620067	462940	931714	3047865	868323	2344929	903355	201757	76919	175805	1937292	166188	898928	680901	2
790	769,33466	59,01	0	1	1,434	2,763	5,00E-01	1,334	2,218	5,30E-01	0	492101	2355718	155707	1541762	6022848	22536	4112055	1154758	639813	1229388	2206797	27484	1726640	840215	28171	10,98
791	500,23575	52,286	0,944	4	1,31	2,993	6,80E-01	1,596	4,005	5,20E-01	0	1139065	152198	406098	40021	46861	25767	30110	22393	327983	209081	32019	70026	206795	34807	319440	4,55
792	629,85681	38,605	0,718	13	1,211	3,784	8,30E-01	1,279	4,088	7,80E-01	0	100321	56525	126866	918858	46243	249056	166479	184037	57516	129303	28640	1271241	87133	1747702	214195	2,45
794	1208,37439	58,978	0,971	3	1,695	3,491	3,70E-01	1,593	2,729	3,40E-01	0	303178	1813121	35778	1096540	3735858	3729	2479857	403767	465451	685994	1488794	4368	472948	694590	1513	7
796	635,6543	31,949	0,751	24	1,168	2,236	7,70E-01	1,294	2,457	6,20E-01	0	244368	2111920	1128007	1856380	327247	2028681	255825	601750	81093	861380	1919659	110067	822598	239326	70962	3,05
797	541,02142	25,087	0,822	20	0,84	1,412	7,20E-01	0,834	1,396	7,00E-01	0	81441	750401	366397	656986	281423	667012	63221	119881	355654	178401	869663	93241	383454	96129	75901	3,98
798	917,94934	39,114	0,733	53	0,254	0,243	9,90E-05	0,267	0,216	5,10E-07	1	154097	1023443	563210	280545	904900	57694	375846	119155	1272135	1495898	2120653	1668369	1839374	490861	293726	2
799	689,88013	51,273	0,778	3	0,248	0,868	3,70E-01	0,322	1,074	3,90E-01	1	60140	78557	2284056	42598	20930	181736	42870	2599487	17423	8655407	20139	1894468	453842	136372	36002	5,15
801	748,74359	59,837	0,703	5	4,011	11,701	3,10E-01	3,96	11,846	3,00E-01	0	1173702	32365	22323	18746	95972	17437	151497	19160	16907	41141	32406	89412	22610	26072	67005	3
807	709,72766	51,341	0,773	43	6,683	14,146	1,10E-01	7,743	15,068	8,30E-02	1	456421	565306	698067	876166	1701793	1538377	2526489	9776930	236192	185266	421942	77112	497135	390042	417395	3,07
808	641,30957	44,781	0,753	27	0,34	0,323	6,90E-03	0,36	0,397	4,50E-02	0	129229	202307	1009583	235233	832166	212757	993330	567412	2496183	1040447	1415707	1560954	1723602	866264	549275	3,89
811	637,37775	42,025	0	1	13,976	52,032	3,30E-01	18,582	69,152	3,30E-01	0	938001	4291	6848	9476	34608	3682	8403	10303	3072	16519	11186	2021	3860	5139	3918	2,18
812	635,83606	40,621	0,966	2	7,258	10,285	2,40E-02	8,27	11,269	2,70E-02	0	1300917	165420	218623	353184	130728	207203	1037924	1076863	32323	82380	85908	39155	83976	170349	38196	3,61
813	698,11023	45,094	0,935	10	2,069	3,526	1,70E-01	2,414	4,081	1,20E-01	0	33422	322211	303201	799685	249177	19531	49123	418033	39856	245044	195635	30570	223061	822830	21080	5,73
815	834,77472	71,281	0,901	2	3,535	10,463	3,60E-01	3,781	9,828	3,40E-01	0	1067748	48213	15030	27391	83325	10017	66578	18760	16739	66883	61842	29252	21344	28715	20536	3,14
817	463,30737	37,829	0	1	15,507	71,12	3,70E-01	20,244	93,443	3,60E-01	0	1000328	604	458	1597	1624	546	651	1372	260	22678	7141	586	1862	502	1106	2
819	854,38934	42,221	0,886	39	1,652	2,824	3,80E-01	1,714	3,197	3,50E-01	0	66926	308194	929024	96147	90964	141610	79386	124318	209253	108453	94139	90663	111109	48255	143053	2
823	732,05902	57,745	0,799	19	1,291	2,397	6,20E-01	1,706	2,638	2,20E-01	0	246101	508141	170666	941024	153287	103472	151723	62417	101541	369091	530467	62815	58747	331157	231170	3,01
827	977,53363	38,523	0,91	15	2,155	2,639	6,20E-02	2,235	2,767	4,20E-02	1	133790	487473	598178	950270	65995	337504	277238	114323	244686	149191	189353	42138	205517	437501	178221	2,01
828	599,34033	53,679	0,969	2	3,174	7,277	2,90E-01	3,927	8,667	2,60E-01	0	1085799	97192	70359	85460	43262	53004	68025	71792	45534	72079	78039	34926	35719	36080	79338	2,97
830	588,60614	52,512	0,774	8	6,361	7,386	5,80E-03	7,216	7,854	2,40E-03	1	102277	323280	392963	1084348	354172	352151	1042214	885740	80435	73519	98978	26131	111468	347522	83326	3,04
831	971,02484	52,793	0,9	3	9,104	29,224	3,50E-01	11,936	37,325	3,40E-01	1	791939	5994	4433	10679	14216	8532	10323	5872	6993	12765	8645	6111	12865	7596	3109	2,07
832	497,26675	33,335	0	1	1,871	1,699	4,80E-02	1,965	1,602	2,40E-02	0	366148	541160	334595	1237535	268003	296917	590647	384776	181713	167006	310135	316844	270313	355031	287635	2,67
833	540,77094	25,12	0,721	8	0,984	1,172	9,60E-01	1	1,217	1,00E+00	0	244731	722051	480276	685757	300965	656675	208995	169575	579036	268567	1051262	210145	573278	1108946	698216	3,26
834	539,76843	21,013	0,816	9	0,898	1,389	7,90E-01	0,797	1,204	5,60E-01	0	37719	87607	701692	266451	100664	270499	77957	629729	117932	217923	175555	464119	339166	57081	130606	2,91
835	409,52899	30,814	0,658	2	1,901	2,371	9,80E-02	1,843	2,308	9,80E-02	0	145627	240193	381760	1196582	1012268	300779	350700	532487	404686	159052	211171	116067	345514	294197	246046	2,84
836	588,94037	52,463	0,858	8	6,184	7,012	6,50E-03	7,055	7,364	2,70E-03	1	89470	296771	379694	1032725	340334	329240	997895	822118	74714	94176	87503	26404	95784	324967	68808	3
839	709,44641	59,49	0,796	14	1,735	2,478	2,10E-01	1,751	3,007	2,60E-01	0	13022	253419	1120406	1161867	1135352	4135	246617	985786	457679	208067	117757	302829	519071	638060	11793	2,39
841	635,98798	31,852	0,789	22	1,168	2,255	7,70E-01	1,291	2,471	6,30E-01	0	151576	2121899	1168506	1915346	341492	2048629	278255	612881	83493	859814	1943809	105576	839364	243367	73249	3
844	408,21945	22,866	0,792	7	1,781	1,558	2,70E-02	1,754	1,365	1,90E-02	0	1091418	537766	1232656	992681	248393	1030760	1738930	1143995	508595	450976	730442	329151	1051073	1662050	1255704	2,47

845	927,65515	68,052	0,802	12	0,498	1,15	3,30E-01	0,615	1,089	3,70E-01	0	258185	473997	291682	1566413	65509	235472	3629268	88018	208217	2767035	3716689	1520938	72081	1356131	288075	5,99
846	712,66187	24,125	0,899	2	0,723	1,21	4,30E-01	0,732	1,276	4,70E-01	0	22582	207427	154692	909627	136166	166618	78909	147065	160705	271127	391392	199817	320599	89260	31844	2,77
847	425,71906	25,087	0,642	2	2,192	1,986	1,70E-02	2,112	1,898	2,90E-02	0	192678	230005	461088	1064706	426933	348145	371178	634854	199589	124134	225631	165672	301050	224747	499885	3,33
848	438,9187	40,621	0,767	28	2,539	1,891	1,50E-03	2,816	1,622	2,80E-04	0	1212498	703359	684195	899274	926571	584224	1747935	1271144	272381	270655	500722	294864	474146	770893	403743	2,76
849	643,841	32,893	0,788	43	1,108	2,579	8,70E-01	1,194	2,615	7,60E-01	0	194576	263156	258645	1696596	193607	335346	95174	142830	156400	219840	750848	59120	331923	78835	104914	1,99
851	797,42401	45,937	0	1	2,796	5,697	2,50E-01	3,178	5,885	1,90E-01	0	44718	122338	59922	819799	14004	282129	31210	42481	41960	54289	87528	33696	50798	24691	58827	2,34
852	764,60638	24,881	0,968	2	5,424	9,68	3,20E-02	5,075	8,547	2,00E-02	0	152136	564514	800991	90240	155710	643891	1527226	192288	11268	172539	117082	35279	78633	133956	239155	6,75
854	613,79572	46,83	0	1	2,22	4,368	3,60E-01	2,584	5,846	3,50E-01	0	951847	84238	50127	171519	63493	35287	72012	56153	83685	69624	73230	53141	71684	42965	32433	2,09
856	537,75647	26,961	0,803	8	2,076	2,843	8,10E-02	2,117	2,866	8,30E-02	0	392289	435025	146148	805921	127902	187604	116273	56729	149584	95523	297914	69996	119399	284120	489398	3,77
858	787,12933	59,468	0	1	1,384	5,595	7,70E-01	1,468	5,73	7,20E-01	0	44489	1229891	40419	34377	5184486	17402	425393	65244	22706	321956	2101135	27371	83233	27842	8252	13,87
859	590,3374	51,258	0,841	15	5,51	5,223	2,60E-03	6,617	5,413	1,20E-03	1	140607	338276	1013363	778526	316994	524985	716424	1420395	93547	99658	129938	78501	170312	809918	243299	3,11
860	515,30133	40,509	0,915	15	2,747	2,541	3,60E-03	3,152	2,605	1,50E-03	1	1247240	514198	1492811	1454216	768448	1999892	2348586	3514361	553418	628305	716192	258833	970989	2412255	1432768	2,5
861	517,26215	25,196	0,818	10	0,485	0,782	2,60E-01	0,448	0,743	2,60E-01	1	210233	197320	893013	509528	121590	210261	159210	149664	1418627	1072640	152026	398165	221501	296356	419473	2,48
862	558,03802	23,625	0,905	2	1,016	1,443	9,70E-01	1,026	1,343	9,40E-01	0	128283	368541	837287	210415	325345	222078	255949	87172	219971	121917	430592	178113	760762	593169	447593	3,98
863	391,70856	21,325	0	1	1,753	4,472	4,80E-01	1,48	3,438	5,70E-01	0	78059	317501	1042425	736921	9348575	79077	321401	3455275	236440	842277	1216077	313802	2135798	1177692	73271	3,68
866	544,21893	20,78	0,818	3	1,255	2,344	6,50E-01	1,216	2,272	7,00E-01	0	28958	89099	113214	632520	130564	249833	39375	103288	77865	49338	63858	163411	283208	110377	103065	2,97
867	514,53027	49,813	0	1	0,897	1,322	7,90E-01	1,009	1,305	9,80E-01	0	51985	987362	529190	426630	302759	84197	729014	942093	208938	745599	739702	93461	605056	179579	59417	3,96
869	918,49396	57,084	0,783	15	3,692	6,247	7,60E-02	4,266	7,129	4,60E-02	1	40980	132826	155323	798814	130286	208007	102127	122320	71112	66652	78822	21398	20393	121586	95598	4,02
870	820,24249	44,501	0,95	17	0,484	0,752	1,80E-01	0,521	0,746	1,70E-01	0	19978	911958	370571	879793	370401	63210	489461	456913	406794	721578	1606919	205306	968068	195964	22782	6,92
872	758,45795	21,74	0,994	2	4,831	9,451	4,80E-02	4,234	7,904	3,70E-02	0	123450	639390	476331	35629	88503	487138	1643547	669809	9451	218233	101734	35821	97269	242110	62446	7,26
874	775,48596	69,267	0,714	32	1,513	2,573	4,00E-01	1,49	3,451	5,40E-01	0	570824	447657	171490	973576	776934	886372	578631	82358	1236861	222199	335043	255505	62355	1370881	532599	2,1
876	435,77393	27,998	0,64	29	1,105	0,978	7,10E-01	1,039	0,901	8,90E-01	0	778555	1099488	913633	1851180	1175597	1248365	2344053	1547308	1505208	2270978	936419	659715	845034	1767593	1019670	1,96
877	742,86456	48,756	0,442	2	3,119	4,452	7,40E-02	3,427	4,948	5,30E-02	1	119920	200186	147621	890768	144327	95682	110815	81370	94712	57947	67897	45555	115042	439594	147681	3,02
878	419,90018	43,512	0,911	2	10,567	34,342	3,30E-01	13,71	44,665	3,30E-01	0	1296893	11042	11486	17123	31184	14335	22485	18297	10559	23739	5902	15212	13614	19183	16687	2,82
881	653,3009	45,953	0,899	11	0,412	0,476	4,80E-02	0,429	0,561	1,30E-01	0	82067	554767	913124	275585	647703	153447	196403	242735	1601118	434170	806090	715131	1140472	529759	271479	3,49
883	681,53088	55,272	0,888	6	1,324	2,575	6,00E-01	1,617	3,055	3,70E-01	0	38190	413297	918614	1084556	1001726	153929	1190772	2155416	169251	1508049	445068	263498	337426	197800	58651	5,02
884	454,23831	34,279	0,83	40	1,47	1,607	2,30E-01	1,706	1,816	1,30E-01	0	874083	466523	1080897	256655	248387	412404	223825	606035	356702	326430	603321	340454	732885	1734102	1035140	2,8
885	516,27686	36,836	0,875	6	2,816	2,129	1,50E-03	2,948	1,928	3,30E-04	1	801940	529460	505446	1800358	1065014	774420	1412434	663613	320118	190798	344251	293692	454260	853507	621890	2,03
886	822,67303	43,155	0	1	0,327	0,529	1,20E-01	0,348	0,526	9,90E-02	0	26293	645074	241100	602828	217685	47018	376412	250517	544609	611688	1739402	157636	901271	171670	7221	6,74
887	822,81549	43,31	0,915	30	0,316	0,53	1,20E-01	0,335	0,53	1,10E-01	0	23304	792304	284912	692357	255880	36098	431637	284734	645093	711760	2156468	180901	1068651	198689	5555	6,79
892	472,25714	23,046	0,864	3	1,071	1,566	8,70E-01	1,127	1,918	8,10E-01	0	973860	187061	219588	102974	109883	105884	122477	81428	249804	171072	220200	135507	256953	168604	143007	3,38
893	599,76636	21,359	0,766	14	1,193	2,305	7,30E-01	1,104	2,353	8,60E-01	0	89093	144628	222311	702090	28081	203870	72094	56694	350834	69922	118714	46933	151015	76416	164503	2,25
894	590,67126	51,258	0,865	17	6,304	6,666	2,80E-03	7,644	6,958	1,40E-03	1	94229	319259	969977	702393	272548	476742	685663	1315581	73244	90983	101584	47340	146956	761194	203693	3
898	731,45917	60,261	0,796	13	2,496	4,313	9,30E-02	2,352	4,563	1,50E-01	0	10094	123750	1020924	1007996	1041441	4784	297872	878858	329556	23868	31344	284033	303529	459469	8614	2
899	544,31671	44,697	0	1	1,916	3,914	4,00E-01	2,337	5,171	3,60E-01	0	786557	56280	61569	33711	44834	48322	102389	111756	49365	59834	91588	98364	51818	52381	47412	3,41
901	545,93701	38,299	0,852	9	4,738	3,86	1,70E-03	4,974	3,862	1,20E-03	0	98469	288393	761934	473764	160663	405716	688393	673064	95223	75277	125894	68811	106229	723684	193006	2,99
902	997,28253	68,349	0,726	6	0,54	1,636	5,10E-01	0,56	1,521	4,80E-01	0	33908	1604986	13391	211808	142445	11558	948051	42795	107063	941859	1724221	17949	80203	80721	9314	9
903	664,60889	61,681	0,991	2	0,947	2,485	9,40E-01	0,962	2,156	9,50E-01	0	99790	867007	26658	132837	907677	20673	1738620	76048	47837	672773	1125852	22340	213006	62205	12485	15
904	521,72845	51,256	0,859	18	0,984	1,304	9,60E-01	1,169	1,314	6,10E-01	0	103924	520604	1139703	778443	315924	570011	1630065	1291123	443062	604138	1120001	239086	1018546	195460	197163	3,97
906	782,65393	55,275	0	1	0,303	0,44	5,50E-02	0,318	0,403	3,80E-02	1	58647	1023412	809546	334827	843879	54014	1049038	165371	564741	1981439	2521442	2157215	595131	282995	124734	4
907	419,53787	22,429	0,961	2	1,305	2,328	5,80E-01	1,324	2,304	5,60E-01	0	70073	138822	443299	876869	162892	138960	108755	45851	186994	71456	120994	122715	420939	205945	218190	2,73
910	917,44836	39,22	0,748	33	0,257	0,241	5,90E-05	0,27	0,216	4,20E-07	1	153716	1016709	542610	286678	882528	57667	378901	127639	1258979	1463965	2042941	1655135	1776391	482385	290830	2
911	997,17096	68,345	0,743	5	0,541	1,629	5,00E-01	0,559	1,504	4,70E-01	0	46946	1673482	13864	213100	156997	14677	983827	48941	115707	983113	1793260	31869	74117	82762	8000	8,94
912	1000,1662	57,173	0,883	18	0,434	0,635	1,40E-01	0,471	0,595	9,90E-02	1	37936	957278	859698	496866	821788	67151	1027757	138666	674296	1497945	2154281	1338185	99012	421164	168925	3,06
913	544,26855	42,025	0	1	0,532	0,558	1,70E-02	0,585	0,659	6,10E-02	0	1167055	1370316	518732	387798	482496	124722	223665	139327	889244	768999	808642	1278083	1069115	457078	255296	2,69
916	690,38055	58,163	0	1	0,324	0,818	3,80E-01	0,473	1,163	4,50E-01	0	91670	1225560	1635322	1198026	65735	229798	102405	811458	138006	6651826	482718	1662203	139365	437733	85998	5,6
918	538,81219	51,259	0,634	3	0,997	1,599	9,90E-01	1,253	1,752	5,50E-01	0	89992	310568	939822	637216	579060	279032	500709	1696376	133567	954389	365685	295524	973743	246034	151033	2,07
921	539,2937	29,773	0,93	2	1,05	3,142	9,50E-01	1,098	3,479	9,10E-01	0	882351	56524	71357	63724	97165	44385	80583	95551	58164	94711	39859	478940	51828	59405	68729	2,93
925	488,27951	35,401	0,83	3	1,617	1,936	2,20E-01	1,713	2,453	2,40E-01	0	1064853	204743	204269	591082	265637	182824	169652	285637	303122	237442	158254	148221	172312	197352	131342	2,58
927	681,88635	73,762	0,964	2	7,458	25,418	3,40E-01	8,287	24,932	3,20E-01	1	927782	17571	7912	16109	25853	4905	26574	10992	8715	32113	13994	7722	9197	12456	19937	2,02
928	743,38086	42,596	0,776	23	3,468	3,67	1,90E-02	3,868	3,287	8,10E-03	1	183542	151060	156465	765628	138069	208974	291111	200380	47177	77898	93054	52974	77072	170608	149456	2,11
929	560,80499	21,655	0,821	10	2,254	3,77	2,10E-01	2,351	4,366	2,60E-01	0	541900	58325	84330	106966	83914	88556	28828	53868	37468	81420	45418	32670	63233	54886	71294	2,38
931	738,71014	48,277	0,751	6	2,189	1,592	3,00E-02	2,281	1,859	1,30E-02	0	150168	284906	373323	846824	203947	468138	812023	1073414	227553	219491	234539	216695	199940	419725	176177	3
932	533,26483	22,678	0,819	17	0,622	1,337	4,80E-01	0,556	1,231	4,40E-01	0	159005	99406	801468	224933	55480	114664	78080	75585	843506	307791	71785	190057	138662	100069	223088	3,4
935	602,98419	51,26	0,869	12	3,904	12,16	3,30E-01	4,501	13,245	2,90E-01	0	52110	269376	923140	793599	17083003	82706	367279	2711452	235514	359788	860479	149418	1382185	979226	65164	3
937	468,25317	30,877	0,702	2	1,645	1,982	2,00E-01	1,775	2,314	1,90E-01	0	1274547	351546	208073	525711	212300	182547	312115	175706	173165	146666	311107	338492	211114	313995	326450	2,87
938	425,58023	51,723	0,826	73	0,366	0,398	1,00E-02	0,417	0,452	2,50E-02	1	76883	1007661	773319	438965	714621	35795	324375	142812	1331498	1164389	827980	874306	1873862	647845	281995	2,89
939	681,7312	55,155	0,777	5	1,32	2,588	6,00E-01	1,614	3,107	3,80E-01	0	47910	409180	879158	1069152	966142	151827	1134116	2351696	167613	1495763	419645	264702	408086	210262	56448	4,93
944	652,31134	44,654	0,932	3	0,409	0,336	1,40E-02	0,457	0,349	8,20E-03	0	169969	810917	627140	549076	1001600	430074	554972	589246	1424912	912543	2059284	1207011	2108895	1198193	374422	3,53
945	882,51794	26,341	0,977	5	3,569	2,873	3,00E-04	3,319	2,257	1,70E-04	0	178318	587592	366235	265616	353478	298630	461919	189865	68919	74749	139412	53554	110734	322019	170064	6,95
947	603,31891	51,26	0,831	10	3,883	11,693	3,20E-01	4,47	12,635	2,80E-01	0	188932	317555	947992	857667	17361596	125968	437176	2793930	259688	406247	877835	181940	1382218	1000417	103606	3
949	555,35382	51,293	0,744	3	4,292	18,401	3,00E-01	5,825	24,967	2,50E-01	0	2692	2190	977506	2246	2438	2241	4284	862508	407	214253	1264	303	558	3337	1479	2
951	540,94995	55,415	0,871	5	4,534	4,23	2,60E-03	5,091	5,515	2,50E-03	0	116927	379452	611752	1020630	198279	619748	750150	437131	122438	95770	118419	49159	138617	476597	144828	3
952	521,47815	51,256	0,842	8	1,019	1,369	9,60E-01	1,209	1,388	5,40E-01	0	108876	467245	983583	702809	255348	501695	1450166	1188562	399075	528907	973238	174992	868350	162777	179500	3,99
954	741,32227	20,819	0,96	6	3,319	3,125	2,10E-03	2,971	2,641	2,30E-03	0	115064	284150	542395	139556	179347	387156	413077	217458	33323	102175	112452	56784	96151	170657	212101	4,3
958	547,27625	24,429	0,737	2	1,911	2,214	1,50E-01	1,991	2,794	2,20E-01	0	1169805	291669	254018	404294	232923	165871	184428	158312	176385	178538	208473	119759	155646	217644	127600	2,87
959	552,35114	30,719	0,699	4	8,413	29,477	3,70E-01	9,969	35,072	3,70E-01	0	648161	3902	4739	5097	6050	3102	3727	4185	4785	14812	7662	4039	9408	5175	784	2
960	854,89014	42,322	0,897	15	1,994	3,945	3,00E-01	2,014	4,325	2,90E-01	0	51483	252801	793262	69295	81010	113793	63247	92499	175099	69965	53616	58028	62306	36849	116494	2,08
964	872,41357	67,991	0,761	20	1,888	4,756	4,20E-01	1,745	3,636	3,40E-01	0	140303	473162	152045	918987	217956	211559	3695849	24451	127415	259775	668839	696413	19052	610565	242861	2,43
968	726,32117	38,666	0,822	12	3,361	2,763	2,10E-03	3,529	2,523	5,20E-04	1	110513	158298	240290	628619	365625	218509	518502	258400	92470	86859	125716	50415	101715	337638	237168	2,65
969	753,47272	60,875	0,815	9	2,968	5,489	7,80E-02	3,055	6,149	1,10E-01	0	9916	76195	945412	880475	922403	4347	348053	816025	84322	55210	27028	258156	280427	178918	3598	2,06
973	734,6217	47,847	0,875	4	8,525	12,371	2,40E-02	9,093	11,082	1,30E-02	0	78864	225472	445843	711784	168919	565453	1417739	1760102	46474	85181	76174	28422	97137	251370	58784	3,98
975	794,427	55,324	0	1	2,549	6,376	3,90E-01	2,817	7,483	3,70E-01	0	643005	30749	18926	24473	56035	24967	30524	27901	46107	52082	29302	30414	20454	29953	22809	3,42
976	596,82068	40,095	0,828	2	2,473	5,067	3,40E-01	3,019	6,692	3,20E-01	0	774986	29917	33598	77825	56373	48032	41186	74826	48197	63000	41704	42682	52327	33261	56143	3,39
980	544,28076	36,588	0,68	3	1,183	1,689	6,90E-01	1,236	1,816	6,20E-01	0	187344	389796	999961	496543	150617	769093	605865	376030	219597	216844	217093	963138	315795	335316	261301	2,96
982	709,94781	67,229	0	1	1,368	2,784	5,80E-01	1,393	3,556	6,50E-01	0	431469	450092	114235	912133	50248	831116	340146	36015	1120215	175996	265820	226156	29940	1346312	461386	2,44
986	877,52881	25,183	0	1	5,439	12,686	1,20E-01	4,851	10,271	1,00E-01	0	99425	643622	594191	268773	420583	1116698	891239	4775019	36305	176854	67658	175463	390774	309346	95257	5,96
988	587,3316	48,707	0	1	1,703	2,675	2,60E-01	1,994	3,475	2,10E-01	0	906317	124403	168842	263971	68052	194259	198128	202816	67011	205039	85701	250041	99415	176141	106152	3,21
991	473,27075	38,144	0,766	5	1,683	1,338	3,70E-02	1,825	1,244	7,90E-03	0	526609	1124175	932107	981980	1229240	811167	1230571	468172	439264	483937	1003259	265293	548269	1107121	811588	2,22
993	542,94873	43,544	0,788	12	3,816	4,017	3,80E-03	4,182	3,919	8,70E-04	1	279728	234496	262949	902840	568574	282443	845272	202091	117994	70925	184889	59744	123279	366396	304778	3,02
994	625,31287	27,287	0,828	21	2,702	2,489	1,30E-02	2,571	2,123	1,60E-02	1	200291	126298	156814	623867	233754	127854	405604	208051	65499	81632	116989	71150	116473	241224	117642	2,28
997	465,91656	21,975	0,846	9	2,176	2,643	7,60E-02	2,202	2,758	9,00E-02	0	778210	638963	496867	146981	61307	117608	86142	74231	157001	153670	292831	109134	194380	671928	875769	3
998	472,90167	33,524	0,763	4	1,66	3,061	3,40E-01	1,782	3,039	2,60E-01	0	49678	732798	152540	480972	90796	158819	85818	114114	49902	129124	83797	73094	270628	101032	47178	2,95
999	430,24728	20,53	0	1	0,78	1,013	4,70E-01	0,743	1,072	4,20E-01	0	664265	259398	97597	268826	82789	112973	266014	141330	243382	456843	130790	196008	328016	129444	90579	2,59
