# Supplementary material for: PTRF/Cavin-1 and MIF Proteins Are Identified as Non-Small Cell Lung Cancer Biomarkers by Label-Free Proteomics
Source: PLoS One. 2012 Mar 26;7(3):e33752. doi: 10.1371/journal.pone.0033752 (PMC3312891; doi:10.1371/journal.pone.0033752)
Supplement: Table S3 — PTRF and MIF MS2 spectra. (PDF) [file pone.0033752.s007.pdf]

Accession #	#AAs	MW [Da]	Description	ΣCoverage	Σ# Peptides
P14174	115	12468	Macrophage migration inhibitory factor [MIF_HUMAN]	9,57%	14
Q6NZI2	390	43450	Polymerase I and transcript release factor [PTRF_HUMAN]	2,82%	54

Sequence	IonScore	Exp Value	Charge	MH+ [Da]	ΔM [ppm]	Ions Matched	Annotation
PMFIVNTNVPR	41	3,50E-03	2	1287,69475	5,34	7/90	MIF
PMFIVNTNVPR	62	2,70E-05	2	1287,6884	0,41	8/90	MIF
PMFIVNTNVPR	43	2,30E-03	2	1287,68718	-0,54	9/90	MIF
PMFIVNTNVPR	74	1,80E-06	2	1287,69084	2,31	9/90	MIF
PMFIVNTNVPR	87	7,00E-08	2	1287,69267	3,73	9/90	MIF
PMFIVNTNVPR	77	8,80E-07	2	1287,69158	2,88	9/90	MIF
PMFIVNTNVPR	84	1,70E-07	2	1287,68852	0,51	9/90	MIF
PMFIVNTNVPR	66	1,10E-05	2	1287,68425	-2,81	9/90	MIF
PMFIVNTNVPR	70	4,60E-06	2	1287,68901	0,89	9/90	MIF
PMFIVNTNVPR	73	2,00E-06	2	1287,68877	0,7	9/90	MIF
PMFIVNTNVPR	43	2,10E-03	2	1287,69426	4,96	9/90	MIF
PMFIVNTNVPR	71	3,00E-06	2	1287,6884	0,41	9/90	MIF
PMFIVNTNVPR	69	5,00E-06	2	1287,68926	1,08	9/90	MIF
PMFIVNTNVPR	84	1,90E-07	2	1287,68999	1,64	9/90	MIF
SLKESEALPEK	46	1,00E-03	2	1230,66045	2,26	11/114	PTRF
SLKESEALPEK	44	1,70E-03	2	1230,66057	2,36	12/114	PTRF
SLKESEALPEK	27	8,70E-02	2	1230,66265	4,04	14/114	PTRF
SLKESEALPEK	47	8,80E-04	2	1230,66045	2,26	8/114	PTRF
SLKESEALPEK	53	2,20E-04	2	1230,66057	2,36	8/114	PTRF
SLKESEALPEK	49	5,20E-04	2	1230,65886	0,97	8/114	PTRF
SLKESEALPEK	39	5,20E-03	2	1230,66057	2,36	8/114	PTRF
SLKESEALPEK	56	1,10E-04	2	1230,65923	1,26	8/114	PTRF
SLKESEALPEK	49	5,10E-04	2	1230,65996	1,86	9/114	PTRF
SLKESEALPEK	56	1,10E-04	2	1230,6602	2,06	9/114	PTRF
SLKESEALPEK	58	7,10E-05	2	1230,65984	1,76	9/114	PTRF
SLKESEALPEK	58	7,60E-05	2	1230,65935	1,36	9/114	PTRF
SLKESEALPEK	59	5,50E-05	2	1230,6602	2,06	9/114	PTRF
SLKESEALPEK	56	1,00E-04	2	1230,65947	1,46	9/114	PTRF
SLKESEALPEK	51	3,50E-04	2	1230,65996	1,86	9/114	PTRF
SLKESEALPEK	42	2,80E-03	2	1230,65911	1,17	9/114	PTRF
SLKESEALPEK	35	1,60E-02	2	1230,65788	0,17	9/114	PTRF
SLKESEALPEK	56	1,20E-04	2	1230,66057	2,36	9/114	PTRF
SLKESEALPEK	59	5,80E-05	2	1230,65935	1,36	9/114	PTRF
SLKESEALPEK	58	7,50E-05	2	1230,65947	1,46	9/114	PTRF
SLKESEALPEK	58	6,90E-05	2	1230,65874	0,87	9/114	PTRF
SLKESEALPEK	62	3,10E-05	2	1230,65911	1,17	9/114	PTRF
SLKESEALPEK	47	8,80E-04	2	1230,66008	1,96	9/114	PTRF
SLKESEALPEK	58	7,70E-05	2	1230,65923	1,26	9/114	PTRF
SLKESEALPEK	59	5,70E-05	2	1230,66057	2,36	9/114	PTRF
SLKESEALPEK	45	1,50E-03	2	1230,65935	1,36	9/114	PTRF
