# Supplementary material for: PTRF/Cavin-1 and MIF Proteins Are Identified as Non-Small Cell Lung Cancer Biomarkers by Label-Free Proteomics
Source: PLoS One. 2012 Mar 26;7(3):e33752. doi: 10.1371/journal.pone.0033752 (PMC3312891; doi:10.1371/journal.pone.0033752)
Supplement: Table S4 — Peptide Mass Fingerprint and Protein Identification settings. (DOC) [file pone.0033752.s008.doc]

**Peptide Mass Fingerprint and Protein Identification settings.**

1. General Settings:

-----------------------------

Precursor Selection: Use MS1 Precursor

Unrecognized Charge Replacements: Automatic

Unrecognized Mass Analyzer Replacements: ITMS

Unrecognized MS Order Replacements: MS2

Unrecognized Activation Type Replacements: CID

Unrecognized Polarity Replacements: +

2. Scan Filters:

-----------------------------

Lower RT Limit: 13

Upper RT Limit: 0

Min. Precursor Mass: 350 Da

Max. Precursor Mass: 5000 Da

3. Scan Event Filters:

-----------------------------

MS Order: Is MS2

Scan Type: Is Full

4. Peak Filters:

-----------------------------

S/N Threshold: 3

5. Spectrum Properties Filter:

-----------------------------

Total Intensity Threshold: 0

Minimum Peak Count: 1

------------------------------------------------------------------------------

Processing node 2: Mascot

------------------------------------------------------------------------------

1. Input Data:

-----------------------------

Protein Database: SwissProt

Enzyme Name: Trypsin

Maximum Missed Cleavage Sites: 2

Instrument: ESI-FTICR

Taxonomy: . . . . . . . . . . . . Mammalia (mammals)

1.1 Peptide Scoring Options:

-----------------------------

Peptide Cut Off Score: 10

1.2 Protein Scoring Options:

-----------------------------

Use MudPIT Scoring: True

Protein Cut Off Score: 20

2. Decoy Database Search:

-----------------------------

Decoy Search: True

Target FDR (Strict): 0.01

Target FDR (Relaxed): 0.05

3. Tolerances:

-----------------------------

Precursor Mass Tolerance: 15 ppm

Fragment Mass Tolerance: 0.9 Da

Use Average Precursor Mass: False

4. Dynamic Modifications:

-----------------------------

1. Dynamic Modification: Oxidation (M)

2. Dynamic Modification: Phospho (ST)

3. Dynamic Modification: Phospho (Y)
